# Origin and composition of three heterolithic boulder- and cobble-bearing deposits overlying the Murray and Stimson formations, Gale Crater, Mars

**DOI:** 10.1016/j.icarus.2020.113897

**Published:** 2020-06-06

**Authors:** Roger C. Wiens, Kenneth S. Edgett, Kathryn M. Stack, William E. Dietrich, Alexander B. Bryk, Nicolas Mangold, Candice Bedford, Patrick Gasda, Alberto Fairen, Lucy Thompson, Jeff Johnson, Olivier Gasnault, Sam Clegg, Agnes Cousin, Olivier Forni, Jens Frydenvang, Nina Lanza, Sylvestre Maurice, Horton Newsom, Ann Ollila, Valerie Payré, Frances Rivera-Hernandez, Ashwin Vasavada

**Affiliations:** a Los Alamos National Laboratory, Los Alamos, NM, USA; b Malin Space Science Systems, San Diego, CA, USA; c Jet Propulsion Laboratory, California Institute of Technology, Pasadena, CA, USA; d Department of Earth and Planetary Science, University of California–Berkeley, Berkeley, CA, USA; e Laboratoire de Planétologie et Géodynamique, UMR 6112 CNRS, Université Nantes, Université d’Angers, Nantes, France; f Lunar and Planetary Institute, Houston, TX, USA; g Centro de Astrobiologia (CSIC-INTA), Madrid, Spain; h Planetary and Space Science Centre, University of New Brunswick, Fredericton, New Brunswick, Canada; i Johns Hopkins University Applied Physics Laboratory, Laurel, MD, USA; j Université de Toulouse, UPS-OMP, Toulouse, France; k Institut de Recherche en Astrophysique et Planéetologie, CNRS, UMR 5277, Toulouse, France; l University of Copenhagen, Copenhagen, Denmark; m Institute of Meteoritics, University of New Mexico, Albuquerque, NM, USA; n Earth, Environmental, and Planetary Sciences, Rice University, Houston, TX, USA; o Department of Earth Sciences, Dartmouth College, Hannover, NH, USA; p Department of Astronomy, Cornell University, Ithaca, NY, USA

**Keywords:** Gale crater, Heterolithic unit, Curiosity rover, Stimson formation, Murray formation, Greenheugh pediment

## Abstract

Heterolithic, boulder-containing, pebble-strewn surfaces occur along the lower slopes of Aeolis Mons (“Mt. Sharp”) in Gale crater, Mars. They were observed in HiRISE images acquired from orbit prior to the landing of the Curiosity rover. The rover was used to investigate three of these units named Blackfoot, Brandberg, and Bimbe between sols 1099 and 1410. These unconsolidated units overlie the lower Murray formation that forms the base of Mt. Sharp, and consist of pebbles, cobbles and boulders. Blackfoot also overlies portions of the Stimson formation, which consists of eolian sandstone that is understood to significantly postdate the dominantly lacustrine deposition of the Murray formation. Blackfoot is elliptical in shape (62 × 26 m), while Brandberg is nearly circular (50 × 55 m), and Bimbe is irregular in shape, covering about ten times the area of the other two. The largest boulders are 1.5–2.5 m in size and are interpreted to be sandstones. As seen from orbit, some boulders are light-toned and others are dark-toned. Rover-based observations show that both have the same gray appearance from the ground and their apparently different albedos in orbital observations result from relatively flat sky-facing surfaces.

Chemical observations show that two clasts of fine sandstone at Bimbe have similar compositions and morphologies to nine ChemCam targets observed early in the mission, near Yellowknife Bay, including the Bathurst Inlet outcrop, and to at least one target (Pyramid Hills, Sol 692) and possibly a cap rock unit just north of Hidden Valley, locations that are several kilometers apart in distance and tens of meters in elevation. These findings may suggest the earlier existence of draping strata, like the Stimson formation, that would have overlain the current surface from Bimbe to Yellowknife Bay. Compositionally these extinct strata could be related to the Siccar Point group to which the Stimson formation belongs.

Dark, massive sandstone blocks at Bimbe are chemically distinct from blocks of similar morphology at Bradbury Rise, except for a single float block, Oscar (Sol 516). Conglomerates observed along a low, sinuous ridge at Bimbe consist of matrix and clasts with compositions similar to the Stimson formation, suggesting that stream beds likely existed nearly contemporaneously with the dunes that eventually formed the Stimson formation, or that they had the same source material. In either case, they represent a later pulse of fluvial activity relative to the lakes associated with the Murray formation.

These three units may be local remnants of infilled impact craters (especially circular-shaped Brandberg), decayed buttes, patches of unconsolidated fluvial deposits, or residual mass-movement debris. Their incorporation of Stimson and Murray rocks, the lack of lithification, and appearance of being erosional remnants suggest that they record erosion and deposition events that post-date the exposure of the Stimson formation.

## 1 Introduction

**Fig. 1:**
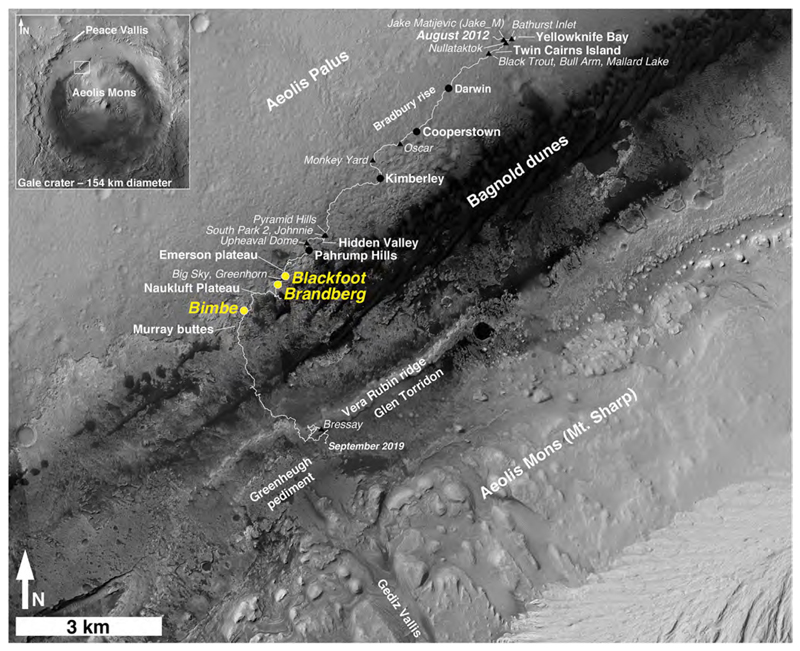
The Curiosity rover field site occurs on the lower northwest slope of the 5-km-high Aeolis Mons (Mt. Sharp), a mountain of predominantly stratified sediments in Gale crater. Gale is a ~154 km diameter impact structure. The white trace indicates the rover traverse from landing until September 2019. The inset shows the location of the field site within Gale crater. The three heterolithic units studied by the rover are indicated by filled circles. Waypoints are indicated by black circles. Targets mentioned in the text that are outside of the heterolithic units are indicated by triangles. Other features mentioned in the text are also indicated.

Since the Mars Science Laboratory (MSL) rover, Curiosity, landed on Mars in 2012, it has been used to explore the geologic record preserved within Gale (Fig. 1), a 154 km diameter impact crater located at the boundary between heavily cratered ancient highlands and younger, northern lowlands. Through investigation of Gale’s ancient rock record—considered to be younger than 3.8 Ga and generally older than 3.2 Ga (Thomson et al., 2011; Le Deit et al., 2013)—the MSL team seeks to characterize the nature and evolution of early Martian environments and to understand their potential habitability (Grotzinger et al., 2013; Grotzinger et al., 2015).

Here we present ground-based observations regarding a puzzling type of geological unit first observed prior to landing. Using ~25 cm/pixel High Resolution Imaging Science Experiment (HiRISE; McEwen et al., 2007) images acquired from the orbiting Mars Reconnaissance Orbiter (MRO), the team identified a suite of isolated features ranging in size from 10s to 100s of meters across that occur along the lower northern slopes of Aeolis Mons (informally, Mt. Sharp), the 5-km-high stratified mountain in Gale (Fig. 1). The strata of Aeolis Mons are sedimentary (Malin and Edgett, 2000; Anderson and Bell, 2010; Milliken et al., 2010). The puzzle was whether these isolated features are part of the sedimentary rock record (i.e., lithified units) or are younger, unconsolidated deposits.

These landforms exhibit a variety of shapes ranging from circular, elliptical, to irregular, but in HiRISE images all are relatively featureless in texture (at ~25 cm/pixel scale) and are of a uniform, intermediate to dark tone. In addition to these attributes, the landforms also contain concentrations of boulders to >1 m in size. Some of the boulders seen in HiRISE images exhibit light tones; others are dark toned. Prior to encountering these units, it had been suggested that the apparent light-toned rocks are from the eolian lithology on upper Mt. Sharp (Milliken et al., 2010), or that they could be remnants of landslide or glacial till deposits. The team preliminarily interpreted these landforms to be depositional units because of their boulder-rich nature. These “heterolithic deposits” or “blocky units” are spatially distinct, span a ~200 m elevation range along the lower northern slopes of Aeolis Mons, and occur across a lateral distance of ~10 km. Although most of the heterolithic units are in direct contact with the lowermost exposed unit of Aeolis Mons, the Murray formation, several examples overlie the Stimson formation, which unconformably overlies an erosional paleoslope cut through the Murray formation on the lower portion of Mt. Sharp (Banham et al., 2018; Watkins et al., 2016).

To understand whether the heterolithic deposits are part of the ancient rock record in Gale crater, or whether they are modern, unconsolidated materials like the nearby eolian dunes, the MSL team used the tools and instruments of the Curiosity rover to investigate three of them that were encountered along the rover traverse (Fig. 1). In this paper we describe their setting, morphology, lithology, and compositional relationships using data acquired from orbit and on the ground. Based on these observations, we determine the stratigraphic context of the heterolithic deposits relative to the major geologic units investigated by the Curiosity rover, and discuss potential depositional origins and processes for these deposits. This work focuses exclusively on the three units studied by Curiosity, though many more exist in the area at the base of Mt. Sharp.

### 1.1 Geological context

Curiosity landed on Bradbury Rise, at the distal end of the alluvial fan produced by Peace Vallis, a fluvial channel that descends from the crater rim (Fig. 1 inset; Palucis et al., 2014). Rocks of the Bradbury group include loose gravels, conglomerate beds, and sandstones deposited in a fluvial setting (Williams et al., 2013; Grotzinger et al., 2015). Upon reaching the Pahrump Hills around Sol 750, Curiosity entered the Murray formation (Grotzinger et al., 2015) of the Mt. Sharp group. This primarily lacustrine formation has been eroded to expose over 300 m in vertical extent starting at Pahrump Hills and extending up at least through Vera Rubin ridge (VRR; Fraeman et al., 2020) and Glen Torridon (Fig. 1). The slope of the eroded Murray formation forms the base of Mt. Sharp. Draping over this erosional surface and forming patchy outcrops overlying the Murray is the Stimson formation, considered to be part of the Siccar Point group (Fraeman et al., 2016; Banham et al., 2018; Watkins et al., 2016). It consists of dark-gray sandstones containing cross-bedding ~1 m thick with cross laminations. It has a bimodal grain size centering on 250 μm and 710 μm (Banham et al., 2018). The Stimson formation was interpreted to be eolian (Banham et al., 2018). Lithification of the Stimson followed by canyon incision led to formation of the ~7–17 meter-high Murray buttes (Fig. 1 and Supplemental material) in which the Stimson formation is exposed as buttes and mesas over the underlying Murray formation (Banham et al., 2019).

In addition to the Murray and Stimson formations, Curiosity encountered three heterolithic units (Fig. 1): Blackfoot (encountered by Curiosity on sols 1094–1104), Brandberg (sols 1158–1160), and Bimbe (sols 1400–1410), characterized by unconsolidated deposits of pebbles, cobbles and boulders that overlie the Murray formation and, in at least one case, the Stimson formation. These units are clearly different from the surrounding surface exposures. Dark-toned, massive float rocks in particular are unique to these units relative to the surrounding surface, which have very few float rocks aside from those that clearly belong to the respective Murray and Stimson formations or are meteorites (Meslin et al., 2017). These three units occur between elevations of −4434 and − 4419 m (relative to the Martian datum) within 1.2 km of each other and within ~0.5 km of a discontinuous scarp that marks a topographic transition between the flat plains (Aeolis Palus) and Mt. Sharp (Fig. 1).

The units are distinct from boulder and cobble deposits that occur on the slopes of mounds, buttes, mesas, and scarps in the region. In the latter cases, usually an intact, erosion-resistant cap rock (e.g., Stimson formation, in the case of Murray Buttes) overlies exposures of Murray-formation rock. It is also important to note that there are few to zero cobbles and boulders composed of fragments of these overlying rocks sitting on Murray-formation bedrock exposures > 50 m (and usually not >10 m) from the base of the buttes.

## 2 Methods

### 2.1 Imaging

All HiRISE images acquired through 1-October-2017 of Blackfoot (24 images), Brandberg (23 images), and Bimbe (28 images) were used to observe the distribution pattern and relative tone of observable boulders. Typically, these are boulders ≥ 0.45 m in size; boulders smaller than ~1 m in size can be identified if they have a tone that contrasts with surroundings and/or cast a shadow. In addition, we illustrate our observations using the Calef and Parker (2016) mosaic of HiRISE images of the MSL investigation site compiled as a base map with the rover traverse overlain. Topographic information comes from a digital elevation model (DEM) assembled by Parker and Calef (2016) using HiRISE stereo pair images and correlated with Mars Global Surveyor (MGS) Mars Orbiter Laser Altimeter (MOLA) topographic measurements, referenced to the Martian datum per the approach outlined by Kirk et al. (2008).

Nearly all of Curiosity’s cameras were used in this investigation. They consist of redundant dual pairs of left and right stereo Navigation cameras (Navcams; Maki et al., 2012) and front and rear Hazard cameras (Hazcams; Maki et al., 2012); two Mast cameras, one on the right side of the mast, with a 100 mm focal length for long-range imaging, and one on the left side with a 34 mm focal length for a larger field of view (Mastcam-34 and Mastcam-100; Malin et al., 2017); a Mars Descent Camera (MARDI; Malin et al., 2017); a Remote Micro Imager (RMI; Maurice et al., 2012; Le Mouelic et al., 2015); and a Mars Hand Lens Imager on the rover arm (MAHLI; Edgett et al., 2012). Imaging via Curiosity’s cameras occurs in two modes: (1) mono- and stereo-context imaging and (2) high-resolution or close-up imaging. The rover’s Mastcams, Navcams, Hazcams, and MARDI provide contexts at a variety of scales as a function of distance from the rover. The RMI and MAHLI provide close-up photography. As part of ChemCam (Wiens et al., 2012; Maurice et al., 2012), the RMI provides panchromatic images with a spatial resolution of ~100 μm within 2.5 m distance. Typical MAHLI macrophotography provides color images over a 15–100 μm/pixel range (Yingst et al., 2016).

### 2.2 Boulder and cobble lithology interpretation

Stereo context imaging provides information on boulder and cobble dimensions that are generally accurate to ±1 cm (Maki et al., 2012). Boulder and cobble size classifications use the scheme of Blair and McPherson (1999) and Terry and Goff (2014) over the 6.4–409.6 cm size range: fine cobbles (6.4–12.8 cm), coarse cobbles (12.8–25.6 cm), fine boulders (25.6–51.2 cm), medium boulders (51.2–102.4 cm), coarse boulders (102.4–204.8 cm), very coarse boulders (204.8–409.6 cm). Grains smaller than cobbles and coarser than 4 mm are referred to as pebbles, while particles 2 to 4 mm are granules (Wentworth, 1922). A detailed approach to both the Curiosity rover instruments and rock classification is provided by Mangold et al. (2017).

Most of the boulders, cobbles, and pebbles observed at Blackfoot, Brandberg, and Bimbe were investigated only through contextual images obtained by MRO HiRISE and Curiosity’s Mastcams, Navcams, and Hazcams. Additional context images were obtained by MARDI and MAHLI (wheel inspection and landscape images), particularly when the rover drove across Blackfoot. A few boulders and cobbles were examined in greater detail using MAHLI for macrophotography; the Alpha Particle X-ray Spectrometer (APXS; Campbell et al., 2012) was used for geochemistry on one cobble at Blackfoot and two boulders at Bimbe. ChemCam RMI images and laser-induced breakdown spectroscopy (LIBS) were used to investigate three targets at Blackfoot, three targets at Brandberg, and 16 targets (several of which are on the same boulder) at Bimbe. As described below, the compositions derived from ChemCam LIBS and APXS are used in the interpretations of the boulders and cobbles.

Image interpretation of boulder and cobble lithology is based on experience gained from Curiosity’s exploration in Gale crater to date. All of the >350 m of observed in-place strata appear to be sedimentary rocks. Further, the nearly 5 km of stratigraphy above, on Mt. Sharp, are also interpreted to be dominated by sedimentary rock (e.g., Malin and Edgett, 2000; Anderson and Bell, 2010; Milliken et al., 2010). The strata encountered along the traverse include conglomerates interpreted to be fluvial sediments (Williams et al., 2013; Mangold et al., 2016); sand-stones interpreted to be fluvial, deltaic, and lacustrine (Grotzinger et al., 2013; Grotzinger et al., 2015; Rice et al., 2017); sandstones interpreted to be eolian (Banham et al., 2018); and rocks finer-grained than fine sand, interpreted largely to be lacustrine mudstones (e.g., Grotzinger et al., 2015). Clasts interpreted as being derived from igneous processes or perhaps impact-generated melts have been confined to coarse cobbles and smaller sizes (Williams et al., 2013; Sautter et al., 2014, 2015, 2016; Mangold et al., 2016; Cousin et al., 2017a); these are interpreted as having been liberated by rock decay processes from exposures of conglomerate and pebbly sandstone, likely transported in streams from the crater walls and rim (e.g., Williams et al., 2013; Sautter et al., 2016).

The experience in Gale crater includes observations of bedrock properties and landscape evolution that pertain to indicators of Blackfoot, Brandberg, and Bimbe boulder and cobble lithology as a function of color, shape, sedimentary texture (e.g., clastic, clast properties, fabric), and sedimentary structure (bedding, bedding plane features, soft sediment deformation). Concretions and nodules (Stack et al., 2014; Nachon et al., 2017; Wiens et al., 2017; Sun et al., 2019) as well as fracture-associated diagenetic features such as veins (Nachon et al., 2014, 2017; Kronyak et al., 2019) and alteration halos (Frydenvang et al., 2017; Yen et al., 2017) also occur in the sedimentary rocks of Gale; their presence in a boulder or cobble can also aid in lithological identification. Color is helpful because the majority of sandstones coarser than very fine sand observed in Gale are medium to dark gray (almost black). Conglomerates are generally gray with clasts of varied sizes from sand to small cobbles of various shades of gray from white to black. Mudstones and very fine sandstones which make up the majority of the stratigraphy encountered by Curiosity, occur mostly in various shades of medium to light-gray, purple-gray, and various shades of red.

Thus a first-order comparative approach emerges for lithologic interpretation of boulders and cobbles at Blackfoot, Brandberg, and Bimbe that are only seen in context images.

- Conglomerate cobbles and boulders have a gray matrix and abundant pebble clasts. In some cases, pebbles liberated from their surfaces litter the adjacent ground. Pebbles were normally interpreted as igneous from their coarse-grained or massive texture, at least on Bradbury Rise (e.g., Williams et al., 2013).
- Cobbles and boulders interpreted as sandstone are dark gray, can exhibit bedding structures and sand-, granule-, or minor pebble-sized clasts as viewed in the highest spatial resolution context images (e.g., Mastcam-100 data). Some of the dark-gray sandstones in Gale have concretions (like the object liberated from Stimson eolian sandstone studied by Van Bommel et al., 2017), fracture fills (veins), or fracture-parallel alteration “halos.”
- Mudstone cobbles and boulders are expected to be rare, given their poorer resistance to erosion. However, angular fine cobbles of mudstone do occur and have been identified along Curiosity’s traverse (e.g., Lamoose target noted by Morris et al., 2016, and a large number of dislodged slabs observed in the upper Murray formation below VRR, and smaller pebbles on the ridge itself). Fine laminae and diagenetic features, especially veins and concretions, are common in the mudstones in Gale (e.g., Sun et al., 2019). Color might actually be the most helpful clue, as none of the mudstones identified thus far have been as dark gray as the sandstones and their colors include reddish, golden brown, and purplish strata.

### 2.3 Elemental compositions and reflectance spectroscopy

ChemCam uses LIBS at distances of 2–7 m from the rover’s remote sensing mast to determine elemental chemistry (Wiens et al., 2012; Maurice et al., 2012). Laser pulses of 14 mJ energy and 5 ns duration are focused on observation points (0.3–0.6 mm diameter) on the targets. The first few laser pulses remove dust from the surface. Individual spectra from pulses 6–30 at each targeted point are averaged and processed to remove ambient-light background, noise, and electron continuum, leaving the atomic emission spectrum (Wiens et al., 2013). These are further processed through a calibration algorithm that normalizes the spectra, correcting for variable distance (Wiens et al., 2013), and yields the abundances of the major elements as oxide weight percent (Clegg et al., 2017). Trace elements Li, Rb, and Sr are processed by calibrating the area of an emission peak of the respective element, slightly revised from Payré et al. (2017).

Accuracies are estimated using the calibration algorithm on a separate test set of standards over a variety of compositions, yielding accuracies as functions of abundances for each major element and trace element. Precisions are determined by comparing repeated observations of onboard calibration targets carried out over a number of different days (Blaney et al., 2014) or by comparing repeated observations of a homogeneous lithologic unit such as the Sheepbed mudstone in Gale crater (Mangold et al., 2015). The latter is more realistic in that it is done over various instrument-to-target distances, but it is a worst-case measurement of precision, as it includes any heterogeneity that may exist in the bedrock at the scale of the beam. Overall, the two determinations of precision are comparable. Finally, for each observation point, the averaging of 25 spectra, and the resulting standard deviation, allows us to determine the stability of the composition within the evolving laser pit. This standard deviation is another measure of precision and is equivalent in its nature to the error reported with APXS observations. An exception is that, because the laser creates a depth profile into the target, the standard deviation includes the heterogeneity of the target over the depth of laser pulses 6 to 30. Normally this is not a significant consideration but sometimes the composition does change over the course of the 25 laser pulses, resulting in a higher standard deviation that is unrelated to instrument stability.

All ChemCam statistics, including abundances, accuracies, and standard deviations are provided in the data submitted to the Planetary Data System. Quantification limits for the trace elements Li, Rb, and Sr are 5, 26, and 96 ppm, respectively. While accuracies vary with abundances, observations presented here were generally within ±5, ±30, and ±150 ppm, respectively.

For all targets, Fe is not separated by oxidation state, but is computed as total iron as FeO (“FeO_T_”). The major elements are not normalized to 100% to allow for contributions from minor elements such as S, P, Cl, F, and H. In most cases the major-element total is in the mid-90s in wt%, but in a few cases the minor elements appear to comprise up to ~12 wt%.

APXS was used to analyze four targets: Badlands in the Blackfoot area and targets Sonneblom, Funda, and Zambezi at Bimbe (Sonneblom and Zambezi were on the same boulder). Funda was analyzed by a 4-point raster, while the other three targets were single analyses. Badlands, Zambezi, and Sonneblom were overnight integrations. The Dust Removal Tool was not used on any of the targets. The footprint of APXS is ~1.7 cm in diameter.

Johnson et al. (2015) demonstrated that radiance spectra acquired using ChemCam without the laser provides sufficient signal to allow measurement of reflected sunlight from soil and rock targets. Calibration of such data was accomplished by dividing the scene radiance spectrum by a calibration target radiance spectrum with known reflectance properties (Wiens et al., 2013). This provided an estimate of relative reflectance with uncertainties of <10% (Johnson et al., 2015). Typically, such passive observations were acquired after laser shots, which served to minimize dust contamination in the measurement’s field of view.

The rover’s interior mineralogical and geochemical laboratories, Chemistry and Mineralogy (CheMin) and Sample Analysis at Mars (SAM), were not used to investigate Blackfoot, Brandberg, and Bimbe.

## 3 Results

### 3.1 Blackfoot image observations

#### 3.1.1 Field site and stratigraphic position

**Fig. 2:**
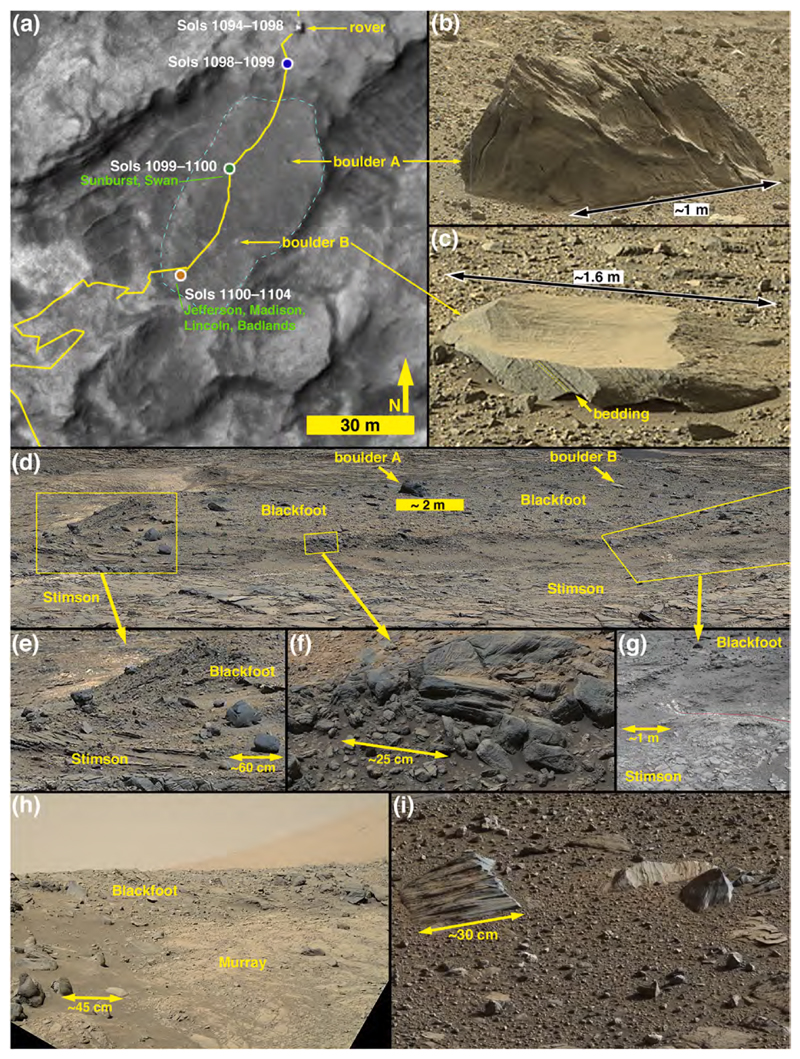
(a) The Curiosity rover and the Blackfoot heterolithic unit (within blue dashed outline) as imaged by HiRISE on 04 September 2015. Yellow trace indicates rover traverse, from north toward southwest. Locations of boulders A and B and the sites of two rover stops on Blackfoot are indicated. Locations of ChemCam targets Sunburst, Swan, Lincoln, Jefferson, Madison, and the APXS/MAHLI target Badlands, are indicated in green. (b) Boulder A is dark-toned when viewed from above; the rock is a dark gray sandstone and it has a surface that is rough at a centimeter scale. (c) Boulder B is light-toned when viewed from above (e.g. HiRISE images); the rock is a dark-gray sandstone; bedding is apparent; the skyward-facing surface is smooth at a centimeter scale and coated with eolian dust. (d) Blackfoot as seen from the north on Sol 1094 when the rover was parked at the position shown in (a). Boulders A and B and the locations of (e), (f), and (g) are indicated. (e) The north and east margins of the Blackfoot heterolithic unit overlie dark-gray, cross-bedded eolian Stimson-formation sandstones. (f) North edge of Blackfoot reveals the deposit in cross-section; here, it consists of a jumble of angular cobbles and pebbles; cobbles display a variety of orientations relative to their internal sedimentary structures (bedding). Dark-toned eolian sand has filled some of the gaps between stones. (g) Many of the open fractures which cut through Stimson-formation sandstones exhibit a light-toned, fracture-parallel, altered zone (a “halo;” see Frydenvang et al., 2017 and Yen et al., 2017). Here a fracture and associated halo follow the red trace and are abruptly truncated where covered by the Blackfoot heterolithic unit; this indicates that the fracture and fluids responsible for the “halo” alteration did not penetrate the Blackfoot deposit; i.e., one of the indicators that the deposit is not a lithic unit. (h) At its west-southwest end, the Blackfoot unit directly overlies mudstones of the Murray formation. (i) The cobbles and boulders of Blackfoot display a range of orientations, relative to their internal structure (bedding). Some of them protrude from the surface, others rest on the surface. (For interpretation of the references to color in this figure, the reader is referred to the web version of this article.)

Of the three heterolithic units that were investigated, Blackfoot was the only one that the Curiosity rover drove across. The route went southwest across the long dimension of Blackfoot between sols 1099 and 1104 (09–14 September 2015). Curiosity parked at two spots on Blackfoot (Fig. 2a) to briefly investigate the surface. ChemCam observed targets named Sunburst and Swan at the Sol 1099–1100 site and targets Jefferson, Madison, and Lincoln, at the Sol 1100–1104 site. APXS and MAHLI were deployed on Sol 1102 to study an angular, coarse cobble named Badlands at the Sol 1100–1104 parking spot. Early views of Blackfoot were acquired on Sol 1094 (04 September 2015) and later views were obtained from the Big Sky and Greenhorn drill sites (Fig. 1; see Yen et al., 2017) as late as Sol 1144 (25 October 2015). HiRISE captured an image of the Curiosity rover on Sol 1094 (Fig. 2a).

Blackfoot covers about 1730 m2 in a semi-elliptical area that has a long dimension of ~62 m oriented northeast-southwest and a short dimension of ~26 m perpendicular to that. It occurs between elevations −4434 and − 4432 m on the lower north-facing slope of Mt. Sharp. Excluding the largest boulder, the unit has <~1.1 m relief and its overall surface slopes upward ~1.2° toward the southwest (i.e., elevation gain of ~1.4 m over 62 m). At the northeast end, the gravels, cobbles, and boulders of which it is composed are in contact with the cross-bedded sandstone facies of the Stimson formation (Fig. 2d, e). At its southwest end it is in contact with mudstones of the Murray formation (Fig. 2h). The Blackfoot unit cross-cuts and bridges the pattern of parallel ridges (Banham et al., 2018) separated by troughs > 4 m deep that are exhibited by the underlying Stimson and Murray-formation rocks (Fig. 2a, Supplementary Fig. 1-1). Its topography and contact relations with underlying bedrock suggest a near-uniform thickness of the order of a few tens of centimeters. No intact rock unit overlies Blackfoot; it is not emergent from beneath superposed rock strata.

#### 3.1.2 Physical sedimentology of the Blackfoot deposit

The surface of Blackfoot is pebbly with scattered occurrences of cobbles and boulders of a variety of sizes, shapes, and orientations relative to their internal bedding structures (Fig. 2f, i). Some of the boulders and cobbles are resting on the surface of Blackfoot, while others protrude from the deposit. The loose, pebbly surface includes a few minor superimposed patches of unconsolidated eolian sand. The largest boulders are ~1.5–2.5 m in size; boulders of sizes ~0.45 to ~1.8 m can also be observed in the highest quality HiRISE images of Blackfoot (e.g., PSP_009294_1750 and ESP_018854_1755), as well as the images acquired during the rover’s visit to the area. Most—but, importantly, not all—boulders and cobbles are clustered on the downward slopes around the unit margins; some of these clasts have apparently moved down onto nearby, subjacent rock surfaces.

The northeast end of Blackfoot exhibits an exposure that gives a glimpse of its internal sedimentary structure (Fig. 2f). The framework clasts in this few tens-of-centimeters-thick exposure are angular to subrounded pebbles and cobbles; matrix clasts — i.e., grains finer than pebbles in the interstices between pebbles and cobbles — are not resolved in the highest resolution images acquired. Sorting in this apparent exposure is poor and laminae or beds within individual cobbles are randomly oriented. Although the subjacent Stimson and Murray-formation rocks are cut by fractures (some of which are filled with minerals, i.e., veins), the Blackfoot material is not; furthermore, some fractures in Stimson-formation rocks terminate at the boundary with the Blackfoot unit (Fig. 2g). Particle size analysis of Blackfoot (Supplementary material, Section 2) indicates a median size (D50) of 16 mm, and 84th percentile (D84) of 40 mm and a 16th percentile (D16) of 7 mm (Supplementary material, Section 2, Supplementary Fig. 2-3).

#### 3.1.3 Evidence of lithologic diversity

The lithologic diversity of Blackfoot was suspected for several years before the rover arrived, based on mapping during traverse planning. This notion was due to the tone of the two largest boulders at Blackfoot relative to the surrounding terrain as seen in HiRISE images. As labeled in Fig. 2a, boulder A has a dark tone and boulder B has a light tone in this perspective from orbit. As seen from the ground, the only relatively light-toned rocks encountered along the traverse to that point were light-gray mudstones of poorer erosion resistance (e.g., Grotzinger et al., 2013; Grotzinger et al., 2015; Morris et al., 2016; Schieber et al., 2016). In addition to these few light-toned rocks, light-toned fracture-associated halos occur in the Stimson formation (Frydenvang et al., 2017; Yen et al., 2017), but the affected portion of these sandstones is small relative to the size of boulder B. In addition, white vein minerals, interpreted based on ChemCam and APXS observations to be calcium sulfates, are abundant throughout Curiosity’s field site (Nachon et al., 2014, 2017; Kronyak et al., 2019). Clasts formed from the breakdown of exposed, eroding veins along the traverse have been of pebble size and smaller (e. g., Newsom et al., 2018).

The light-toned (orbital perspective) boulder B was imaged on the ground using the Mastcams from four different locations on sols 1094, 1098, 1100, and 1144 (e.g., Fig. 2c), and it was also captured in a MAHLI rover wheel inspection image from Sol 1102, as well as in engineering camera data. The boulder’s dimensions are approximately 2.7 by 1.6 m. The Mastcam images provide eight important observations about boulder B: (1) the rock is actually not light-toned as seen from the ground; it is dark gray, (2) it is angular, (3) it is tabular with a relatively flat skyward-facing surface, (4) its skyward-facing surface is coated with brownish-orange eolian dust, (5) its skyward-facing surface is smooth at centimeter scale, (6) it exhibits sedimentary structure in the form of bedding, (7) the bedding thickness is <1 cm (i.e., of the order of a few millimeters), and (8) the bedding is at an angle of about 50° relative to the top of the boulder and relative to the surface on which the boulder rests. On the basis of bedding and color, boulder B is interpreted to be a sandstone, similar to other sandstones observed in the area and in Gale crater in general. The light tone observed from orbit (Fig. 2a), and also to some extent as observed from a distance with rover cameras, is entirely the result of the combination of a dust coating and a skyward-facing surface that is smooth at centimeter and perhaps millimeter scales.

Boulder A was imaged using Curiosity’s Mastcams and engineering cameras from four different locations on sols 1094, 1098, 1100, and 1144 (e.g., Fig. 2b). Boulder A differs from boulder B; it is smaller (~1.2 m across), but stands higher above the surface owing to its greater sphericity. Like boulder B, it is dark gray in color, but it is rougher at a centimeter scale. Boulder A, too, has dust on its skyward-facing surfaces, but the combination of shape and centimeter-scale roughness renders it dark-toned when viewed from above in HiRISE images.

**Fig. 3:**
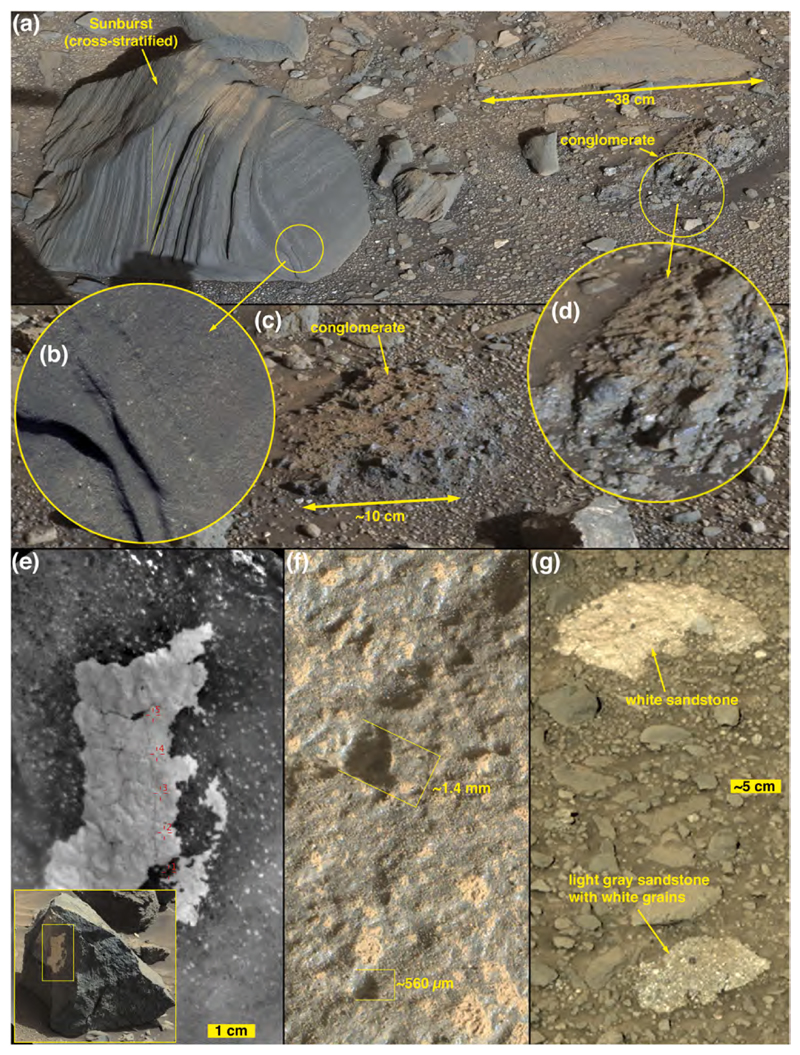
(a) Typical Blackfoot surface, with scattered cobbles, small boulders, pebbles, and sand of various orientations (relative to internal sedimentary structure, such as bedding), colors, and lithologies. Two key examples are highlighted: A cross-stratified sandstone boulder (ChemCam target named Sunburst) and a conglomeratic cobble. (b) Dark-gray sandstone target named Sunburst. White and light-gray sand grains are readily distinguished because of their contrast relative to the majority of grains, which are dark gray. (c) Example of a light-gray conglomeratic cobble, protruding from the surface. The orange-brown material on part of this stone is eolian dust. (d) Pebbles in an otherwise light-gray sandy matrix in a conglomeratic stone protruding from the surface at Blackfoot. (e) ChemCam target Madison; inset shows the image location on the rock face. The large, light-toned feature consists of the remains of a fracture wall vein composed of calcium sulfate. More importantly, the image shows light-toned sand grains, which contrast with the bulk of dark-toned sand grains (much more difficult to see) in this rock. Madison is a dark-gray sandstone. (f) APXS/MAHLI target, Badlands. This stone, too, is a dark-gray sandstone. Unlike Madison and Sunburst, Badlands has no light-toned sand grains; they are all dark gray. The sizes of two larger grains are indicated; most grains are smaller and difficult to distinguish from each other and their intergranular cement. Brownish-orange patches are depressions (some of them, perhaps, may be sockets from which sand grains have been removed) containing eolian dust. (g) Two examples of the light-toned sandstone or pebbly conglomerate fragments which occur at Blackfoot. (For interpretation of the references to color in this figure, the reader is referred to the web version of this article.)

The images acquired during Curiosity’s traverse across Blackfoot show the range of boulder and cobble lithologies. Generally, on the basis of color, tone, texture, and sedimentary structure, the boulders can all be interpreted as sandstones. Some of the cobbles can also be interpreted as likely sandstones, on the same basis, but some cobbles are pebbly conglomerates or pebbly sandstones (Fig. 3). The lithology of pebble clasts and smaller grains are not interpreted here owing to insufficient spatial resolution in the images acquired.

Fig. 3 shows various examples of the rocks interpreted to be conglomerates and sandstones at Blackfoot. Mastcam and RMI images of select targets, such as Swan, Sunburst, Lincoln, Jefferson, and Madison show that sand grains are present in these rocks (Fig. 3b, e), all of which are interpreted to be dark gray, erosion-resistant sandstones. Their individual characteristics vary; for example Madison and Sunburst include light-toned sand grains (Fig. 3b, e) and Sunburst exhibits cross-stratification (Fig. 3a). MAHLI images of the rock target Badlands also show sand grains (Fig. 3f). Some of the rocks observed at Blackfoot are light gray to white in color (Fig. 3g); some of these might have been eroded from fracture-associated altered sandstones that occur in the Stimson formation (see Frydenvang et al., 2017; Yen et al., 2017).

None of the boulders and cobbles observed at Blackfoot resemble or are lithic fragments of the Murray formation. As well as can be determined from the images acquired by the Mastcams, RMI, Navcams, Hazcams, MARDI, and MAHLI, none are as finely (<1 mm) laminated as typical Murray-formation rock, nor are they of one of the colors—light grays, grayish purples, brownish and yellowish reds—observed in Murray-formation strata.

In summary, Blackfoot is a relatively thin accumulation of poorly sorted pebbles to boulder-size clasts; the angular to well-rounded pebble-sized lithic fragments appear to serve as matrix to the coarser rocks. The boulders and cobbles exhibit random orientations relative to their internal sedimentary structure (bedding); some of them protrude from the deposit and others rest on its surface. The deposit overlies both Stimson and Murray-formation rocks and is not lithified. The surface is largely a pebbly lag with very little superimposed eolian dust and sand. Boulder and cobble shapes include angular clasts; these are suggestive that they were not transported far from their bedrock source. The boulders are generally dark-gray apparent sandstones; the gray conglomerates are a sub-set of a population of otherwise dark gray (and a few light gray to white) sandstone cobbles.

### 3.2 Brandberg image observations

#### 3.2.1 Field site and stratigraphic position

**Fig. 4:**
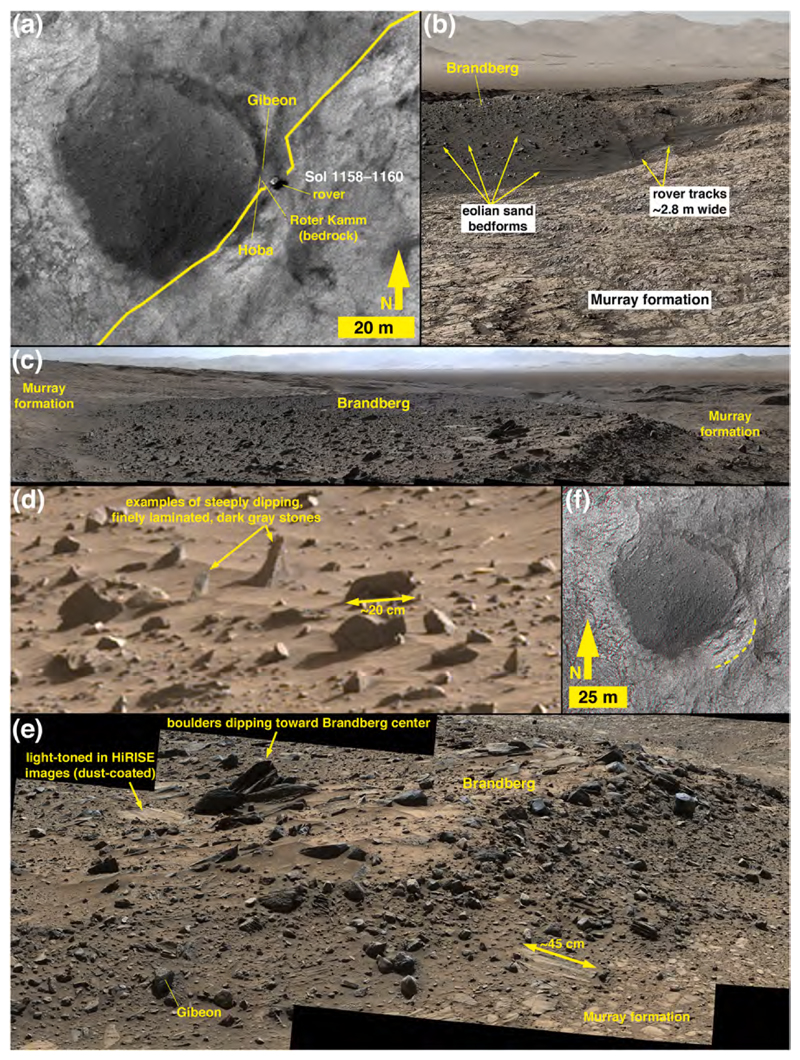
(a) Brandberg and the Curiosity rover as viewed by HiRISE on Sol 1159. Yellow trace indicates the rover traverse; drive direction was from northeast to southwest. ChemCam targets are indicated by name. (b) Eastern Brandberg. In this scene, Brandberg is downhill from the rover; the near side of Brandberg forms a shallow depression. Rover tracks provide scale. Eolian bedforms explain the faint lineated texture observable in HiRISE images. (c) Brandberg as viewed from the northeast. (d) Examples of steeply dipping cobble-sized angular clasts in Brandberg. (e) Examples of clast variety in northeastern Brandberg. The largest boulder-sized clasts dip toward the center of the circular landform. Boulders with relatively flat, skyward-facing surfaces are coated with dust and thus appear light-toned when seen from above in HiRISE images. The ChemCam target, Gibeon, is indicated. (f) Red/cyan stereo pair anaglyph produced from two HiRISE images; the dashed yellow arc indicates a ridge that might be an indicator that Brandberg occurs within the eroded remains of an impact structure. (For interpretation of the references to color in this figure, the reader is referred to the web version of this article.)

Brandberg is a nearly circular (~50 × 55 m) landform. The MSL team parked the rover next to the eastern edge of Brandberg on Sol 1158 for a limited investigation. The Navcams, Hazcams, Mastcams, and ChemCam were all used to examine nearby cobbles and boulders of Brandberg from this vantage point over sols 1158–1160 (09–11 November 2015). HiRISE captured an image of Curiosity parked at the site on Sol 1159, 10 November 2015 (Fig. 4a). Brandberg was also visible in Mastcam mosaics acquired during the drive toward and away from Brandberg over the sol 1115–1163 period (25 September–14 November 2015).

Covering about 2300 m^2^, Brandberg occurs between elevations −4435 and − 4432 m on the lower north-facing slope of Mt. Sharp. Not only is it nearly circular, an arcuate ridge of about 1–2 m height occurs about 15–20 m to its east (Fig. 4f). A Sol 1160 Mastcam mosaic (sequence mcam05248) shows that the ridge is composed of purple-ish Murray-formation bedrock. As viewed in planform in HiRISE images, Brandberg has a surface speckled with light- and dark-toned boulders (Fig. 4a). The overall surface is also dark-toned, similar in HiRISE images to nearby eolian dunes such as the Namib and High dunes explored via Curiosity (Bridges and Ehlmann, 2018) after departing Brandberg. The HiRISE views of Brandberg also show that it has a discontinuous, meter-scale parallel ridged texture that runs approximately northeast-southwest (Fig. 4a). In HiRISE images and all of the acquired Mastcam and Navcam mosaics of Brandberg, this unit is seen to be elevated above and in contact with Murray-formation rocks, all around its circumference. It does not contact the Stimson formation and there are no overlying rock units. Supplementary Fig. 1-2 shows that Brandberg and Blackfoot occur at the same elevation and have a similar thickness.

#### 3.2.2 Physical sedimentology of the Brandberg deposit

The surface of Brandberg exhibits scattered boulders, cobbles, and pebbles, interspersed with dark-gray, windblown sand and granules (Fig. 4b–e). The sands form tails in the lee of boulders and cobbles (Fig. 4b), creating the pattern of northeast-southwest oriented lineations observed in HiRISE images. Some of the boulders and cobbles are resting on the surface of Brandberg, but many protrude from within the deposit (Fig. 4d, e).

**Fig. 5:**
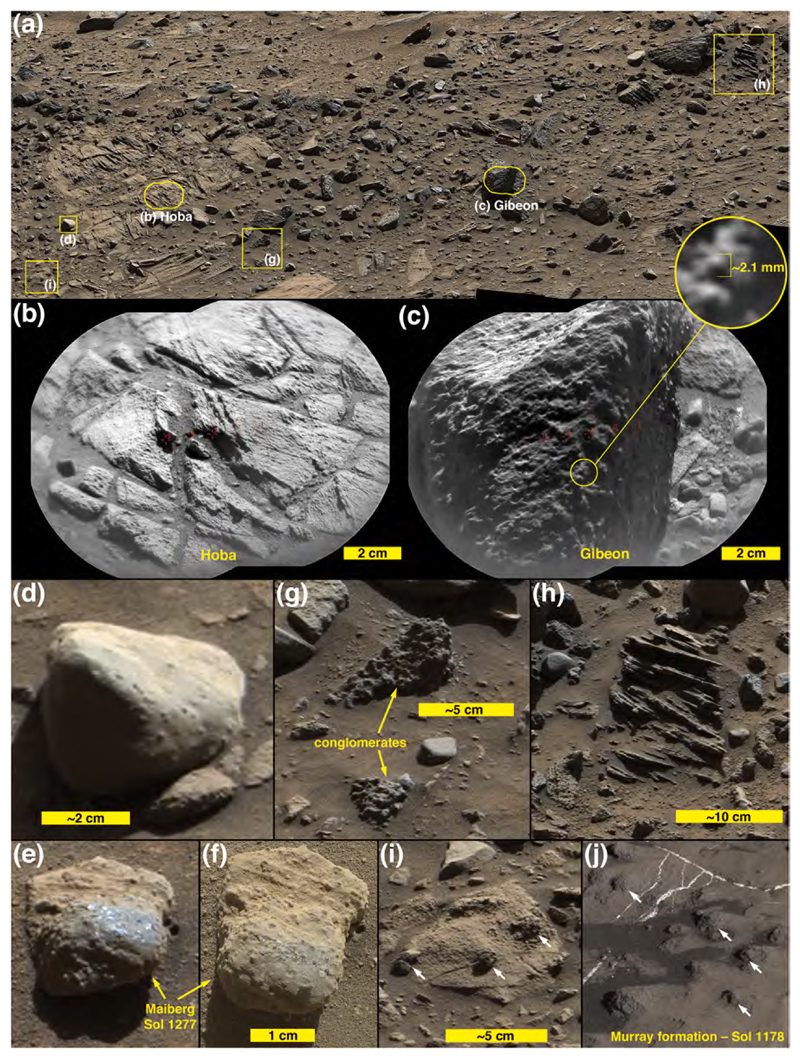
(a) Sol 1160 view of the variety of boulders, cobbles, and pebbles which compose the Brandberg deposit. Outlines indicate locations of panels b, c, d, g, h, and i. (b) Dust-coated, fractured Murray-formation lithic clast target named Hoba. (c) Dark-gray sandstone cobble target named Gibeon. The inset shows enlargement of a single grain or concretion, representative of the larger grains (or typical concretion size) in this rock, ~2 mm. (d) Sandstone with concretions. (e) Example sandstone with concretions (target named Maiberg) liberated from Stimson-formation bedrock on the east side of Naukluft Plateau, imaged on Sol 1277; shown here for comparison. (f) Sedimentary structure and texture details of Maiberg. (g) Examples of candidate, rare conglomeratic stones at Brandberg. (h) Example of angular, cobble-sized fragment of cross-bedded sandstone. (i) Example lithic fragment of Murray-formation bedrock incorporated into the Brandberg deposit; white arrows indicate erosion-resistant concretions; compare with (j): Example of intact Murray-formation bedrock south of Brandberg which contains similar concretions (white arrows).

Overall, Brandberg is a poorly sorted, unconsolidated sedimentary deposit. Three attributes indicate that Brandberg is not a lithified unit (i. e., not sedimentary rock): (1) fractures and (2) veins do not cut across the unit and its clasts, and (3) it is not emergent from beneath an overlying rock unit. The largest boulders are angular and of several meters in size. Many of the boulders and cobbles are angular, slab-shaped, and dip at steep angles relative to the surface (Fig. 4e). Supplementary Fig. 2-3 shows that Brandberg has a similar particle size distribution to Blackfoot of D50 of 13 mm, D84 of 44 mm, and D16 of 4 mm. Size analysis was performed via images acquired along the steepened exposure shown in Figs. 4e and 5a, where the maximum exposed clast size was 409 mm.

#### 3.2.3 Evidence of lithologic diversity

The presence of boulders of differing tone in HiRISE images suggested the possibility of lithologic diversity in Brandberg, years before the rover arrived (Fig. 4a). As with boulder B at Blackfoot, Mastcam images of the boulders at Brandberg show that the light tones are not the result of an intrinsic tonal property of the individual clasts, but instead are evident in cases in which a boulder has a relatively smooth (at cm- to mm-scale), skyward-facing surface coated with eolian dust.

Mastcam images show that some of the boulder- and cobble-sized clasts seen along the east margin of Brandberg exhibit fine lamination (sub-mm to mm-scale), relatively flat surfaces at cm-scale, and are covered with a thin coating of dust (Fig. 4e). In some cases, the laminae are dipping steeply relative to horizontal, indicating that they have been displaced from their original bedding orientation (Fig. 4d). One of the ChemCam targets at the edge of Brandberg was Hoba (Fig. 5a, b), which from the images could be part of the local bedrock. The RMI mosaic of Hoba (Fig. 5b) shows a fractured rock, with fractures cutting across laminae; the rock surface is rough-textured at millimeter scales, rougher than adjacent eolian sandstone surfaces. These properties are similar to those of nearby outcrops of intact Murray-formation rocks. Some of the clasts in Brandberg that are interpreted to be Murray-formation rocks also exhibit protrusive, cm-scale concretions (Fig. 5i), as do nearby Murray-formation rocks (Fig. 5j).

Brandberg also has dark-gray boulders and cobbles (Fig. 4b–e). In some cases they are cross-stratified and laminated at millimeter scales (Fig. 5h) and thus physically resemble rocks of the Stimson formation, such as those on the nearby Emerson plateau (Fig. 1) described by Banham et al. (2018). A dark-gray ChemCam target cobble, Gibeon (Fig. 5a, c) has protruding objects (grains or concretions) of very coarse sand and granule size (i.e., 1–3 mm) and exhibits no distinct bedding. A few of the cobble-sized stones observed at Brandberg contain pebble-sized clasts or concretions and are either conglomerates, pebbly sand-stones, or sandstones containing pebble-sized concretions (Fig. 5g). Further, some of the loose, pebble-sized clasts in the Brandberg deposit resemble concretions that eroded out of Stimson-formation sandstones on the nearby (~340 m to the southwest) Naukluft Plateau (Fig. 5d–f).

### 3.3 Bimbe image observations

#### 3.3.1 Field site and stratigraphic position

**Fig. 6:**
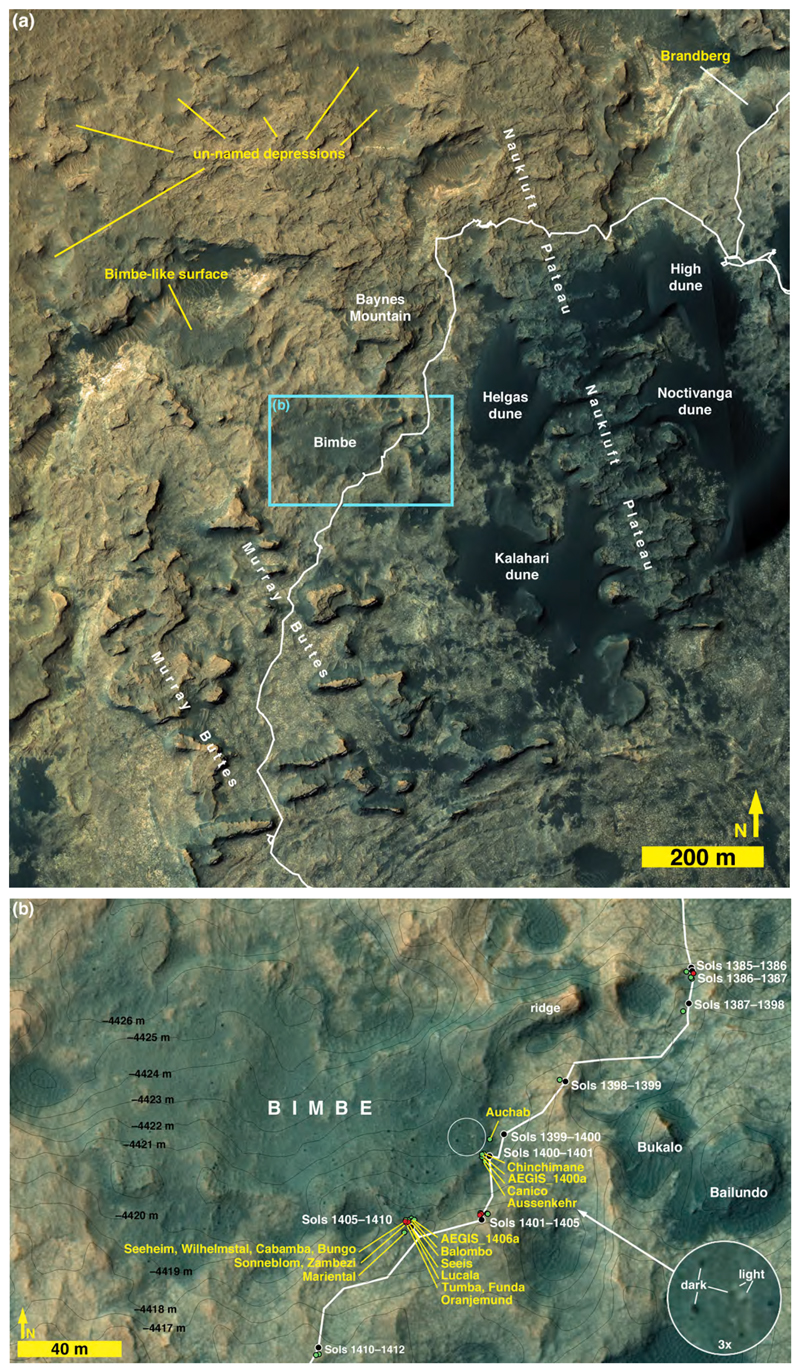
(a) Local context of Bimbe on the lower slopes of Mt. Sharp, between the Murray buttes to the south, Naukluft Plateau and some of the Bagnold dunes to the east, and Baynes Mountain to the north. The white trace is the Curiosity rover traverse; the drive direction was from northeast (upper right) toward the south (bottom left of center). Blue box indicates the location of (b): Bimbe with ChemCam, APXS, and MAHLI targets indicated in yellow. Green dots indicate ChemCam targets, red dots indicate APXS targets, and black dots indicate rover parking spots. The inset, a 3× expanded view of the area inside the white circle next to target Auchab, shows an example of light- and dark-toned boulders as observed from the orbiting HiRISE camera. The white trace is the Curiosity rover traverse; the drive direction was from the northeast (upper right) toward the south (bottom center). The elevation contours are derived from HiRISE stereo-pair products calibrated to Mars Global Surveyor (MGS) Mars Orbiter Laser Altimeter (MOLA), topography by Parker and Calef (2016). (For interpretation of the references to color in this figure, the reader is referred to the web version of this article.)

Located due north of the Murray buttes, Bimbe was the most investigated of the three heterolithic units visited by the Curiosity rover. As viewed in HiRISE images (Fig. 6), Bimbe is a boulder-bearing surface with an irregular shape that covers ~16,800 m^2^. It is intermediate in tone relative to the nearby darker-toned eolian sands of the Bagnold dune field (e.g., Bridges et al., 2017) and the surrounding lighter-toned, dust-coated outcroppings of the Murray formation. Most of the boulders visible in HiRISE images of Bimbe are dark toned but a few, as in Brandberg and Blackfoot, exhibit a light tone (Fig. 6b inset).

**Fig. 7:**
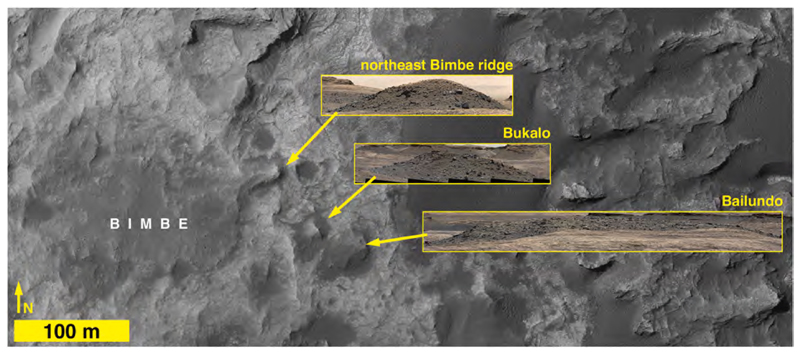
A ridge on the northeast side of Bimbe, which trends northeast-southwest (azimuth ~65.5°), is mantled with cobbles and small boulders in Mastcam images (inset). The ridge resembles nearby boulder- and cobble-mantled hills named Bukalo and Bailundo (insets). These are likely examples of buttes that have weathered to the point that they are hills with remnants of the capping rock taking the form of small boulders and cobbles (cf. Migon et al., 2018).

The easternmost extent of Bimbe is a ~37 × 11 m ridge that protrudes northeastward and is mantled with small boulders and cobbles (Fig. 7). This ridge exhibits an eastward azimuth of ~65.5°, which is within the range of the azimuths (50°–95°) exhibited by ridge forms in the nearby Murray buttes, Naukluft Plateau, and Baynes Mountain areas (Fig. 6). Approximately 65–90 m southeast of Bimbe are two hills mantled with dark-gray cobbles and small boulders, named Bukalo and Bailundo (Fig. 7); Mastcam image mosaics show that these resemble the northeast-projecting ridge on the east side of Bimbe (Fig. 7).

**Fig. 8:**
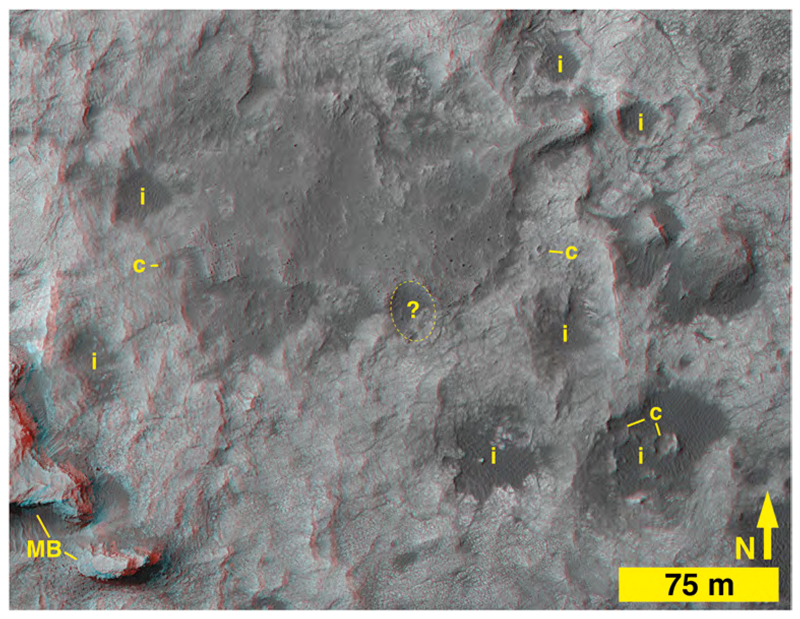
Stereo pair anaglyph of Bimbe (large, dark-toned patch) and its vicinity. Most of the boulders observed at Bimbe occur on the elevated portions of the south margin of Bimbe. The feature within the dashed ellipse, indicated with a “?,” might be the eroded remains of an impact structure. Definitive impact craters are labeled with “c” and probable impact structures are labeled with “i”. Dark-toned eolian sand, expressed as bedforms, has collected in the impact structure depressions. Features labeled “MB” are a couple of the Murray buttes. (Anaglyph will only appear in 3D on the web version of this article.)

With the exception of the northeastward-projecting ridge, the rest of Bimbe generally slopes down toward the north and lies in a broad depression in the Murray formation which is bordered in this area by Stimson formation-capped buttes and mesas on the lower northward-facing slope of Mt. Sharp (Figs. 6b and 8, Supplementary Fig. 1-3). The average slope from Bimbe to a Bimbe-like surface to the north (Fig. 6a) is about 4.5%. Bimbe occurs between the elevations −4417 and − 4426 m and exhibits 9–10 m of surface relief, more than at Blackfoot or Brandberg. A higher proportion of boulders, visible in HiRISE images, occurs on the southern, higher-elevation side of the unit (Figs. 6, 8). Several somewhat circular and elliptical depressions near and adjacent to Bimbe exhibit dark-toned bedforms within them; by analogy to similar features throughout Gale crater, these are probably meteoritic impact structures that have trapped eolian sand. One such elliptical depression occurs on the southeast side of Bimbe and some of the larger boulders encountered during the rover investigation of Bimbe occur there (dashed ellipse in Fig. 8).

The Curiosity rover was driven along the east and southeast margins of Bimbe and stopped at four locations (Fig. 6b). The first site was visited on sols 1399–1400 (12–13 July 2016) and the second on sols 1400 and 1401 (13–14 July 2016). The rover was parked at a stand-off position some meters from the edge of Bimbe, to image the boulders and cobbles during sols 1401–1405 (15–19 July 2016), where ChemCam observed Murray bedrock. Then it was driven to the last investigation site for study of Bimbe over sols 1405–1410 (19–24 July 2016). At the first two Bimbe investigation sites (sols 1399–1400 and 1400–1401), only remote-sensing observations (ChemCam, Mastcam, Navcam, Hazcam) were acquired; at the final site, MAHLI and APXS were also deployed. While a large number of boulders and cobbles were observed by Mastcam at Bimbe, the descriptions given here focus on the ones that were also observed by ChemCam, MAHLI, and APXS.

**Fig. 9:**
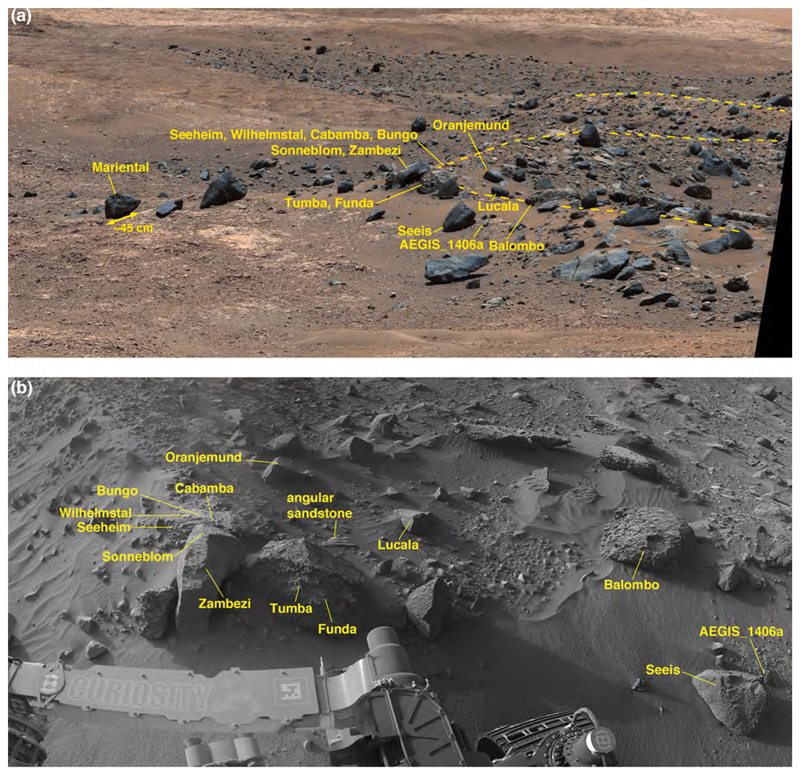
a) Work site for sols 1405–1410, viewed from the east. Names of targets investigated using ChemCam, Mastcam, APXS, and MAHLI are indicated. The dashed trace approximately indicates the crest of a low ridge line that defines the southern margins of Bimbe. (b) Navcam view of the work site. ChemCam, APXS, and MAHLI targets are indicated (except Mariental, which is to the left of this scene). The stone labeled “angular sandstone,” imaged by MAHLI on Sol 1407, is also shown in Fig. 15 and was not a named target. For scale, the width of the rover wheel (right of lower center) is 40 cm.

ChemCam observed two targets on Sol 1400 (Fig. 6b), Auchab and AEGIS_post_1400a (the latter selected via rover autonomous targeting software; Francis et al., 2017). As the latter target was observed after a rover drive, it is at the same site as the Sol 1401 ChemCam targets, Aussenkehr, Canico, and Chinchimane (Fig. 6b). The final Bimbe site visited by Curiosity (sols 1405–1410) displayed boulders and cobbles of various morphologies along a low (≤1 m) ridge trending to the northwest (Fig. 9a). Several boulders were south of the ridge, sitting on Murray-formation bedrock just past the southern margin of Bimbe. The boulders and cobbles nearest to the rover were examined using ChemCam, Mastcam, MAHLI, and APXS (Fig. 9b). These included two boulders in the robotic arm workspace, one a conglomerate with targets Tumba (MAHLI only) and Funda (APXS raster and MAHLI), the other a dark-gray apparent sandstone with targets named Sonneblom (APXS, MAHLI, and ChemCam) and Zambezi (MAHLI and APXS). Just behind those boulders lay a partially exposed conglomeratic boulder containing ChemCam targets Seeheim, Wilhelmstal, Cabamba, and Bungo. About half a meter farther on the low ridge to the right (north and east) of the rover were ChemCam targets Oranjemund and Lucala (Fig. 9b). Farther to the right were several conglomeratic stones, including a rounded one containing ChemCam target Balombo, along with a dark-gray fine boulder named Seeis and another target selected by the rover, AEGIS_-post_1406a. Finally, to the left of the rover was a ChemCam target named Mariental (Fig. 9a); this small boulder was not on the surface of Bimbe proper, but instead sat isolated on Murray-formation bedrock to the north of the low ridge which forms the southern margin of Bimbe.

#### 3.3.2 Physical sedimentology of the Bimbe deposit

The first two investigation sites (sols 1399–1400, and sols 1400 and 1401; Fig. 6b) were relatively flat with the edge of the unit in contact with Murray-formation bedrock and observed to be relatively abrupt. Some eolian sand and regolith occurs at the outer edge, and then, moving inward—amid a regolith of granules, pebbles, and eolian sand—small cobbles, then larger cobbles and small boulders occur a bit farther in from the edge. The final investigation site (sols 1405–1410; Fig. 6b) was a ridge, oriented approximately west-southwest to east-northeast, littered with larger boulders and cobbles. In all cases, some of the cobbles and boulders sit on the surface and others protrude from the regolithic subsurface. The boulders and cobbles exhibit an array of orientations relative to internal bedding structure (where present) and exhibit a range of shapes from angular to sub-rounded. Patches of eolian sand form ridges in the lee of obstacles.

HiRISE images show Bimbe to be in contact with Murray-formation exposures all around its perimeter. For the east and south side of Bimbe, imaged using the rover Mastcams, Navcams, and Hazcams, the same relationship is observed from the ground—Bimbe overlies Murray-formation rock; nowhere does it contact Stimson-formation rocks or other rock units. In addition, no rock unit overlies Bimbe; it does not appear to be emergent from beneath a rock unit. Bimbe is not cross-cut by fractures or veins or intrusive rock. Like Brandberg and Blackfoot, Bimbe is not a rock unit but is, instead, an unconsolidated accumulation of poorly sorted sediment. Grain-size analysis of the Bimbe unit produced a D50 of 9 mm, D84 of 33 mm and a D16 of 4 mm (Supplementary Fig. 2-3).

#### 3.3.3 Evidence of lithologic diversity

As noted, Bimbe was suspected of being heterolithic because HiRISE images show that it includes light and dark-toned boulders. Like Brandberg and Blackfoot, the boulders that exhibit light tone in HiRISE images are actually dark gray and present a relatively flat, dust-coated surface toward the sky. Mastcam images and analyses made using ChemCam, APXS, and MAHLI, do, of course, show that Bimbe contains a range of lithologies. These include boulders and cobbles that are conglomerates, sandstones, and reddish stones interpretable as Murray-formation rocks (mudstones or very fine sandstones).

##### 3.3.3.1 Sandstone boulders and cobbles

Intact outcrops of sandstone encountered along the Curiosity rover traverse, whether deposited in eolian, fluvial, deltaic, or near-shore lacustrine settings, have generally been dark gray in color (e.g., Grotzinger et al., 2013; Grotzinger et al., 2015; Anderson et al., 2015; Treiman et al., 2016; Edgar et al., 2017; Banham et al., 2018). Some of these sandstones exhibit clear and obvious bedding, others are more “massively” bedded at centimeter to meter scale. Some exhibit protrusive (erosion-resistant) grains of very coarse sand, granules or pebbles; others exhibit protrusive concretions that, in places, can give the rock surface a “knobby” appearance at centimeter scales. Some of the sandstones encountered by the rover also have pits in their surfaces, perhaps sites at which a coarse clast or concretion was liberated by weathering. All of these properties of the sandstones seen throughout the MSL mission in Gale crater are also found in one or another boulder or cobble at Bimbe. As a matter of communication convenience, we describe the dark-gray sandstone boulders and cobbles at Bimbe that display some form of stratification or bedding as layered, those which do not exhibit layers are described here as massive, and those with numerous mm- to cm-scale protrusions are here described as nodular. One modification to this scheme is that some dark-gray sandstones have similar appearance including apparent grain size range, and identical chemistry to the layered targets. We will show later that this class (both in layered and not apparently layered form) has been observed earlier along the traverse, and so we group these targets together here as well, calling them all “layered.”

###### 3.3.3.1.1 Massive boulders and cobbles

**Fig. 10:**
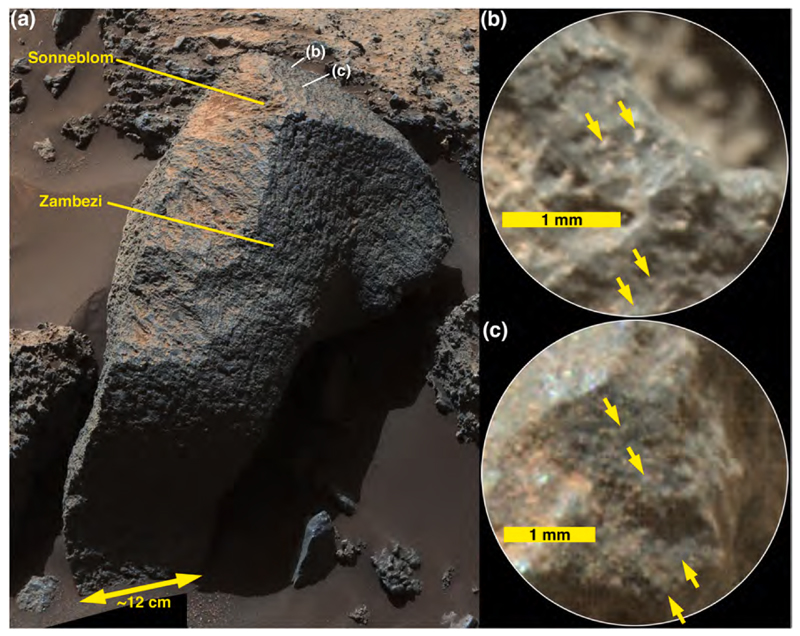
(a) Boulder containing targets Sonneblom (ChemCam, APXS, MAHLI) and Zambezi (APXS, MAHLI). The boulder is a dark-gray, pitted sandstone; the locations of the MAHLI views in (b) and (c) are indicated. (b and c) Close-up views of the surface; the arrows point to several individual grains.

**Fig. 11:**
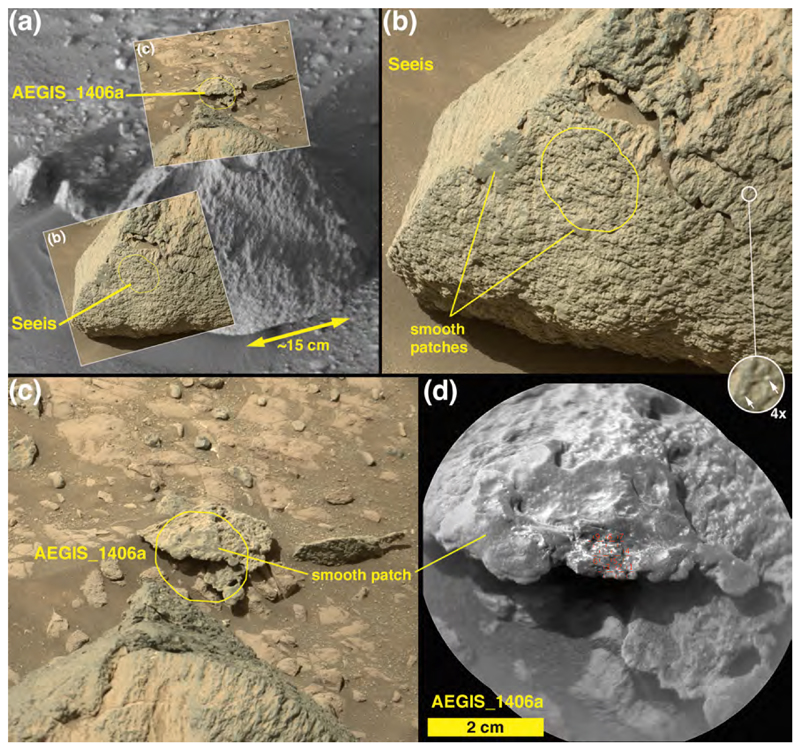
(a) Context for two ChemCam targets, Seeis and AEGIS_1406a, on two separate stones at Bimbe. (b) Seeis target; outline indicates location of ChemCam RMI coverage, 4× inset indicates examples of the largest (mm-scale) grains. (c) AEGIS_1406a target; outline indicates the location of the ChemCam RMI coverage shown to the right. (d) Close-up view, with crosshairs indicating LIBS observation points on AEGIS_1406a.

A “massive” sandstone, for this discussion, is one that exhibits little or no evidence of layering within the cobble or boulder at the scales observable by MAHLI, RMI, and the Mastcams, Navcams, and Hazcams; this does not mean that the original outcrop from which the boulder or cobble eroded was not layered at some greater (decimeter to meter) scale. The boulder on which the targets Sonneblom and Zambezi occur provides an example (Fig. 10). It is dark gray, sand grains are observable (Fig. 10b, c), and the rock surface is pitted at millimeter to sub-millimeter scales, which appears to be a characteristic of this group. Mastcam-100 and ChemCam target, Seeis (Fig. 11a, b), is another example. Like Sonneblom, it is dark gray and pitted; dust-free surfaces can glint in sunlight and some well-rounded mm-scale grains protrude from the rock surface (e.g., 4× inset in Fig. 11b). Seeis also seems indistinctly layered. Some of these massive apparent sandstone boulders and cobbles also exhibit patches of relatively smooth, dark-gray material (Fig. 11); it is unclear whether these are intrinsic to the sediment or the remains of mineralization formed on fracture walls in the original, intact strata. One such patch was observed by ChemCam on target AEGIS 1406a (Fig. 11c, d).

**Fig. 12:**
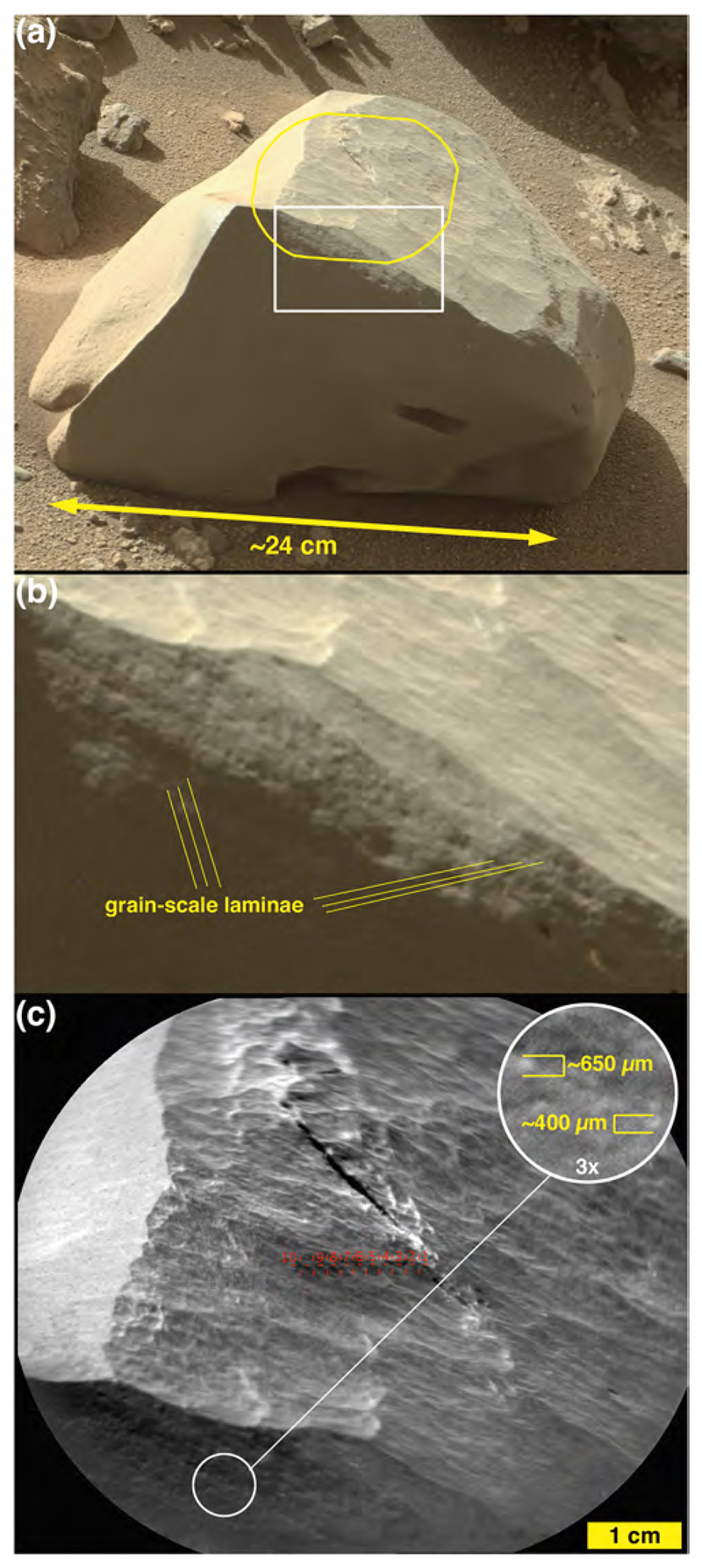
(a) Dark-gray sandstone cobble target named Oranjemund. White box indicates location of expanded view in (b); outline indicates ChemCam RMI coverage in (c). (b) View showing grain-scale bedding, which runs diagonally from upper left to lower right. (c) Close-up view of Oranjemund; inset shows 3× expanded view with measurements on features that could be among the larger sand grains in the rock (i.e., most of the grains are smaller than ~400 μm). Oranjemund is compositionally identical to layered target Chinchimane (Fig. 14).

Another cobble examined using Mastcam-100 and ChemCam, called Oranjemund (Fig. 12), is inferred to be a sandstone because it is dark gray and resistant to erosion; only some of its larger grains are perhaps resolvable in the RMI and Mastcam-100 images acquired are 400–700 μm in size. The majority of grains are likely smaller than this. The sunlit portion of the stone facing the Mastcam-100 in Fig. 12 shows that it is laminated at the grain scale (a lamina is the smallest megascopic layer that can be observed in a sedimentary rock; Campbell, 1967). Oranje-mund exhibits sharp corners in some places, especially near the top, and more rounded edges near the base. Some glinting of the surface near the apex is evident in the Mastcam image, and the surface investigated using ChemCam is scalloped or fluted. We will show in Section 3.4.1 that Oranjemund is chemically related to the more obviously layered cobble, Chinchimane, described next.

###### 3.3.3.1.2 Layered boulders and cobbles

**Fig. 13:**
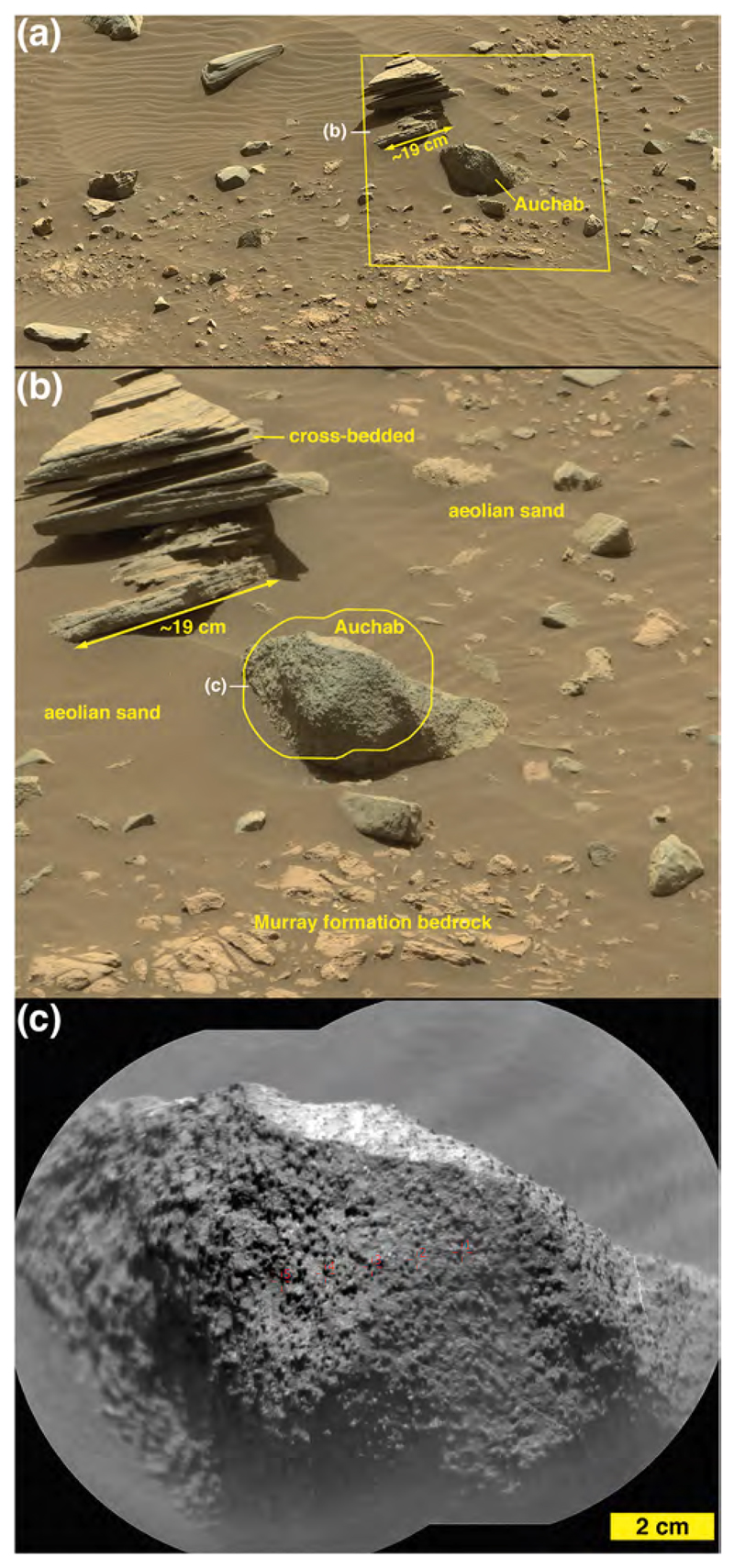
(a) Local context for ChemCam target Auchab; yellow outline indicates location of panel (b): closer view of knobby gray target Auchab; yellow outline indicates ChemCam RMI coverage in (c). Note the nearby angular, cross-bedded sandstone clast. Both stones are surrounded by windblown sand and overlie reddish Murray-formation bedrock. (c) ChemCam RMI mosaic showing the nodular texture of Auchab. (For interpretation of the references to color in this figure, the reader is referred to the web version of this article.)

**Fig. 14:**
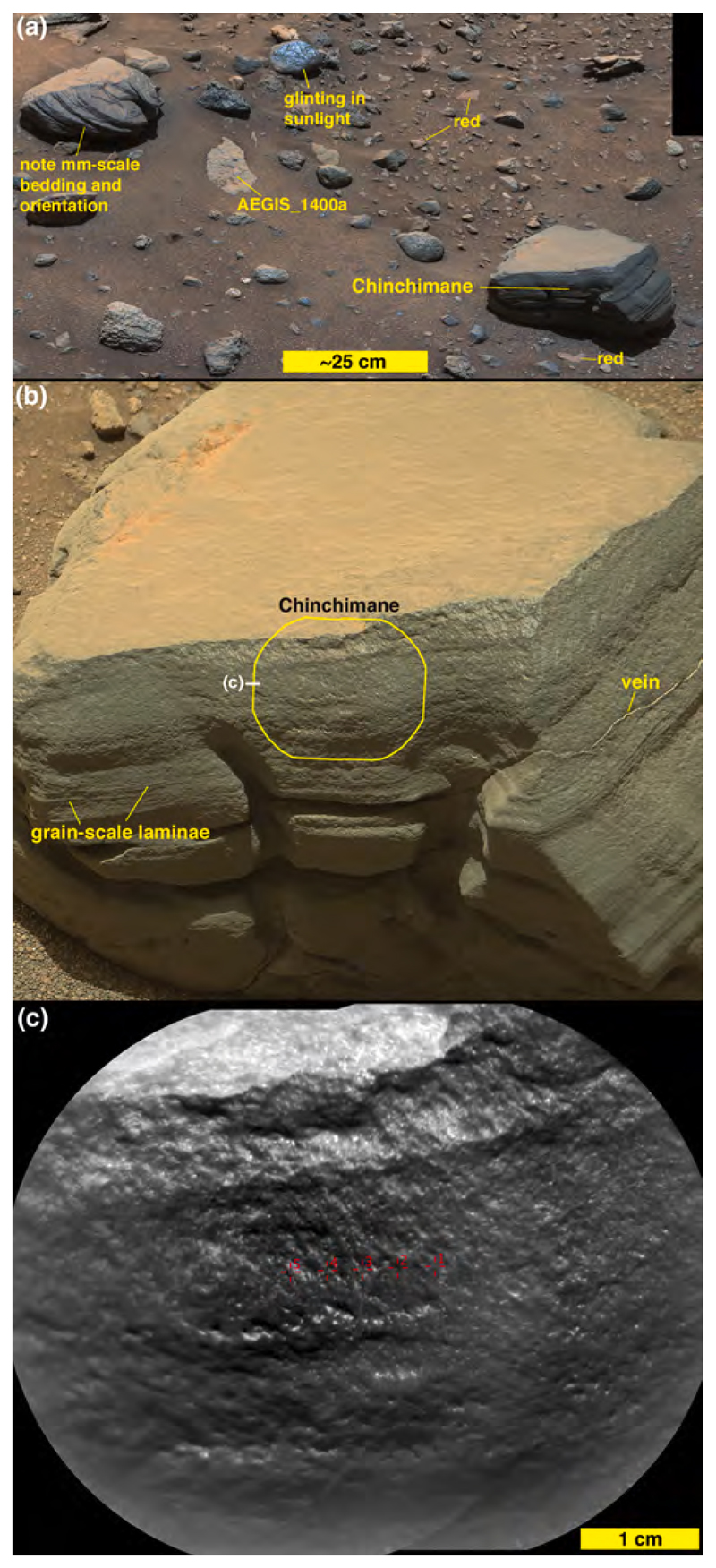
(a) Local context for layered gray sandstone cobble target Chinchimane. Note the differences in cobble orientation relative to their bedding structure, as well as the diversity of cobble colors, response to sunlight, and sedimentary structure and texture. (b) Mastcam-100 view. Bedding is approximately parallel and thin (sand grain scale thickness). A fracture filled with a while material (vein) cuts across bedding. The yellow outline indicates the location of the ChemCam RMI coverage shown in (c), where a few individual sand grains can be identified in a well cemented and well sorted sandstone. (For interpretation of the references to color in this figure, the reader is referred to the web version of this article.)

 Mastcam images show that some boulders and cobbles at Bimbe are not only layered, they exhibit cross-bedding at scales to tens of centimeters (Fig. 13a, b). Still others are finely layered, in parallel beds, at sub-millimeter scales. The ChemCam and Mastcam-100 target, Chinchimane, provides an example (Fig. 14). Classified as a coarse cobble, the overall size of Chinchimane is roughly 15 × 25 × 12 cm. Its top is flat and parallel to the bedding, suggesting breakage along a bedding plane. Grains in Chinchimane are challenging to identify in Mastcam-100 and ChemCam RMI images; this can be an indicator that grains are smaller than the spatial resolution of the images, or it can be an indicator that pores are filled with cement and the cement is difficult to distinguish from the grains, or a bit of both. A few rounded objects visible in the RMI images of Chinchimane are in the coarse to very coarse sand range, roughly 700–1100 μm in size; these might be sand grains and might indicate that the rock includes coarse to very coarse sands. Pitting at the scale of ~500 μm to several mm can be also seen where the layering is indistinct; the scale of these pits is consistent with the tentative identification of grains. A light-toned fracture or vein cross cuts some of the layers along the right side of the Mastcam-100 image.

**Fig. 15:**
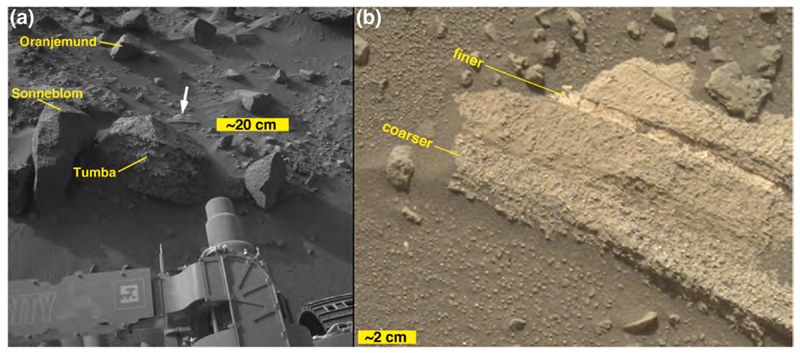
Angular sandstone cobble with fine-grained interbeds. (a) Location (white arrow) of the cobble relative to the larger boulders in the rover workspace during sols 1405–1410. (b) Highest spatial resolution view of the cobble. Coarser grains are ~700 μm; the skyward rock face cuts steeply across the original bedding. A recessed interval of ~1 cm thickness consists of grains too small to resolve.

One layered sandstone cobble, explored via MAHLI from a distance of ~1 m, is an angular slab (“angular sandstone” in Fig. 9b) oriented such that the bedding points approximately skyward (Fig. 15); its ~700 μm grain-scale-thickness bedding includes a much finer-grained, light-toned, ~1 cm-thick recessive stratum. Nothing quite like the stone in Fig. 15 has been observed in an intact outcrop during the rover’s explorations in Gale to date.

3.3.3.1.3

**Fig. 16:**
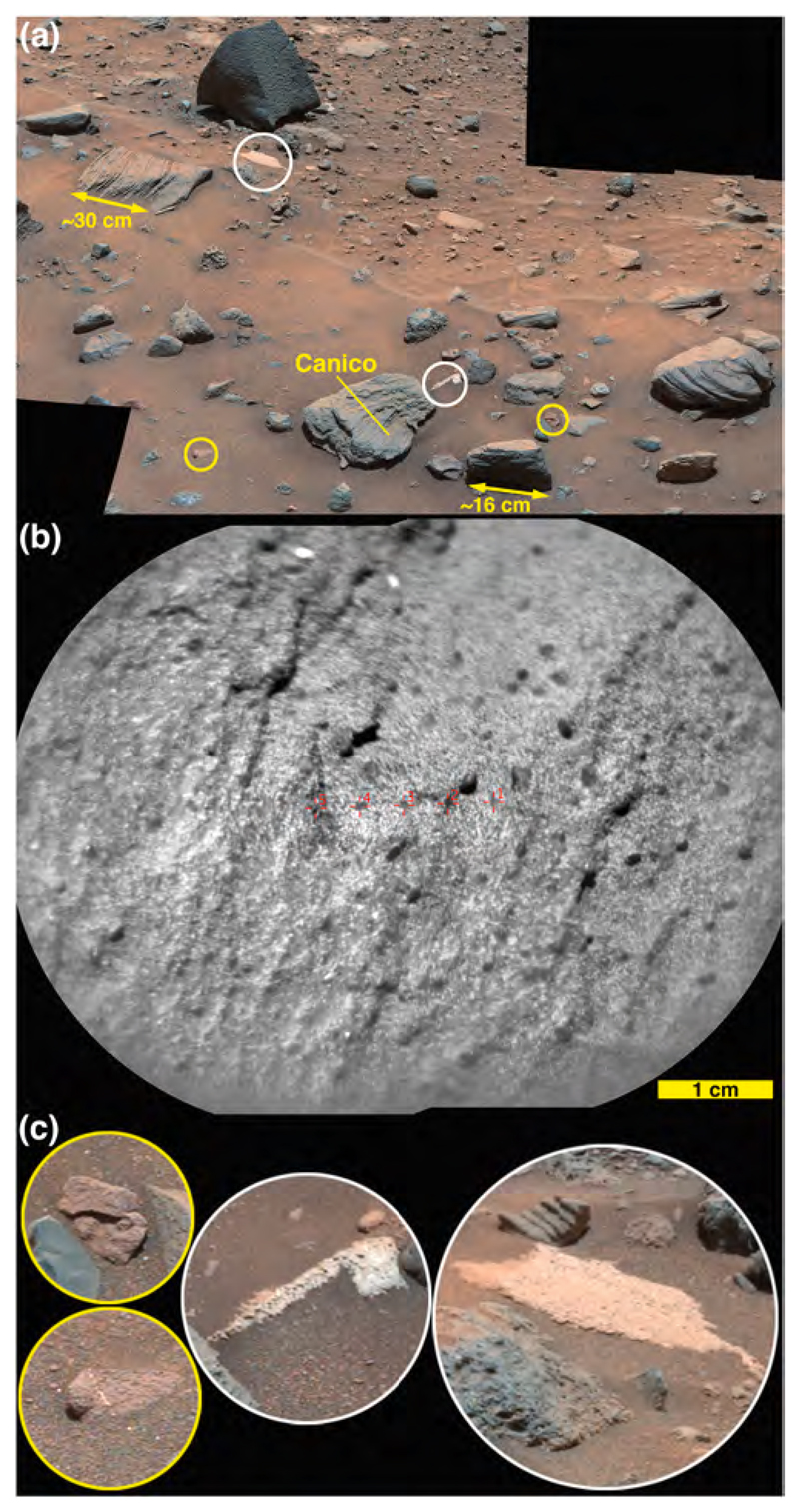
(a) Example showing diversity of cobble and boulder orientations, sedimentary structures, and colors at Bimbe. The white circles indicate examples of white stones shown in (c); the yellow circles indicate examples of “red” stones in (c). (b) Knobby sandstone target, Canico. (c) Examples of “red” (left) and white (center and right) cobbles which are minor constituents of the Bimbe deposit; locations and relative sizes are shown in (a). The red stone at the lower left in (c) has a white vein cutting across bedding within the stone. (For interpretation of the references to color in this figure, the reader is referred to the web version of this article.)

*Nodular sandstone boulders and cobbles.* Some of the sandstone boulders and cobbles at Bimbe display surfaces of many strongly protrusive sand-sized grains, concretions, or both. Some of them are light gray or white; most are darker shades of gray. Two ChemCam targets, Auchab and Canico, provide good examples. At millimeter to centimeter scale, they both have rough textures with protrusive objects of approximately 500–2000 μm size (Figs. 13b, c and 16a, b). Of the two, Auchab contains the highest density of these objects, which appear to cover nearly 50% of its surface.

##### 3.3.3.2 Conglomerate boulders and cobbles

**Fig. 17:**
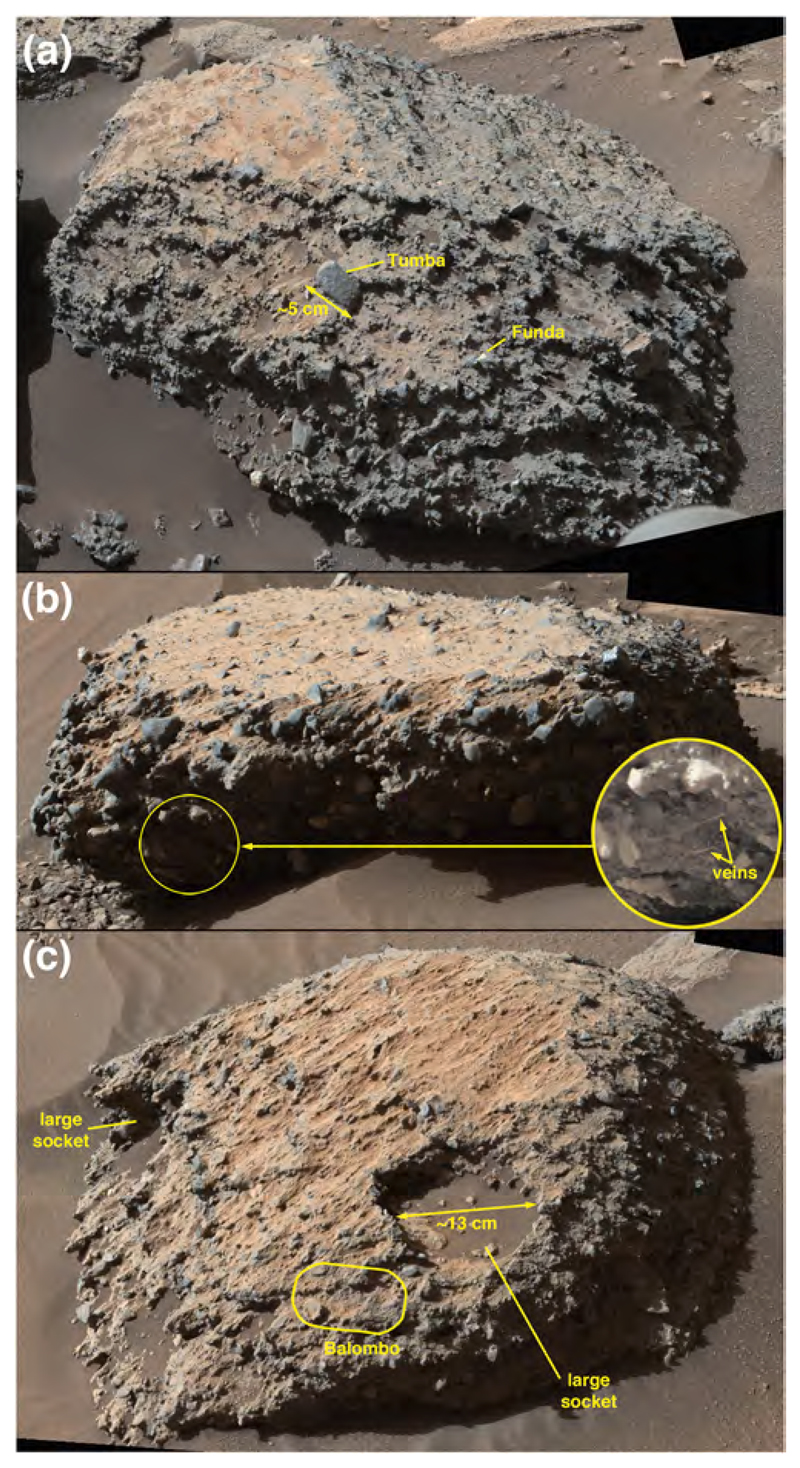
(a) Conglomeratic boulder containing MAHLI targets Tumba and Funda; the latter was also an APXS target. (b) Conglomeratic boulder, ~60 cm wide, that includes veins that cut across the sandy matrix (inset). (c) Conglomeratic boulder that includes the ChemCam target Balombo (outline indicates RMI image coverage).

Some of the boulders and cobbles at Bimbe are conglomerates; that is, they are boulder-sized and cobble-sized fragments of conglomeratic bedrock. A conglomerate is a clastic sedimentary rock that consists of >10% clasts coarser than sand size. Conglomerates are distinguished from breccias because their coarse clasts are mostly sub-rounded to rounded, whereas breccia clasts are generally angular to sub-angular. The larger examples of conglomerates examined via Curiosity at Bimbe are associated with the low ridge that marks the southern boundary of Bimbe. Generally, they are gray and contain gray pebble clasts in a gray sandy matrix (Fig. 17). The pebble clasts have a range of shapes, from angular to sub-rounded and lower to higher sphericities. Some of the boulders are weakly stratified (Fig. 17a), supporting the interpretation that they are fluvial conglomerates. A few boulders contain cross-cutting veins (Fig. 17b), indicative of fracturing and fluid flow at a time when the conglomerates were still part of an intact rock unit or units at some depth below the Martian surface. Some of these conglomerate stones lie on the surface; others protrude from the regolith. In some cases, a litter of pebbles, liberated from the conglomeratic stones, lies on the ground in the immediate vicinity (e.g., lower left of Fig. 17b). Attributes of four specific conglomerate boulders at the sols 1405–1410 field site were examined in some detail using ChemCam (three boulders) plus MAHLI and APXS (one boulder).

**Fig. 18:**
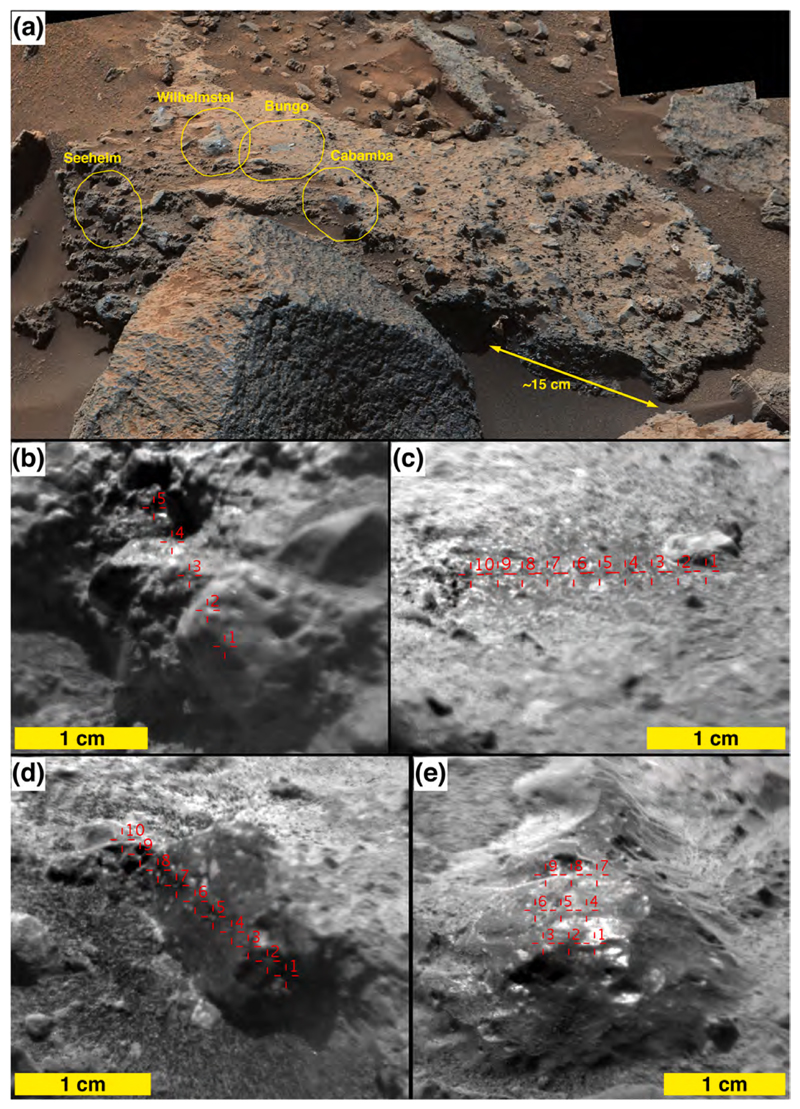
(a) Conglomeratic boulder investigated via four ChemCam targets. (b–e) Respective RMI views and LIBS target areas of Seeheim, Bungo, Cabamba, and Wilhelmstal. Note the vitreous luster of Wilhelmstal, and similarity to Cabamba.

One of the conglomerate boulders at the sols 1405–1410 location was the site of four ChemCam targets, Bungo, Seeheim, Wilhelmstal, and Cabamba (Fig. 18). The leftmost target, Seeheim, exhibits pebble clasts that protrude somewhat more than in other areas of the boulder surface, perhaps a result of greater eolian weathering. ChemCam LIBS observation points 1 and 2 at Seeheim hit a specific clast, while the remaining points hit other materials—two hit individual clasts, two hit matrix material (Fig. 18b). In the same boulder, the target named Cabamba is a single, angular clast that contains some light-toned, angular sub-clasts or mineral grains (Fig. 18d). Wilhelmstal is a rounded clast that might also have a few light-toned sub-clasts or mineral grains within it (Fig. 18e). After the laser pulses removed dust from Wilhelmstal, it presented a glinting, vitreous luster. The Wilhelmstal pebble also exhibits small, rounded indentations that could be vesicles. We will show in Section 3.4 that Cabamba and Wilhelmstal have nearly identical, homogeneous compositions. The fourth target on this conglomeratic boulder, Bungo, consists entirely of sandy matrix, with grains < 500 μm in size (Fig. 18c).

A second conglomerate boulder investigated using ChemCam had a singular target, Balombo (Fig. 17c). The boulder has a relatively rounded shape, overall, with mostly angular pebbles protruding from its surface. At least two large sockets are present; these might once have held larger clasts. The ChemCam LIBS observation points on Balombo appear to have hit sandy matrix and a few larger (<2 mm) grains.

**Fig. 19:**
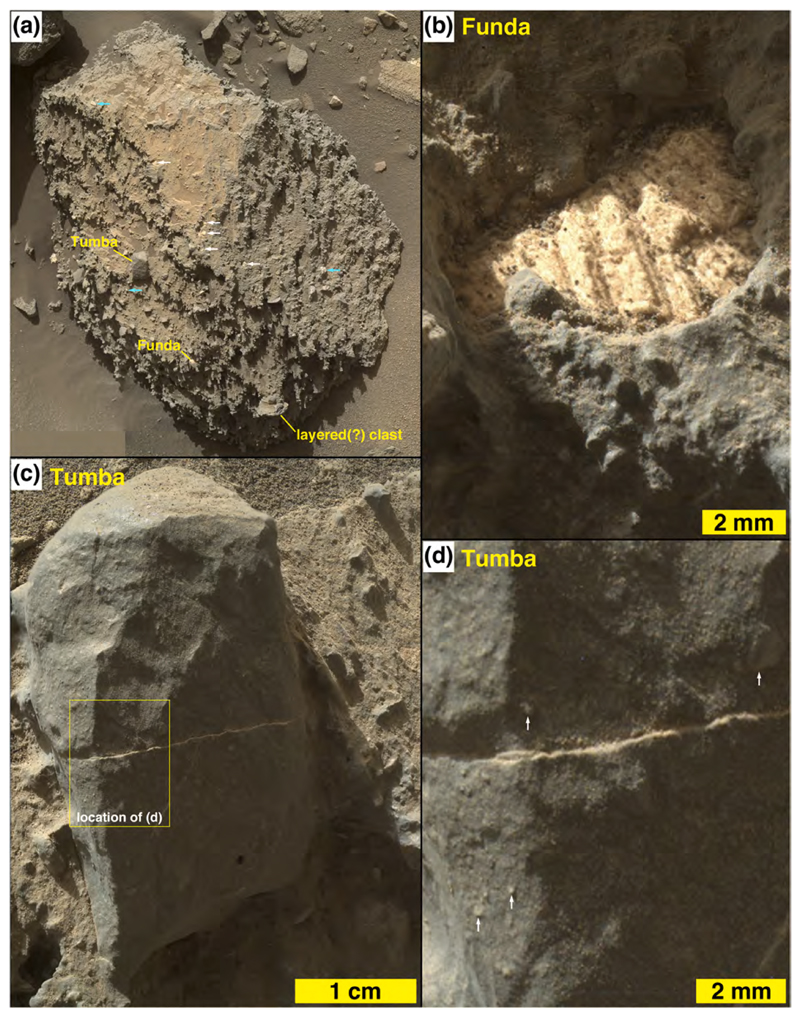
(a) Conglomeratic boulder that includes the APXS/MAHLI target, Funda, and the MAHLI target, Tumba. White arrows indicate recessed white objects similar to Funda; blue arrows indicate light-gray and white protrusive pebble clasts. (b) Close-up view of white, recessed, banded object (clast or void fill) comprising the target Funda. (c) Pebble clast target named Tumba. (d) Close-up view of a portion of the Tumba pebble clast, showing that it is a sandstone; arrows point to a few example grains. (For interpretation of the references to color in this figure, the reader is referred to the web version of this article.)

A fine boulder in the rover’s robotic arm workspace (sols 1405–1410), measuring ~50 cm × ~45 cm × ~30 cm, was investigated using the MAHLI and APXS (Figs. 17a and 19). Like the others at Bimbe, it contains mostly gray pebbles of various shapes and sizes set into a gray sandy matrix. These sediments are weakly stratified. It also has recessed features that—where dust cover is minimal—are white. These white objects are of sizes similar to the pebbles and granules in the boulder. MAHLI and APXS were deployed to investigate one of them at a target named Funda. The white feature at Funda is banded (Fig. 19b). The other target investigated in this boulder, using MAHLI and Mastcam, only, is a pebble-sized sandstone fragment (Fig. 19c). This target, Tumba, provides definitive evidence for recycling of sedimentary rock on Mars (Edgett et al., 2018).

**Fig. 20:**
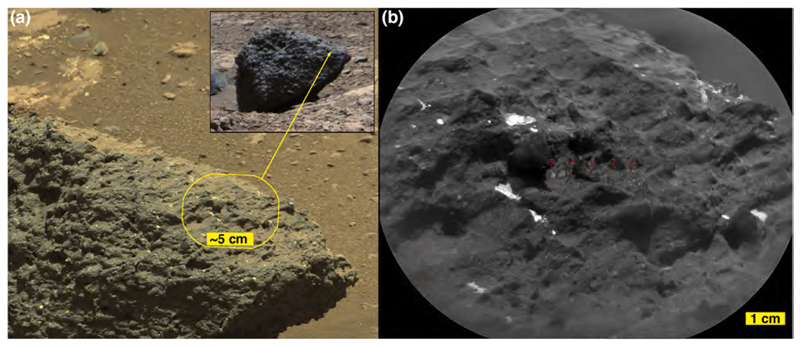
(a) Conglomeratic boulder with ChemCam target, Mariental. Inset shows the entire boulder. (b) Close-up view of Mariental and the locations of ChemCam LIBS observations. Recessive white features are interpreted to be similar to Funda (Fig. 19).

Mariental (Fig. 20) is distinct from the other conglomeratic boulders because it occurs separate from the Bimbe deposit, an outlier about ~3 m to the immediate north (Fig. 9a). Perhaps this boulder rolled or slid to its present location or it was left behind by a retreating margin of the deposit. The ground under Mariental is not littered with pebbles (Fig. 20a inset). As in some of the other conglomerate boulders at Bimbe, the coarser clasts of Mariental are set in a dark, gray, sandy matrix and some of its gray pebble clasts present a glinting, vitreous luster. In addition, and like the boulder that contains the targets Tumba and Funda, Mariental includes recessed, pebble-sized white features (Fig. 20b) that could be either clasts or void-filling minerals similar to Funda (Fig. 19b).

#### 3.3.3.3 Murray-formation pebbles and cobbles, and white stones

Reddish and white stones are present but are the least common of the varieties observed in Mastcam mosaics of eastern Bimbe. Fig. 16a, c shows a couple examples of the reddish stones; none were investigated using the ChemCam, APXS, or MAHLI. They are inferred to be lithic fragments of Murray-formation rocks. Overall, the Murray formation consists largely of mudstones and very fine sand sandstones (e.g., Grotzinger et al., 2015; Rampe et al., 2017; Rivera-Hernández et al., 2019) with colors ranging from various shades of light gray to a purplish gray to brick red. Locally, near and up-slope from Bimbe, much of the Murray-formation bedrock is of the brick-red variety, like the stones observed in the Bimbe deposit.

Angular white or very light gray pebbles are also observed in the deposit at Bimbe. Some of them are angular and relatively flat (Fig. 16a, c). Experience gleaned from studies of the Stimson formation showed that white “halos” of altered rock surround some fractures in the otherwise dark-gray sandstone (Frydenvang et al., 2017; Yen et al., 2017; Banham et al., 2018). Some of the white or very light gray pebbles and cobbles, like those in Fig. 16c, could be fragments of such altered Stimson-formation sandstones. Some of the smaller white pebbles could be fragments of vein material. Essentially all of the white vein materials observed thus far throughout the Curiosity traverse have been calcium sulfate (Nachon et al., 2014, 2017).

### 3.4 Bimbe compositions

Compositions are presented here, starting with Bimbe, as it is the only one of the three sites that had enough targets to classify them into groups which can be readily compared with other rocks examined during the mission. As described in Section 3.5, targets at Blackfoot and Brandberg have Murray and Stimson-formation compositions.

ChemCam performed observations at 125 locations on sixteen Bimbe targets; APXS observed six locations (3 separate targets, one as a raster) on two distinct blocks. The compositions for each ChemCam observation point are given in the Supplementary material. The mean abundances of the major elements for each ChemCam target are given in Table 1. Standard deviations from the mean of the ChemCam observation points within each target (rasters of 5, 9, or 10 points) are given in Table 2. The distance to the target generally does not appear to play a role in the standard deviations, as the most distant target, Auchab, at 5.2 m, has relatively low standard deviations compared to closer targets. Comparison with standard deviations taken from the single-shot spectra (n = 25, dust-free) within a given observation point, presented in the Supplementary material, shows that the variations between points are nearly always larger than the variations between laser shots within an individual point, as would be expected for targets that are heterogeneous on a scale the size of the laser beam (350–550 μm; Maurice et al., 2012) or larger.

Table 3 presents abundances of several trace elements to which ChemCam is sensitive. These are target averages, similar to Tables 1 and 2. In a number of cases for Rb and Sr, one or more points fell below the limits of quantification. In these cases, the mean of the remaining points is given with a “<” sign to indicate the reduction caused by the point with no detection. Standard deviations between observation points of a given target tend to reflect trends in standard deviations of the major elements for the different targets and groups of targets, indicating that, similar to the major elements, the measurement precision is much better than the accuracy, and providing information on the heterogeneity of the targets at the size scale of the laser beam.

Table 4 provides abundances and precisions for APXS observations of Bimbe targets Sonneblom, Zambezi, and Funda.

**Fig. 21:**
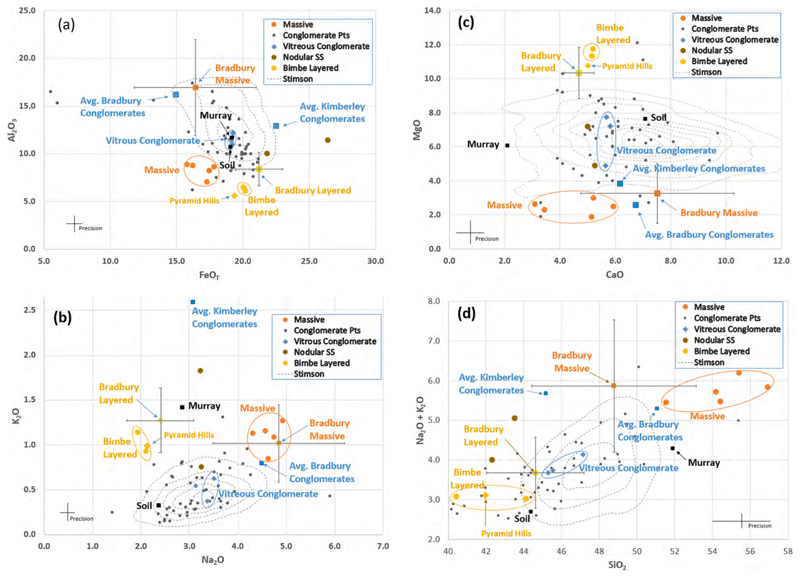
Major-element abundances, in wt%, of ChemCam targets at Bimbe. Larger symbols indicate averages of individual targets for Aussenkehr, Lucala, Seeis, Sonneblom, AEGIS_post_1406a (“Massive,” red dots); Wilhelmstal, Cabamba (“Vitreous Conglomerate,” blue diamonds, also includes AEGIS_post_1400a); Chinchimane, Oranjemund (“Bimbe Layered,” yellow dots), Auchab and Canico (“Nodular SS,” brown dots). Smaller symbols represent individual observation points for the conglomerates (“Conglomerate Pts”), which have more diverse compositions. Circles indicate groupings of Bimbe layered (yellow), massive (red), and vitreous conglomerate (blue) targets. Standard deviations between individual LIBS observation points within a target are given in Table 2. Upper limits on precision for individual point observations are shown in each panel, taken from 480 Sheepbed-formation measurements (Mangold et al., 2015). Also shown are the mean compositions, and standard deviations of the means, of other types of targets observed nearby and earlier along the traverse. These include Murray, Mars soil, two groups of conglomerates, ChemCam target Pyramid Hills, and a group of massive targets from Bradbury rise. Stimson-formation compositions are shown as contours where each contour represents equal weighting in terms of density of samples (see text and Supplementary material). (For interpretation of the references to color in this figure legend, the reader is referred to the web version of this article.)

Fig. 21 shows relationships among and between the different ChemCam Bimbe targets in terms of major-element abundances. Mean abundances are plotted for all but the conglomerates; individual observation points are plotted for the conglomerates. The conglomerates do not have a homogeneous composition, but instead, scatter significantly. The nodular sandstones (2 targets) also scatter, but are still plotted as means. Each of the other Bimbe groups (massive, vitreous-luster conglomerate clasts, and layered) form loci of points that are circled. All four panels show that the massive, layered, and conglomerate groups are generally distinct and do not overlap each other in overall compositions.

#### 3.4.1 Layered rocks

The layered group, consisting of just two targets, Chinchimane and Oranjemund, cluster tightly with the exception of the SiO_2_ abundance, where the two targets differ by nearly 4 wt% (Table 1 and the two yellow dots in Fig. 21). They are characterized by low Si and Al, and relatively high Ti, Mg, and K. Their chemical similarity belies the fact that Chin-chimane is clearly layered and Oranjemund only has evidence of grain-scale lamination (Figs. 12, 14). The standard deviations between points in the respective rasters of observations on these two clasts also places them together; they are among the lowest of all of the ChemCam Bimbe targets (Table 2), suggesting that they are fine-grained (e.g., Rivera-Hernández et al., 2019). In fact, their standard deviations between observation points are almost within a factor of two of the standard deviations of 25 laser shots within each individual observation point (Supplementary material). These latter values speak to the homogeneity over the depth of the laser pits. While the pit depths are not generally known, based on laboratory experiments (Wiens et al., 2012; Maurice et al., 2016) they are generally <150 μm for the 30 laser shots used at each of the observation points reported here, suggesting that the average grain size of these targets may be on the order of a few tens of microns (cf. Rivera-Hernández et al., 2019). Because of the similarity in composition of these two targets, Oranjemund is classified as “layered sandstone” along with Chinchimane.

The mean abundances and standard deviations of all 15 ChemCam observation points on these two layered targets are, for SiO_2_, TiO_2_, Al_2_O_3_, FeO_T_, MgO, CaO, Na_2_O, K_2_O: 42.9 ± 2.3, 1.2 ± 0.1, 6.3 ± 0.6, 20.1 ± 0.6, 11.5 ± 1.1, 5.2 ± 1.2, 2.1 ± 0.2, 1.0 ± 0.4 wt%.

#### 3.4.2 Massive rocks

Inspection of the compositions of the massive targets in Table 1 and Fig. 21 shows that their compositions are quite similar. They have relatively high SiO_2_, Na_2_O, and FeO_T_ abundances, moderate CaO, and relatively low MgO. Aluminum is surprisingly low, given the silica and alkali abundances. Although low Al_2_O_3_ has been seen in Gale crater rocks before (e.g., Stolper et al., 2013), the Al_2_O_3_/(CaO þ Na_2_O þ K_2_O) ratios are even lower in the Bimbe massive targets, as will be discussed in Section 3.6.4. Table 3 shows that the Bimbe massive targets are enriched in Sr (387 to 673 ppm mean abundances) and Li (24–34 ppm) relative to the other Bimbe targets. In particular, the lowest Sr abundance of the massive targets (Sonneblom_CCAM, at 387 ppm) is more than double the next highest Sr value (Chinchimane, at 173 ppm; Table 3) among the Bimbe targets. Simple statistical evaluation of the compositions and deviations of the massive targets, from Tables 1 and 2, indicate that they belong together as a compositional group. Specifically, standard deviations of the major-element abundances of the different targets, from Table 1, are significantly less than the means of the standard deviations between observation points within each target, from Table 2, indicating the compositional spread is greater within each target than it is between the different targets for the massive targets.

The mean abundances of all 42 ChemCam observation points on massive targets are, for SiO_2_, TiO_2_, Al_2_O_3_, FeO_T_, MgO, CaO, Na_2_O, K_2_O: 54.3 ± 6.3, 1.1 ± 0.5, 8.2 ± 3.4, 17.1 ± 3.2, 2.5 ± 1.0, 4.7 ± 3.2, 4.6 ± 0.9, 1.1 ± 0.6 wt%.

The Sonneblom and Zambezi APXS targets (Table 4) were on different areas of the same block. They are compositionally similar to one another. Slight differences in composition can be attributed to the varying dust coverage for the two different targets. The Sonneblom target was on the top, dusty surface of the rock, and the Zambezi target was on a darker gray, cleaner, inclined surface (Fig. 10a). The increased dust on the Sonneblom surface results in somewhat higher FeO, MgO, CaO, SO_3_, Cl, Ni, Zn, and Br concentrations than for the cleaner Zambezi target. Zambezi has relatively high SiO_2_, Na_2_O, and K_2_O concentrations. Al_2_O_3_, MgO, CaO, SO_3_, and Ni contents are relatively low. Comparison of ChemCam compositions with that of APXS (Table 4) for Sonneblom (Fig. 10)–the one target that was analyzed by both instruments–shows similar compositions. All of the major elements observed by APXS are within one standard deviation of the ChemCam compositions except for MgO, Na_2_O, and K_2_O. The MgO abundance observed by APXS is significantly higher than that of ChemCam, and Na_2_O is lower, possibly due to dust. APXS’s Zambezi observation appears more dust-free, and its Mg and Na abundances are close to those of ChemCam. An explanation for the substantially higher K_2_O observed by APXS at both locations of this block is unknown.

#### 3.4.3 Conglomerates

Conglomerates show significant compositional scatter in Fig. 21 and Tables 1–3, as expected for a group of cemented clasts from diverse sources, and consistent with previous conglomerates observed by ChemCam LIBS (Williams et al., 2013; Mangold et al., 2016). As mentioned above, two sub-clasts with vitreous lusters were sampled by ChemCam as individual targets within the conglomerates; these are Wilhelmstal and Cabamba. The compositions of these two targets (e.g., Fig. 18d, e) are each quite homogeneous (Table 2), and the two targets have similar compositions (Table 1 and Fig. 21) for all elements except Mg, where the mean MgO abundances range from 4.9 to 7.2 wt%. For the other elements, the differences between these two targets are generally smaller than the standard deviations within individual targets (Table 2), indicating significant homogeneity among the two. (Note, however, white grains in Fig. 18d, e that may not have been sampled by ChemCam.) The source of this apparent vitreous-luster, homogeneous material is not known, but it could be produced by impact melting or partial melting of some of the conglomerate source material, resulting in a relatively homogeneous glass with a composition that is near the mean composition of the more heterogeneous conglomerate.

A third target, AEGIS_post_1400a (Fig. 14a, and Supplementary material), appears to be compositionally similar to the vitreous-luster conglomerate clasts: Its composition is almost identical to that of Wilhelmstal, and also to that of Cabamba with the exception of MgO. This is in spite of having a quite different texture: The AEGIS target is not a conglomerate or part of a conglomerate; it is rough at the sub-millimeter scale and covered by indistinct 5–10 mm diameter lumps (Fig. 11c, d). Some of the protrusions are angular while others are rounded. We will show in Section 3.6.1 that its composition is that of the Stimson formation.

Among the ChemCam conglomerate targets, Bungo (Fig. 18c) shows the lowest heterogeneity between points (Table 2), besides the vitreousluster ones. It also has the highest FeO_T_ and lowest Al and alkalis (Table 1). The RMI image (Fig. 18c) shows no observable clasts in the targeted region. So a possibility is that Bungo’s composition represents matrix material. Bungo’s composition is somewhat different from the vitreous-luster conglomerate clasts in that it has lower Al and alkalis and higher Mg, although the standard deviations of Bungo and the vitreousluster conglomerate clasts overlap (Tables 1–2).

Table 2 shows that, except for Bungo and the vitreous-luster conglomerate clasts, the standard deviations within each individual conglomerate target are large, at least for Si, Al, Fe, Ca, and the alkalis. This might be expected, as the individual clasts within the conglomerates appear dissimilar. Thus the compositional variation agrees with the visual inspection, suggesting that these conglomerates contain clasts from a range of different host rocks. Seeheim (shown in Fig. 18b) in particular has high standard deviations (Table 2) due to several observation points apparently hitting nearly pure feldspars, with alkali totals in the 6–9 wt% range (Supplemental material). As noted above, nearly every point hit a different clast within the conglomerate.

The mean abundances of all 44 ChemCam observation points on conglomerates are, for SiO_2_, TiO_2_, Al_2_O_3_, FeO_T_, MgO, CaO, Na_2_O, K_2_O: 45.9 ± 5.3, 1.0 ± 0.1, 11.1 ± 2.6, 18.4 ± 3.2, 6.4 ± 2.0, 6.4 ± 2.4, 3.3 ± 1.0, 0.5 ± 0.5 wt%.

The compositions of individual ChemCam observation points of the conglomerates revealed other details of the chemistry, including a fluorine-rich point. This is described briefly in the Supplementary material Section 5.

Embedded in a conglomerate, the banded clast Funda and the surrounding matrix and clasts (Fig. 19a, b) were analyzed only by APXS. Funda raster-spot 3 had a significant proportion of the APXS field of view filled with the white clast, and the chemical data (Table 4) suggest that it consists of calcium sulfate. Funda could be either a mineral fill in a former void, or it could be a calcium-sulfate clast liberated from a previous rock and deposited with the sands and pebbles observed in the boulder. The remaining APXS raster spots do not have appreciable calcium sulfate, but reveal appreciable MgO, CaO and Na_2_O contents and relatively low K_2_O concentrations.

#### 3.4.4 Nodular sandstones

Targets Auchab and Canico are classified as nodular or coarse sandstone based on their textures (Figs. 13, 16). These two targets were found at different locations, both separated from the main group of Bimbe targets (Fig. 6b), and Auchab having been observed at a relatively long distance of 5.2 m. The two appear to differ in their FeO_T_ and K_2_O abundances (Table 1), with Auchab being higher in the latter and Canico higher in FeO_T_. However, the standard deviations of the points (Table 2) are relatively high, so that the Fe difference is not significant in terms of the overall rock composition. ChemCam’s K_2_O abundances have been observed to be higher for targets at distances > 3.5 m; Auchab is in the 95th percentile in terms of target distance (Maurice et al., 2016), so the K_2_O difference is not likely significant.

To investigate briefly the chemical make-up of the nodules, we look at the compositions of the individual points (Supplementary material). From the Canico data, Fe is anti-correlated with Si, Al, Ca, and Na; it is uncorrelated with Mg, and Fe is positively correlated with Ti (R^2^ = 0.73). Qualitatively it is also positively correlated with Mn, although the Mn results are not elaborated further in this paper. Auchab, at more than twice the distance from ChemCam, generally shows the same trends, but with greater scatter. The images (Figs. 13, 16) show that Auchab has a far higher density of coarse nodules. The generally higher Fe abundance of Auchab (Table 1) might suggest that the nodules are high in Fe and Ti, although it is difficult to make any stronger inferences from this limited data set.

The mean abundances of all 10 ChemCam observation points on these two nodular sandstone targets are, for SiO_2_, TiO_2_, Al_2_O_3_, FeO_T_, MgO, CaO, Na_2_O, K_2_O: 42.9 ± 4.4, 0.9 ± 0.1, 10.7 ± 3.2, 24.1 ± 4.9, 6.1 ± 2.1, 5.1 ± 1.1, 3.3 ± 1.0, 1.3 ± 0.7 wt%.

Mariental’s sedimentology (Fig. 20) was noted to be different from other conglomerates targeted by ChemCam, and its chemistry differs somewhat as well. Mariental’s CaO abundance is higher than any of the conglomerates and massive float rocks. Mariental’s silica abundance is in between that of the conglomerates and the massive targets. Its composition is more iron-poor than the conglomerates (Table 1), and it lies within the relatively tight FeO_T_ range of the massive float rocks. Its MgO is relatively low compared to the conglomerates, but definitely higher than the massive float rocks. Mariental is also distinguished by the highest Al_2_O_3_ of any of the Bimbe clasts analyzed by ChemCam. From these differences we might conclude that Mariental differs in origin from the more bedded conglomerates at this Bimbe location.

### 3.5 Blackfoot and Brandberg compositions

Compositional data from Blackfoot and Brandberg are sparse. Of the few targets observed by ChemCam, most either clearly belong to the Murray or Stimson formations or else they were at relatively long distances of ~4.5 m which, as discussed above, is near the limit of where the calibration can be trusted. At the edge of Brandberg, targets Roter Kamm and Hoba (Fig. 5b) are identical in composition to Murray bedrock (Supplementary material), although Hoba lies within the Brandberg unit. Target Gibeon (Fig. 5c) was shot at longer distance and because the autofocus was made along a receding edge of the rock, the observation points were mostly out of focus. This dark-gray float rock appears to have dark nodules protruding on its right side. Observation point 3 hit a dark patch and shows a strongly enriched FeO_T_ content. This observation might classify Gibeon with the Bimbe nodular sand-stones, although more details on the composition are lacking.

At Blackfoot, the ChemCam targets at reasonable observing distances included Sunburst, Jefferson, and Lincoln (Tables 1, 2, 3; Figs. 2a, 3a, b, e), observed on sols 1100 and 1102. Of these, Sunburst corresponds to a relatively rare group of Stimson-formation targets referred to as Cluster 1 by Bedford et al. (2020). Sunburst has high MgO, like the Bimbe and Bradbury layered targets, but its K_2_O is much lower, averaging 0.2 wt% compared to 1 wt% for the other layered targets. Additionally, Sunburst shows no detectable Rb, and its Sr is nominally less than two thirds of the Sr abundances of Chinchimane and Oranjemund (Table 3).

Jefferson (shown in Supplementary material) is the only ChemCam-sampled example of the enigmatic flat-topped boulders (such as the ones that appear light-toned from orbit, e.g., Fig. 2c). Its composition bears some similarities to Sunburst, both having relatively low SiO_2_ (Table 1). However, considering both of the layered clasts at Bimbe and (possibly) Sunburst at Blackfoot, Jefferson is an outlier in most of the major elements. It has higher Al_2_O_3_ and Na_2_O, and lower MgO and K_2_O, though as noted above Sunburst has even lower K_2_O (Table 1). Additionally, Table 2 shows that Jefferson has greater variance among its ten observation points. This is especially true for Al_2_O_3_ and MgO, where the standard deviations are several times higher than those of the layered sandstone targets. The higher standard deviations suggest that Jefferson has a larger grain size than the other layered sandstone targets.

Lincoln’s (shown in Supplementary material) composition is somewhat similar to Jefferson’s but the differences are in a direction away from those of the layered targets. It has a slightly more felsic composition (higher Si, Al, and alkalis, and lower Mg; Table 1).

The target Madison (Fig. 3e) was observed at a distance of 4.5 m. Four of the five points were on Ca-sulfate material adhering to the side of the rock. The one point that appeared to hit the relatively dark gray rock itself has a felsic composition with 55 wt% SiO_2_, 17 wt% Al_2_O_3_, 4.6 wt% Na_2_O, and 2.2 wt% K_2_O, along with moderate FeO_T_. This would place it near the Bimbe massive-rock composition except the Al_2_O_3_ and K_2_O are both way too high for the Bimbe Massive group. Additionally, Madison’s Sr abundance is near zero, below the RMS error of 85 ppm, far below the mean Sr abundance for the Bimbe massive targets (Table 3), suggesting more strongly that Madison is different from the Bimbe massive targets. Another target, Swan (shown in Supplementary material) was observed by ChemCam on the Blackfoot unit, but its composition is identical to that of Stimson bedrock. The one APXS target within the Blackfoot deposit, Badlands, has an average Mars-like composition, similar to many Stimson-formation APXS targets, but with elevated K_2_O and Na_2_O.

We conclude that a number of Brandberg and Blackfoot ChemCam targets have compositions synonymous with Murray and Stimson-formation rocks, while several targets, including Jefferson, Lincoln, and Madison, and the APXS target Badlands, do not clearly match any other compositions.

### 3.6 Compositional comparisons to other Gale clasts

The Bimbe sediments appear to be overlying the eroded surface cutting across strata of the Murray formation, and they are within a couple hundred meters of Stimson-formation outcrops. Fig. 21 shows that none of the targets observed using ChemCam are close in composition to that of the Murray formation; the same is true for the APXS targets (although, as noted in Section 3.3.3.3, based on appearances in the images, there are cobbles and pebbles at Bimbe which are likely derived from the Murray formation). The Murray targets taken for comparison in Fig. 21 are from Hartmanns Valley, located near the Bimbe unit (Supplementary material, Section 3). Murray compositions varied somewhat across the ~350 m of vertical elevation between the lowest and the highest portions investigated to date. A significant change was the reduction in CaO as the rover ascended higher in the Murray formation (e.g., Mangold et al., 2019). However, CaO is not critical to distinguishing between groups of Bimbe targets or establishing a connection to Murray materials. Overall, comparisons with other targets observed during the mission suggest some specific similarities for the conglomerates and the layered targets, which we discuss in turn below.

#### 3.6.1 Conglomerates

Here we compare the Bimbe conglomerates to those observed earlier in the mission. We limit this discussion to ChemCam observations. Mangold et al. (2016) compiled the compositions from 197 observation points on conglomerates encountered early in the mission on Bradbury rise prior to Sol 540; these were called “Darwin type” in their paper. The same work characterized a number of conglomerates from between sols 540 and 670 in the vicinity of the Kimberley waypoint (Fig. 1). These have distinctly different compositions, being alkali-rich with Na_2_O/K_2_O < 2.0. The mean value (not shown) of all Bimbe conglomerate and nodular sandstone observation points is nominally lower in Al_2_O_3_, Na_2_O, and K_2_O and substantially higher in MgO relative to both Bradbury and Kimberley conglomerates (Fig. 21). Further, the Bimbe conglomerate compositions do not appear to lie along any kind of mixing trend that would relate them to the other two types of conglomerates. This is especially apparent with the alkali elements (Fig. 21b), where a trend through Kimberley and extending through Bradbury conglomerates with lower K_2_O would require higher Na_2_O for Bimbe, but Bimbe conglomerates instead have substantially lower Na_2_O. One might consider, very generally speaking, that Bimbe conglomerates are basaltic, close in composition to a Mars crust average (see details in Mangold et al., 2016), and on a trend between soil composition toward the mafic end, and Bradbury conglomerates toward the felsic end.

Geographically, the Bimbe unit is closer in distance to Kimberley than to the Bradbury conglomerates encountered near the beginning of the mission. The Bimbe conglomerate compositions are in some respects closer to the average composition of Kimberley conglomerates for most elements, including SiO_2_ (45.2 wt%), Al_2_O_3_, MgO, and Na_2_O. However, K_2_O and, to some extent FeO_T_, spoil this potential relationship. At Kimberley, observations of sediment targets showed high K_2_O abundances (Le Deit et al., 2016; Thompson et al., 2016), as high as 5.7 wt% (Le Deit et al., 2016) with individual observation points showing even higher values, while the CheMin team reported a high proportion of the mineral sanidine (28% of crystalline material), which is inferred to be the carrier of the potassium (Treiman et al., 2016).

Overall, Bimbe conglomerates match far better with Stimson-formation composition than any other possibility. Fig. 21 shows the Stimson-formation targets observed by ChemCam using density contours based on >300 ChemCam observation points in the Stimson formation (Supplementary material). The ensemble of Stimson compositions could be described as slightly bimodal, with trends in the direction of more felsic or mafic compositions. The mafic end is anchored to some degree by the composition of modern-day soil (Fig. 21). This generalization does not fit entirely, as Stimson contours trend toward somewhat higher Fe and Mg than soil, even if the description fits for Si, Al, Na, and K. For some of the plots in Fig. 21, the Stimson contours and the conglomerate scatter pattern appear to match well (for example, CaO vs. MgO in panel c, and Na_2_O vs. K_2_O in panel b), while some of the conglomerate points are low for Al_2_O_3_ and SiO_2_ (panels a and d, respectively). By comparison, the targets classified as vitreous conglomerate clasts plot close to the densest part of the Stimson contour pattern in almost every case.

An equivalence test was run to compare the Bimbe conglomerates and Stimson compositions, as described in the Supplementary material. While the match appears relatively close in some panels of Fig. 21 and in boxplots in the Supplementary material, the test passes for generally mafic elements (Ti, Fe, Mg, Ca) plus K, but fails for alkali felsic elements (Si, Al, Na). With this result we cannot say that the two groups are clearly equivalent, but they could have the same sources, for example, if the conglomerates picked up a small amount of additional felsic material.

#### 3.6.2 Nodular sandstone

Regarding nodular targets Auchab and Canico (Figs. 13, 16), some areas of the Stimson formation are dense with concretions (Banham et al., 2018). The first concretion-rich sandstone of Stimson composition was encountered at Upheaval Dome, roughly 100 m north of Pahrump Hills (Fig. 1) in an outcrop that also contains some conglomerates (Williams et al., 2018). Overall, the Stimson concretions do not show much deviation from bulk rock composition but for FeO_T_ there are a number of extreme outliers to high FeO_T_ concentrations, particularly in the Naukluft locality (see the boxplot in the Supplementary material; Bedford, 2019; Bedford et al., 2020). These concretions may relate to preferential cementation of the sandstone. If this is true, the sandstone in the Stimson formation could be predominately cemented by iron oxides that formed from olivine diagenesis when Stimson was buried (Yen et al., 2017; Hausrath et al., 2018). Thus if Bimbe’s nodular sandstones are derived from Stimson then it would be possible that they could have Fe-rich concretions based on what has been analyzed and interpreted for the concretions at Emerson and Naukluft plateaus (Fig. 1). If these features do not distort any sedimentary structures (like laminations) then they are likely to be concretions, which would support this hypothesis.

#### 3.6.3 Layered rocks

**Fig. 22:**
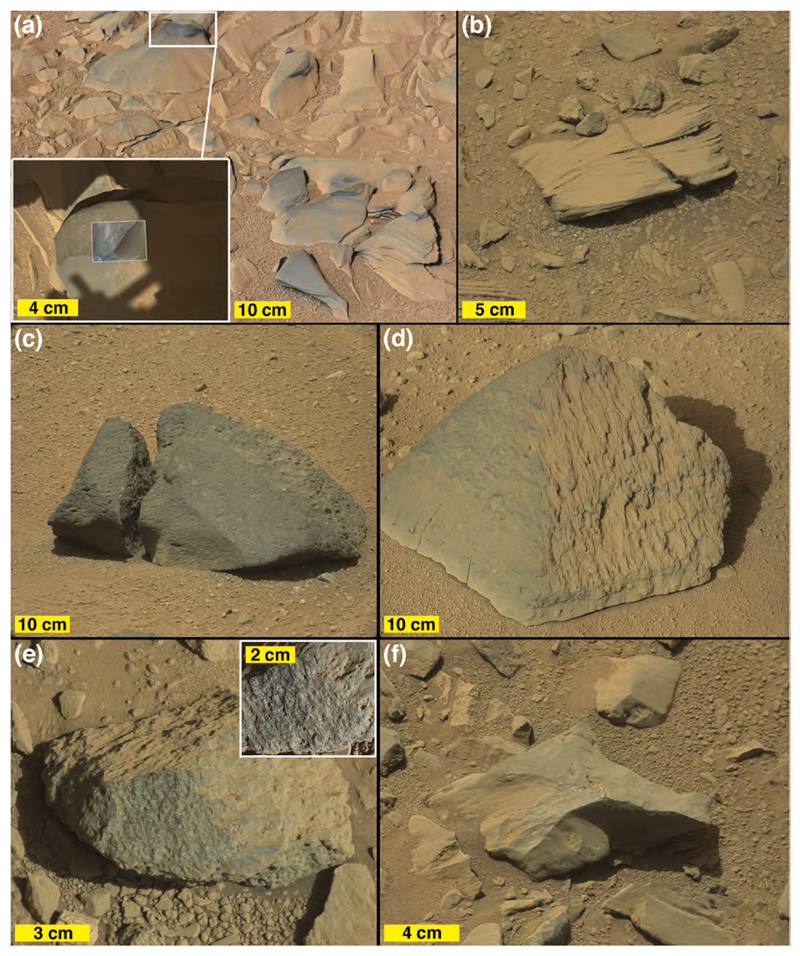
Rocks observed on Bradbury rise that match Bimbe float rocks in terms of morphology and composition in some cases, and only in morphology in other cases. (a) Bathurst_Inlet, observed Sol 55, and (b) Nullataktok (Sol 336), found in the same area, have similar compositions and morphologies to the Bimbe layered clasts. Bradbury massive targets (c) Bull Arm and (d) Jake_M do not match the compositions of the Bimbe massive targets, although they bear morphological resemblances. (e) Oscar (observed Sol 516) does match the Bimbe massive target compositions. (f) Pyramid Hills (observed north of Hidden Valley on Sol 692) is a close match to the Bimbe layered compositions, as well as to the Bradbury layered targets, e.g., (a) and (b). Locations of these targets are shown on Fig. 1.

Another apparent match occurs with the Bimbe layered sandstone group (Chinchimane and Oranjemund). Mangold et al. (2015) noted that a number of float rocks observed near the edge of Bradbury Rise (Fig. 22a, b)—both before the rover entered Yellowknife Bay and after it exited–form a compositionally and morphologically distinct group. The best known of these Bradbury layered rocks is Bathurst Inlet (Figs. 1, 22a), which was observed by Mastcam, MAHLI, APXS, and ChemCam on Sol 55 (e.g., Schmidt et al., 2014; Mangold et al., 2015). Other Bradbury layered sandstones of this class and in this area were chemically analyzed only by ChemCam (Mangold et al., 2015). The re-calibrated abundances of all 109 reported ChemCam observation points on these float rocks are given in the Supplementary material of Clegg et al. (2017). The mean compositions and standard deviations are plotted in Fig. 21. The match is relatively good in that the mean compositions of the two Bimbe layered targets are generally within the standard deviations even if they are not coincident with the mean Bradbury composition. The largest exceptions are Chinchimane’s SiO_2_ and Al_2_O_3_ abundances, both of which are low by a little more than the standard deviation of the Bradbury Layered group (Fig. 21).

**Fig. 23:**
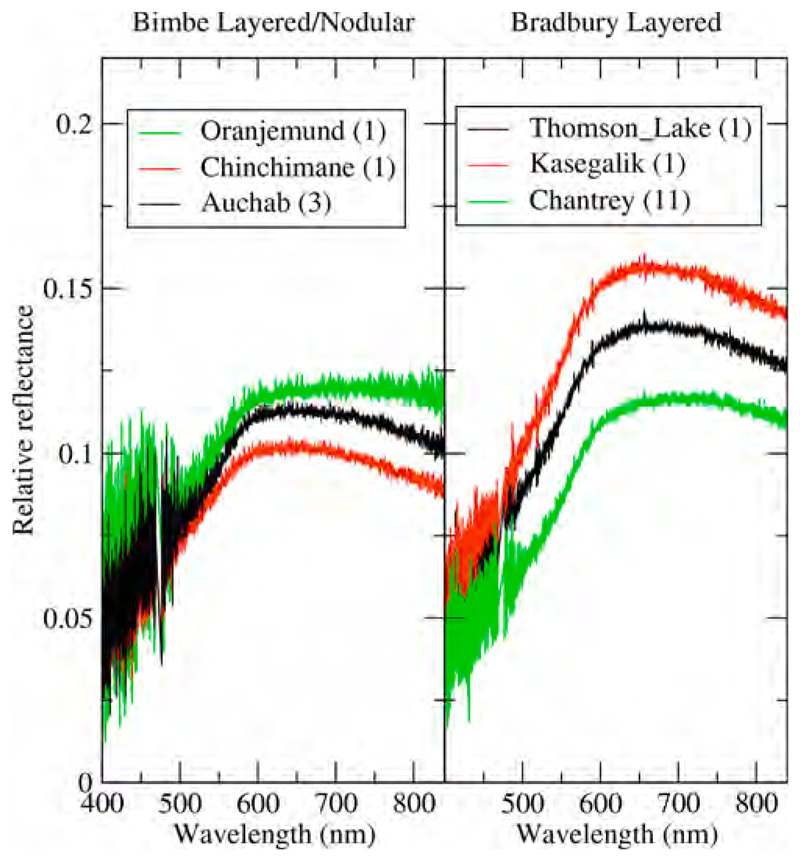
ChemCam relative reflectance spectra of representative samples of the Bimbe layered and nodular rocks, along with sample spectra from the Bradbury layered rocks. The Bimbe rocks show either flat near-infrared spectra or downturns that begin near 600 nm, whereas the Bradbury rocks have peak reflectance wavelengths near 650–675 nm. (For interpretation of the references to color in this figure legend, the reader is referred to the web version of this article.)

Fig. 23 shows ChemCam relative reflectance spectra of representative members of the Bimbe layered and nodular rock classes compared to members of the Bradbury layered rocks. Both exhibit flat to negative spectral slopes in the near-infrared. The Bimbe rocks Chinchimane and Auchab have peak reflectance positions near 600 nm, whereas all the Bradbury rocks exhibit longer wavelength peak reflectances (650–675 nm). This may indicate that the Bradbury layered rocks include lower-calcium pyroxenes and/or less olivine than the Bimbe rocks.

The equivalence test was also run on these two layered groups (Supplementary material). The groups fail the equivalence test for a number of elements (Ti, Al, Fe, Ca, and K), so while their compositions appear similar in Fig. 21 and in boxplots (Supplementary material Section 3), and they are unique in their Mg abundances, we cannot say they are equivalent. However, caution should be exercised with conclusions of this equivalence test due to the small number of Bimbe points (10), which is well below the recommended number of samples per group for this test.

At least one other ChemCam target along the traverse appears to have a close chemical relationship to the Bradbury and Bimbe layered compositions. It is Pyramid Hills (Sol 692; Fig. 22f), with SiO_2_ = 42.0, TiO_2_ = 1.1, Al_2_O_3_ = 5.6, FeO_T_ = 19.4, MgO = 10.8, CaO = 5.0, Na_2_O = 2.1, K_2_O = 1.0 wt% (Fig. 21). The standard deviations between points are also small, like the Bimbe layered targets (Table 2), and its morphology appears to fit this class as well. The Pyramid Hills rock face was in shadow, so its passive spectrum cannot be compared. A couple of other ChemCam targets in the area near Pyramid Hills (Fig. 1) may also be related. The significance of this match is discussed in Section 4.1.1.

#### 3.6.4 Massive rocks

The dark-toned massive float rocks of the Bimbe unit look most like some of the dark rocks observed at a topographic high named Twin Cairns Island (Sol 343; Fig. 1) approximately 0.5 km from Yellowknife Bay (e.g., Wiens et al., 2017). ChemCam targets there consisted of Black Trout, Bull Arm, and Mallard Lake (all observed on Sol 349; Figs. 1, 22c, and Supplementary material). Float rocks there and at Bimbe also bear a resemblance to the wind-eroded fine boulder named Jake_M (Figs. 1, 22d), observed by Mastcam, ChemCam, APXS, and MAHLI in the first 100 sols of the mission (Stolper et al., 2013). Jake_M’s composition falls within the range of the three targets from the Twin Cairns Island topographic high. The mean composition and standard deviation of the 33 ChemCam observation points on these Bradbury rise float rocks, including Jake_M, is plotted in each of the panels of Fig. 21 and labeled “Bradbury Massive”. It is apparent that the standard deviations are large for this group of targets, a feature that was already noted for Jake_M in Stolper et al. (2013); it was found to be quite heterogeneous even at the scale of the APXS footprint. Comparison of the Bimbe massive and vitreous conglomerate targets with the Bradbury massive composition shows that the Bradbury compositions appear to overlap the massive group in several respects, but the two are quite distinct in Al abundance, where the nominal Bradbury Al_2_O_3_ is ~17 wt%, while all of the Bimbe massive targets are below 10 wt%. Additionally, Bradbury massive targets are much lower in trace elements Li, Rb, and Sr. For example, the mean Sr abundance of the 33 points on the Bradbury massive targets is <200 ppm compared to a mean Sr abundance of 479 ppm in the Bimbe massive targets. Thus, the Bimbe massive targets, as seen by ChemCam, do not have the same compositions as, or similar compositions to, most of the massive targets on Bradbury rise. These two groups fail the equivalence test for all major elements but Mg (Supplementary material Section 4).

The APXS analyses of the Sonneblom/Zambezi boulder do, however, indicate a compositional similarity between this rock and an alkaline float rock, Oscar, analyzed by APXS on Bradbury Rise (Sol 516; Fig. 22e). Curiosity encountered a concentration of erosion-resistant, dark-toned float rocks on Bradbury rise between sols 503 and 526, and APXS analyzed nine of these, all of which are alkaline in composition. Other alkaline float rocks interrogated by APXS on Bradbury rise include the targets Jake_M (Fig. 22e) and Monkey Yard (Fig. 1). All the alkaline float rocks analyzed by APXS exhibit similar elemental trends: elevated Na_2_O, SiO_2_ and K_2_O, and depleted MgO, Cr2O_3_, MnO, FeO_T_ and Ni relative to average Mars. However, Oscar and Zambezi have higher SiO_2_ and FeO_T_, and significantly lower Al_2_O_3_ and CaO concentrations than the other alkaline float rocks. They are also somewhat depleted in Na_2_O, MgO, Cr_2_O_3_ and Zn relative to the other alkaline float rocks.

#### 3.6.5 Blackfoot and Brandberg

Of the Blackfoot and Brandberg targets, Lincoln (shown in Supplementary material) appears to be a reasonable match to Stimson-formation compositions. For the single observation point on Madison, its composition lies relatively close to the Bradbury massive targets for several elements (Table 1), but the Bradbury massive targets have much higher CaO, and at least one Bradbury massive target contained Ca-rich pyroxene (Supplementary material of Stolper et al., 2013). The one Brandberg ChemCam target, Hoba, looks like Murray formation in terms of composition and petrography; its composition fits well for the Murray formation except for somewhat high K, which is due to the observation distance (see comment in Section 3.4.4).

APXS observed one Blackfoot target, Badlands, on Sol 1102 (Fig. 3f). Its composition is given in Table 4, and is overall relatively similar to Stimson-formation rocks except for higher K and Na abundances. As such, it is considered to be of the Bell Island class, with somewhat lower K_2_O than Bathurst Inlet (Fig. 1), a layered float clast observed by APXS at Bradbury Rise (Schmidt et al., 2014).

## 4 Discussion

### 4.1 Compositional relationships to other Gale rocks and outcrops: possible implications

#### 4.1.1 Bimbe layered rock similarity to Bradbury layered rocks

The compositional similarity between the layered Bimbe targets and the layered Bradbury targets (Schmidt et al., 2014; Mangold et al., 2015) was noted above. Here we note their textural similarity. Only one Bimbe ChemCam target, Chinchimane, exhibits clear layering, while another target, Oranjemund, is compositionally very similar but only displays grain-scale laminations. Inspection of the rocks at Bradbury rise shows that the compositionally similar targets there exhibit both layering and no apparent layering. This is seen most clearly in Fig. 22a, where clasts at the lower right of the image are obviously layered but clasts from the same unit at the upper left, including APXS, MAHLI, and ChemCam target Bathurst Inlet (Fig. 1), are not obviously layered. This is consistent with the layered and non-layered Bimbe clasts with similar composition being related to each other; their morphological variations may suggest various levels of cementation. Additionally, Fig. 22b shows a Bradbury layered clast that is cross bedded.

The Bradbury clasts in Fig. 22a (Bathurst Inlet and surrounding clasts), while not connected as a single outcrop, appear to form a distinct unit relative to the surrounding gravelly surface material. The same feature appears to occur at the location of Pyramid Hills (Fig. 22f), where the ground appears to be covered with similar clasts. ChemCam observed two other targets—Johnnie and South Park2—that may belong to the same family (see their compositions and images in the Supplementary materials). In this location and at Bathurst Inlet near Yellow-knife Bay the rocks appear to be broken fragments of an intact rock unit.

The layered clasts in Bimbe (Sol 1401), Pyramid Hills (Sol 692), and Bathurst Inlet (Sol 55; Fig. 1) are not time-equivalent, given the significant difference in elevation (~100 m). Assuming that they are related, one possibility is that the rocks at Bimbe were sourced from a stratum or strata that was/were younger and located at a higher elevation than Bradbury rise, but have since eroded away completely. This stratum or strata that sourced the Bimbe rocks may have shared a similar provenance and depositional setting and process as the Bradbury layered targets.

Another possibility is that the layered rocks in Bimbe were deposited from the same source and at the same time as the Bradbury layered material. Considering the bedded nature of the material surrounding Bathurst Inlet on Bradbury rise (Figs. 1, 22a), this would require an unconformable unit that draped across the elevation difference between Bimbe and this portion of Bradbury rise, somewhat like the Stimson formation drapes over the Murray formation (Banham et al., 2018). Indeed these rocks could have been strata that were slightly younger than and overlaid the Stimson formation. Several factors lend credence to this idea. The first is that, based on ChemCam observations, the composition of bedded sandstones on Bradbury Rise, for example, at the Darwin and Cooperstown waypoints (Fig. 1), is nearly the same as those of the Stimson formation (Bedford, 2019; Bedford et al., 2020), suggesting that the Stimson formation may have extended farther from Mt. Sharp than the observable portion of the Murray formation (see Williams et al., 2018). This has implications for the timing of the deposition of rocks exposed on Bradbury Rise that is not discussed here. Secondly, the fact that the Stimson formation is already found to drape unconformably over the Murray formation lends some credence to the idea of additional, stratigraphically higher draping rock units. In this scenario, the draping formation that produced the Bradbury and Bimbe layered clasts would have almost completely eroded away, leaving only the rocks at Bathurst Inlet, some scattered layered clasts nearby (other layered float rocks in Mangold et al., 2015), the rocks at Pyramid Hills, and scattered layered clasts in another area having conditions that permitted long preservation, namely in the Bimbe unit.

#### 4.1.2 Bimbe massive sandstones and relationships to other clasts along the traverse

As described above, the Bimbe massive sandstones are not the same composition as most of their float-rock counterparts on Bradbury rise. The massive targets have been enigmatic ever since they were first reported (e.g., Stolper et al., 2013; Schmidt et al., 2014). Their compositions are consistent with a low degree of chemical alteration, but their textures are not igneous, appearing sedimentary instead. Massive Bimbe float rocks display some faint layering as well as apparent spherical grains (lower left portion of Seeis and inset, Fig. 11b). Similar to Jake_M on Bradbury rise (Fig. 22d), Seeis appears heavily wind scoured (Fig. 11), and the other Bimbe massive targets also show significant wind abrasion, suggesting that these clasts are very well cemented, if sedimentary in origin.

Besides Bradbury rise and the heterolithic units, there is one other location along the traverse, named Bressay (Fig. 1; studied on sols 2013–2023), where massive float rocks of similar appearance were studied. While it is beyond the scope of this work to describe Bressay, it is important to note that the clasts explored there by Curiosity are very different from those in the three heterolithic units described here. The areal extent of the Bressay deposit explored by Curiosity is far smaller, with most of the clasts within just 1 m of each other and a few other float rocks scattered a few meters away, although a larger extent to Bressay was imaged (Williams et al., 2020). The Bressay clasts investigated by Curiosity, all of which are smaller than boulders, include conglomerates, massive sandstones, and compositionally unique ones, including at least one apparent igneous clast (Bridges et al., 2019). The composition of the massive clasts does not match those in Bimbe, but instead appear to be close to those at Bradbury Rise. The compositions of the other clasts also do not match those in Bimbe. In summary, the Bressay clasts that were investigated appear unrelated to those of Bimbe, and based on size and appearance, are also unrelated to those of Brandberg and Blackfoot.

#### 4.1.3 Conglomerates with compositions similar to Stimson, and implications

The compositional similarity of the Bimbe conglomerates to the range of Stimson compositions (Fig. 21) is significant. As noted earlier, vitreous-luster clasts (sampled by targets Wilhelmstal and Cabamba) in conglomerates are fine-grained (as observed by ChemCam standard deviations of points), and compositionally they are located near the center of the locus of Stimson compositions. Other ChemCam observation points on conglomerates have larger ranges of compositions, but generally still fall within the Stimson contours, although the two groups fail the equivalence test for Si, Al, and Na. The ChemCam observations of the Stimson formation exhibit a range of compositions. As described by Bedford (2019) and Bedford et al. (2020) there are clear felsic to mafic trends among compositions observed in the Stimson formation. These are consistent with Curiosity observations of Bagnold dunes, where grain segregation is apparent (e.g., Cousin et al., 2017b; Ehlmann et al., 2017; Johnson et al., 2017; O’Connell-Cooper et al., 2017). Stimson formation is an eolian sandstone (Banham et al., 2018), and so the preservation of compositional variations reminiscent of grain segregation in contemporary dunes is not surprising.

The similarity in composition between Bimbe conglomerates and Stimson-formation rocks could imply either that their material comes from a similar or common source, or that the Bimbe conglomerate clasts are actually broken-up pieces of Stimson-formation sandstones, perhaps with some felsic clasts added. These two possibilities have quite different implications. A potential objection to the first possibility is that we do not see any obvious (significant) very local precursor material for the Bagnold dunes that exist currently (e.g., Ehlmann et al., 2017). If the same is true for the Stimson formation in spite of the different directions of their sources (Banham et al., 2018; Bedford, 2019; Bedford et al., 2020), then the observation of angular Stimson-composition pebbles in the conglomerates might be inconsistent with the lack of a local source. But if the clasts are broken-up Stimson sandstones, it would imply that the conglomerates were formed in fluvial action that came much later in time, after the Stimson-precursor dunes were buried and compacted, lithified, and then exhumed. That possibility, while seeming unlikely, cannot currently be completely discounted based on our limited data set.

The most likely possibility seems to be that there was in fact fluvial activity that was nearly contemporaneous with (e.g., either shortly before or after) the deposition of the Stimson eolian material and that this fluvial activity deposited conglomerates containing pebbles sourced from the same or similar material to that of the Stimson eolian material. That is consistent with some outcropping of conglomerates seen earlier, relatively near Stimson material (Williams et al., 2018). At some later point in time, some conglomerate material was broken up and transported to the heterolithic units, where it lies as described in this study. The next two sections (Sections 4.2 and 4.3) will discuss this latter step—how the material was transported to the heterolithic units and was apparently preserved.

### 4.2 Comparisons of the three units: similarities and differences

Here we consider all three heterolithic units, and their possible origins. But before going into models of deposition, we briefly summarize features of the three units, starting with their similarities: 1) the deposits form thin, isolated but coherent patches; 2) they are composed of unconsolidated pebbles, cobbles and boulders with varying degrees of roundness; 3) the patches include particles derived from the Stimson and Murray formations, and from other sources; 4) the deposits unconformably overlie Murray and (in one case) Stimson-formation bedrock; 5) the deposits occur on an eroded landscape, below adjacent local ridges and surrounding buttes, mesas, and plateaus capped by Stimson-formation rocks, and are not immediately adjacent to talus slopes from these local topographic highs, 6) there are similar boulder-rich patches in the region along the lower slopes of Mt. Sharp in HiRISE images (Fig. 6a), and 7) those patches are distributed in a band along the lower elevations of Mt. Sharp on slopes of about 5%.

Differences among the three units are highlighted in Table 5. Blackfoot is the only one of the three units that physically superposes outcrops of the Stimson formation. The lithologic diversity of Blackfoot and Bimbe includes conglomeratic boulders and cobbles. Whether conglomerates occur at Brandberg is uncertain; if they are present, they are small (cobble-sized) and rare. The conglomerates at Blackfoot and Bimbe may have different lithologies as compared with each other; those at Blackfoot are small, in a greater state of disintegration, and appear to have better-rounded pebbles within them. Some of the boulder- and cobble-sized stones at Brandberg are interpretable as Murray-formation mudstone lithic fragments; some of the cobble-sized stones at Bimbe are also interpreted as Murray-formation mudstones (though none that were analyzed for chemistry). In both cases, the mudstone fragments are similar in color and sedimentary texture to the nearby and adjacent intact Murray-formation outcrops. Blackfoot has no mudstone lithic fragments. Sandstones at all three units are dark gray and exhibit “massive,” “layered,” and “nodular” morphologies. Only at Blackfoot and Bimbe are light-gray and white sandstones observed (Figs. 3g, 15b, 16c)—possibly derived from fracture-associated “halo” areas in the Stimson formation. Only at Brandberg are concretions liberated from sandstones observed (Fig. 5d); these resemble the concretion forms observed via MSL cameras on the east side of the Naukluft Plateau (Fig. 5e, f).

A few more differences are as follows: Brandberg exhibits a circular shape and conforms to an arcuate ridge eroded into the adjacent Murray-formation mudstone (Fig. 4f). Some of the sandstone boulders and cobbles at Brandberg are slab-shaped, embedded in the deposit like lawn darts, and dipping steeply toward the center of the unit. Brandberg and Blackfoot are not cratered within their structures; the south edge of Bimbe is superposed by one, or possibly two, impact crater(s) (Fig. 8). The outline of Bimbe is irregular and its northeastern-most portion is a ridge. In summary, there are significant differences among the three heterolithic units visited by Curiosity.

### 4.3 Origin of the heterolithic units

**Fig. 24:**
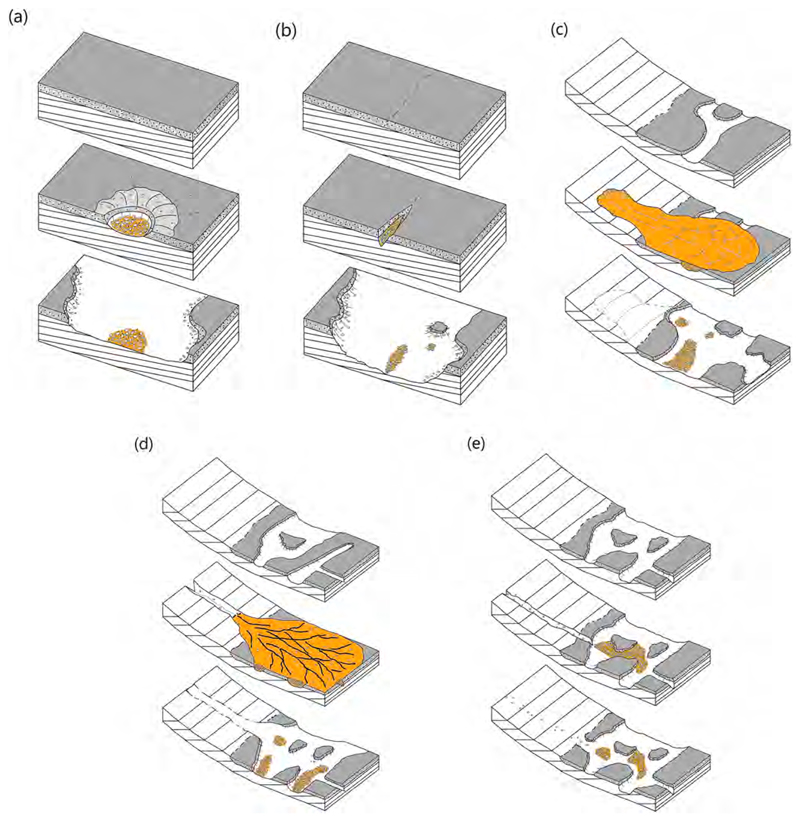
Possible scenarios for formation of the heterolithic units. Each panel shows three time steps. The gray stippled top layer is the Stimson formation, the white bedded unit is the Murray, and the orange records the heterolithic unit. (a) Impact penetrates through the Stimson formation into the Murray formation, creating a crater whose walls erosionally retreat through time. (b) Incision into a resistant Stimson cap rock (either by wind or water) that creates buttes and sheds Stimson and Murray blocks into a narrow valley. Further erosional retreat leaves isolated patches. Alternately, buttes decay in place to create the heterolithic units. Sediments are protected from weathering in the original location of the incision, or boulders shed from the mesas are collected in certain areas that are sheltered from wind erosion. (c) Mass transport of debris, either dry or by glacial processes, followed by extensive erosion that obscures the original source region. (d) Large-scale fluvial or debris flow fan deposits followed by erosion. (e) Localized fluvial or debris flow followed by erosion.

To explore the origin of the heterolithic units, we describe five different process sequences (models) that could have led to these distinct deposits. We use illustrations (Fig. 24) showing common elements: Each panel shows three time steps: an initial condition, delivery of the heterolithic sediment, and subsequent landscape erosion leaving isolated patches of heterolithic units. In every case, eolian abrasion causes erosion and deflation at variable rates around these units. In each illustration, the gray, stippled bedrock layer represents the Stimson formation which overlies the Murray formation (white with lines representing bedding) across an erosional unconformity. The subsequent heterolithic unit is indicated in orange. For the first two illustrations (Fig. 24a, b), dissection of the Stimson and Murray formations occurs simultaneously with the generation of local heterolithic units. The travel distance of sediments is short, on the scale of the preserved sediment pile, e.g., on the order of 50 m. In the subsequent three models (Fig. 24c–e), the initial condition is shown as a partially dissected landscape across lower Mt. Sharp that undergoes a pulse of burial followed by subsequent erosion which leaves isolated heterolithic units. In these three cases some sediment may be arriving from >10 km in the upslope direction, with an elevation drop > 1 km. The partially dissected initial condition localizes sediment deposition on eroded Murray- and Stimson-formation surfaces and exposes these rocks (and the no-longer-present conglomerate bedrock) to entrainment and incorporation into deposits that become the heterolithic unit.

#### 4.3.1 Model 1: crater impact and fill (Fig. 24a)

Impacts large enough to penetrate through the Stimson formation into the Murray formation at an earlier point in time (i.e., at a time when there was greater or complete coverage of the Murray formation by the Stimson formation) could have resulted in local accumulations of residual pebble, cobble, and boulder deposits. In this case broken rock of the Stimson and Murray formations would have collected on the floor from ejecta fall-back and subsequent erosional retreat of the crater walls. Wind erosion could then force retreat and elimination of crater walls and would eventually leave a coarse lag of material, covering the crater floor, that is more resistant to wind abrasion than the Murray bedrock. This model could explain the circularity of Brandberg, the arcuate ridge just outside of the unit (Fig. 4f), and the angular, steeply-dipping “lawn dart” rocks within the unit (Fig. 4d). The nearby clast observed by ChemCam (Hoba; Fig. 5a, b) has a Murray-formation composition and sedimentary texture, consistent with an expectation of local material falling into a crater. Gibeon (Fig. 5a, c) may be a remnant clast from the Stimson formation, and the clast in Fig. 5d is definitely similar to concretions that formed in the Stimson at the nearby Naukluft plateau (Fig. 5e, f). Other types of clasts may exist in Brandberg (e.g., conglomerates in Fig. 5g), depending on what overlying layers, above the Stimson formation, may have been present when the crater was formed. An impact crater of this size in the vicinity of dunes will fill with fine sediment, which may help preserve clasts from erosion for some period.

An impact origin is more difficult to fit with the overall shapes of Blackfoot and Bimbe, which are elliptical and irregular, respectively. Blackfoot could have been formed from an oblique impact, but if so, it does not retain the types of more telltale features seen at Brandberg, especially the surrounding ridge. Future exploration by the rover of craters excavated in Greenheugh Pediment (Stimson formation; Fig. 1) may shed light on the ability of craters in the overlying Stimson formation to explain the heterolithic units.

#### 4.3.2 Model 2: local valley incision (Fig. 24b)

This scenario bifurcates into two pathways, but both begin with valley incision through an erosion-resistant capping unit (Fig. 24b, first two time steps). The valley widens as scarps retreat. Such retreat could have occurred when the environment was wet enough to involve cap-rock undermining by seepage and removal of debris by streams. However, no landforms are present in the current landscape that would require a role for water in the retreat of these scarps (the same point applies to Model 1, above).

Progressive scarp retreat in a setting in which there is an erosion-resistant capping unit, like the Stimson formation, leads to formation of—in order of decreasing planimetric size—plateaus, mesas, and buttes (e.g., Duszyński et al., 2019). Such landscapes on Earth are usually connected to tectonism plus stream incision followed by scarp formation and retreat through a combination of weathering, creep, landslides, overland flow, and groundwater seepage (e.g. Twidale and Milnes, 1983; Howard and Selby, 1994). On Earth, boulders shed from the capstones of retreating cliffs typically do not extend far from the base of the slope (e.g. Glade et al., 2017). Shedding of material from, or disintegration of, the Murray buttes and precursors in the vicinity of the heterolithic units could potentially result in both Murray and Stimson clasts in the heterolithic units. The scarp in Fig. 24b is at least on the order of the height of the Murray buttes, e.g., 7–17 m. The local slopes from the Murray Buttes to the heterolithic units are not steep (Fig. 6b and Supplementary material).

##### 4.3.2.1 Shedding of boulders and cobbles from retreating mesa and butte scarps

One way by which scarp retreat could create a deposit like Bimbe, Brandberg, or Blackfoot is by the accumulation of boulders and cobbles shed from the slopes as they fail. In particular, this would lead to an abundance of the most erosion-resistant rocks, such as the Stimson-formation capping unit. If conglomeratic bodies of rock were present just above, below, or within the otherwise sandstone capping rock unit, they too, would be incorporated in the accumulation. The problem is that much of the space between Murray buttes or between the other scarps which expose the Stimson formation in the area is devoid of such boulders and cobbles. They mainly accumulate on and at the base of the scarp slope. As the scarps retreat, the destruction of boulders and cobbles apparently keeps pace with that retreat. Therefore, if this model is to explain the existence of a heterolithic unit such as Bimbe, it would require the accumulation of boulders and cobbles to be particularly thick, such that while destruction of boulders and cobbles might keep pace with scarp retreat, originally thicker accumulations of boulders at these sites has not yet been fully removed. Perhaps in the early stage of development, blocks accumulated across the valley floor and limited fetch (wind exposure) plus a rough valley floor reduced wind strength and thus abrasion, but once the mesa retreat was sufficiently wide, boulders that reached the base of the mesa were eliminated by wind abrasion. This concept could explain why boulders and cobbles are restricted to the specific heterolithic units where they are found. The coarse patches of the heterolithic sediment are not connected to the adjacent mesas.

##### 4.3.2.2 Decay and death of buttes

Another pathway to leave behind an accumulation of boulders and cobbles is through the decay and near-destruction of buttes. In this case, the boulders and cobbles would have been just barely displaced from their original positions as the capping rock was undermined and broke apart. The progression toward complete destruction of buttes is illustrated by Parzoch and Migon (2015) and Migon et al. (2018). In this case, we would expect the boulders and cobbles of a given heterolithic unit to be superimposed on a small, remnant hill of Murray-formation bedrock. The ridge at the northeast end of Bimbe, plus the two boulder- and cobble-mantled hills named Bukalo and Bailundo (Fig. 7) provide examples that such decayed buttes likely do occur in the landscape around Bimbe. However, the bulk of the area beneath Bimbe is not a low hill that could be interpreted as a decayed mesa or butte or collection of these (see topographic contours in Fig. 6).

#### 4.3.3 Model 3: landscape burial by mass transport of debris followed by extensive erosion (Fig. 24c)

Model 3 starts with a partially dissected lower Mt. Sharp landscape representing an area at least 10 km in length. Some features, specifically the heterolithic units, are not drawn to scale. A mass flow—either a massive landslide, rock glacier or glacier—descends from Mt. Sharp and spreads across the lower slopes, scouring and depositing debris on previously-exposed Murray and Stimson-formation surfaces. Subsequent wind erosion erases the trace of the scar and leaves isolated patches, most likely preserved for some time in depressions (craters, canyons, fractures). This model could account for Murray and Stimson clasts in the heterolithic units, as well as other varieties of clasts sourced from material father up Mt. Sharp. Various authors have proposed that the distinct lobate features on the middle slopes of Mt. Sharp, 20 km to the east of the Bimbe deposits, record landslides or glacial/periglacial features formed at a relatively late stage after Mt. Sharp had its present form (e.g. Anderson and Bell, 2010; Le Deit et al., 2013; Fairen et al., 2014; García et al., 2016).

A weakness of this model is the lack of evidence of either an upslope landslide scar or an accumulation zone for the supply to an advancing glacier in the ablation zone. Rocks along the rover’s traverse also lack glacial polish. However, the progressive erosional retreat of Mt. Sharp could have eliminated such evidence. Sediment sorting shown in Supplementary Fig. 2-4 suggests that the sediment deposit is not an unsorted matrix-supported mass typical of mass-flow deposits, but is instead similar to fluvial sorting, although the infrequent large boulders call for exceptional transport ability.

Periglacial (Anderson and Bell, 2010; Le Deit et al., 2013) and glacial (Fairen et al., 2014) processes have been proposed as possibly active in Gale in the past. Glacial moraines are characterized by poor sorting. The presence of conglomerates at Bimbe and Blackfoot could be explained from fluvial channels occurring beneath a glacier; some of the conglomerate clasts would have later been transported to and preserved in their current location. Another related possibility is that these units were deposited in a perennially ice-covered lake (PICL) that formed in Gale crater (not shown in the Model drawing). On Earth, sediments of varying compositions and sizes can accumulate on the ice cover of PICLs (e.g., Rivera-Hernández et al., 2018). For PICLs without liquid water columns (due to drainage, evaporation or sublimation of the liquid water), laterally discontinuous patches of sediment (i.e., sediment mounds) form as let-down till from the sublimation of the lake ice (Hendy et al., 2000; Hall et al., 2006). The sediment mounds consist of very poorly sorted mud to boulder sized grains. Sediment accumulated on the ice cover of PICLs can be substantial, for example Lake Miers in Antarctica has sediment piles up to several meters high, ten meters in width, and a couple tens of meters long (e.g., Bradley and Palmer, 1967). For PICLs abutting calving glaciers, let-down till can also occur as arcuate debris bands that extend across the former lake floor (Hendy et al., 2000; Hall et al., 2006). PICLs abutting calving glaciers and with liquid water columns, can also have ice-rafted poorly sorted sediments accumulate along the lake edge (which is ice-free during the summer) as a result of the ice cover being pushed toward the lake edge when glacial ice is incorporated into the lake ice (Hendy et al., 2000; Hall et al., 2006). Geomorphic evidence associated with PICLs abutting calving glaciers (Hendy et al., 2000; Hall et al., 2006) has not been observed in the vicinity of the heterolithic deposits either upslope or at the depositional sites.

#### 4.3.4 Model 4: landscape burial by laterally extensive fluvial or debris flow fan deposits, followed by extensive erosion (Fig. 24d)

This model proposes fluvial (water dominated) or debris flow (mobilized by mud supporting matrix) transport in which successive flows descend from Mt. Sharp and spread deposits across a broad area, burying the partially eroded landscape. The heavier black lines are meant to suggest successive channel pathways common to fan construction. Similar to Model 3 (mass transport), the deposited material is mostly lost to wind abrasion over time, leaving patches of sediment, most likely preserved in the original valleys where thicker deposits would have accumulated. Also similar to Model 3, this model would result in Stimson and Murray clasts as well as other material sourced farther up Mt. Sharp.

Transport of clasts in stream beds within Gale crater is now widely accepted to have occurred in the Bradbury rise region. Large amounts of igneous cobbles and pebbles of sizes up to ~10 cm were transported from the crater rim via Peace Vallis (Fig. 1 inset) and other channels (Sautter et al., 2014, 2015; Cousin et al., 2017a). HiRISE observations have also been made of fans deposits overlying the “washboard unit” (Siccar Point group) in other parts of Gale crater (Milliken et al., 2014). Grain size analyses reported for the three heterolithic units and shown in Supplementary Fig. 2-4 are similar to gravel-bed rivers on Earth. Median grain sizes range from 9 to 16 mm. The heterolithic-unit grain-size distributions are influenced by abrasion and weathering breakdown of large particles, partial burial by sand, and possible lag concentration due to wind erosion. The gravel and boulders are subangular (Powers visual scale), although ventifact shaping of the surface particles is common and obscures the original shapes of many of the larger boulders. The limited exposure on the edge of the deposits does not show matrix-supported clasts (as typifies debris flow deposits).

The prominent boulders that stand out in the heterolithic units are 30 to 40 times larger than the median size of the units, and thus would probably not be mobile in flows moving the finer gravel making up most of the deposits. Two explanations are possible for this admixture of very coarse rocks with finer gravel. The flows could have originated >5 km away on the slopes of Mt. Sharp and swept into previously partially-dissected landscape where talus from retreating mesas could be locally incorporated in the flows. Boulders may be entrained and then carried a short distance into the passing flows. Alternatively, the flows may have incorporated sufficient fines to have densified and transformed into debris flows, which could readily carry such boulders on gentle slopes in thin flows (e.g. Whipple and Dunne, 1992).

The laterally extensive occurrences of other apparent heterolithic deposits seen from orbit (e.g., one in Fig. 6a), if indeed these are genetically related to the three heterolithic units studied here, support a laterally extensive burial of the foot of Mt. Sharp. The description and study of these other units observed from orbit are beyond the scope of this paper. As mentioned for Model 3, no observations were made of deposits on the Stimson-capped mesas that would suggest such massive burial.

#### 4.3.5 Model 5: localized fluvial or debris flow transport down dissected plains (Fig. 24e)

The primary difference between this model and Model 4 is the proposed extent and thickness of the depositional field. In this model, localized avenues of runoff and sediment transport extend down a dissected lower Mt. Sharp landscape. Transport could be fluvial or debris-flow dominated or a mixture. In this case, the deposits would be initially relatively thin, and confined in valleys. Post-burial valley-wall retreat and valley-floor dissection by wind abrasion led to the localized, elevated patches of sediment. Preservation of these coarse deposits may be attributable to exceptionally low wind abrasion rates (the landscape was already mostly dissected to its current state by earlier longer-duration eolian abrasion) and the relative strength of the stones in the deposits compared to the underlying Murray formation.

#### 4.3.6 Discussion

The topographic setting of the Blackfoot, Brandberg, and Bimbe suggests a localized transport and deposition origin for the deposits. The Bimbe deposit lies in a ~300 m wide, nearly flat-floored sloping valley (Supplementary Fig. 1-3). Further to the north is a deposit that from HiRISE images is very similar in appearance (“Bimbe-like surface” in Fig. 6a; Supplementary Fig. 1-3). Over a ~500 m length, starting up slope of Bimbe and ending with the northern deposit, the valley slope is about 5% (Supplementary Fig. 1-3). Fluvial gravels, confined in channel banks in river canyons, are typically coarser than 16 mm on such slopes (e.g. Dietrich et al., 2017), but on fans, these median grain sizes do occur on such steep slopes (Stock et al., 2007). The current widths of the deposits are much greater than any likely individual channel. One possible interpretation is that a succession of flows traveled downslope to this area, spread, shallowed and deposited the sediment, as a fan-like deposit bordered by mesa walls. A similar argument can be made for Blackfoot, which forms a diagonal ridge connecting a row of southwest-northeast directed ridges of bedrock bordering its southern and northern edge (Supplementary Fig. 1-1). It is an erosional remnant with scoured depressions ~4 m deep to the west and east. It rests on Murray-formation rocks to the south and rises onto Stimson-formation rocks at its southern edge. The Brandberg deposit is the same elevation as Blackfoot (Supplementary Fig. 1-2). A transport path would have followed a partially etched surface into the Murray, bordered by Stimson formation-capped ridges, some of which were not deeply incised.

The three investigated deposits lie downslope from Gediz Vallis, associated ridge deposits, and Greenheugh pediment (Fig. 1; e.g., Bryk et al., 2019). The stratigraphy and apparent coarse texture of the Gediz Vallis ridge deposit suggest it may be the remnant of a fan of fluvial or debris flow origin. It is possible that the deposits once extended further downslope and that distal flows may have traveled the ~3 km from there downslope to the Bimbe-Blackfoot locations either during fan construction or during subsequent erosion. It was at one time a ready supply of sediment, and could also be a source for lithologic heterogeneity of the sediment during its erosion. This scenario requires that at least a portion of the Gediz Vallis sedimentation postdated lithification of the Stimson formation. The flow would have occurred before the existence of the shallow valley (Glen Torridon; Fig. 1) separating Greenheugh pediment and VRR. The relatively short travel distance to Bimbe would likely cause only limited rounding of sediment (Szabo et al., 2015) during their transport and deposition as collections of float rocks.

More than one model may have given rise to the three heterolithic units. For example, the round shape of and arcuate ridge around Brandberg (Fig. 4f) seem to be indicators of an impact crater but Blackfoot and Bimbe seem to require other factors. The fact that Bimbe and its non-visited companion to the north (Fig. 6a) lie in a broad valley (Supplementary Fig. 1-3) may be a clue, but Brandberg and Blackfoot do not share that characteristic.

The bare, unconsolidated appearance of the sediment in these units, showing clear signs of wind abrasion, raises question of whether these thin deposits were, for a period of time, buried by sufficiently thick deposits of sand (like that in the Bagnold dunes) that exposure to wind erosion was greatly reduced, as active erosion proceeded to cut widened valleys, craters, or local troughs into the underlying Murray formation. Alternatively, the wind-erosion rates may be exceptionally slow on the relatively hard stones and be diminished due to the roughened surface produced by diverse grain sizes. In either case, the current three patches are generally elevated slightly relative to some of their immediate surroundings, indicating greater resistance to erosion than the underlying Murray.

## 5 Conclusions

Exploration of the three heterolithic units has revealed a number of unique details. Significantly, the presence of conglomerates with compositions that are very close to that of the Stimson formation implies that some wet epoch must have existed after the Murray formation was eroded to nearly its present surface, and during or after the time period that the eolian material was deposited to form the Stimson formation. This must have been significantly later than the lacustrine activity associated with the Murray formation, thus representing a “later wet period” in Gale crater (see Palucis et al., 2016). The conglomerates would have been formed from fluvial activity that was relatively close in time to the deposition of the Stimson eolian strata. (However, these eolian strata are notable for their lack of evidence of wet conditions; Banham et al., 2018.) At some later time the conglomerate outcrops would have been broken up and transported to where they are now found in the heterolithic units.

The chemical similarity of float rocks explored early in the mission, classified as Bradbury layered (Mangold et al., 2015), to two stones of similar appearance at Bimbe and at least one located midway between these locations, raises the possibility of the previous presence of overlying strata, above the Stimson formation and extending northward across Bradbury rise. These rocks are notably elevated in Mg and K. These strata, if they existed, would likely have been part of the Siccar point group, as is the Stimson formation (which also shows some elevation of Mg and K, but not to the higher values observed in these disparate samples from Bathurst Inlet to Bimbe).

The dark, gray massive boulders and clasts of Bimbe analyzed by ChemCam are not the same compositions as a number of similar-appearing boulders and smaller rocks at Bradbury Rise. However, the alkaline float rock, Oscar, analyzed on Sol 516 by APXS and ChemCam, is essentially identical in composition to these Bimbe targets.

In terms of the origins of the heterolithic units, we explore five different models that attempt to explain various attributes of the three units explored by Curiosity. Brandberg shows quite strong evidence for starting as an impact crater, while the other two units lack such evidence, suggesting that they may have formed by different means. Particle size analysis suggests a possible fluvial origin for the deposits, but the large boulders suggest a role played by debris flows. A common thread seems to be preservation of the sandstone clasts, cobbles, and boulders by the deposition of soils in the proposed depressions that may have characterized these units at an earlier point in time.

On the observational side, large blocks with relatively flat skyward faces can result in higher apparent albedos from orbit than blocks of the same composition and morphology that are more rounded and not skyward facing. Care must be taken in interpreting the albedos of blocks observed from orbit.

## Supplementary Material

Supplementary data to this article can be found online at https://doi.org/10.1016/j.icarus.2020.113897.

Supplemental material Word

Supplemental material PDF

## Figures and Tables

**Table 1:** Mean relative abundances in wt% of major elements for ChemCam targets in the Bimbe and Blackfoot units.

	Sequence	# points	Dist. (m)	Group	SiO_2_	TiO_2_	Al_2_O_3_	FeO_T_	MgO	CaO	Na_2_O	K_2_O	SUM
*Bimbe targets*													
Aussenkehr	01401	10	2.61	Massive	51.5	1.06	8.2	17.5	1.9	5.1	4.3	1.1	90.7
AEGIS_post_1406a	15900	9	2.88	Massive	56.9	1.00	8.6	17.9	2.6	3.1	4.8	1.1	95.9
Lucala	01407	5	2.62	Massive	55.4	1.41	8.8	15.8	2.3	3.4	4.9	1.3	93.4
Sonneblom_CCAM	04407	9	2.25	Massive	54.4	0.90	7.0	17.3	3.0	5.2	4.6	0.8	93.3
Seeis	04409	9	2.39	Massive	54.2	1.08	8.7	16.2	2.5	5.9	4.6	1.1	94.4
Bungo	03407	10	2.54	Conglomerate	44.2	0.92	9.8	19.5	7.2	6.7	2.7	0.2	91.3
Balombo	05407	10	2.83	Conglomerate	43.9	0.92	10.3	18.2	7.1	7.5	3.0	0.7	91.6
Seeheim	01409	5	2.52	Conglomerate	51.2	0.89	13.2	13.7	4.5	6.1	4.4	1.1	94.9
Wilhelmstal	02409	9	2.53	Conglom. vitreous	45.5	0.95	11.0	19.2	7.2	5.8	3.4	0.4	93.4
Cabamba	02407	10	2.47	Conglom. vitreous	47.2	1.04	12.2	19.2	4.9	5.7	3.5	0.6	94.2
AEGIS_post_1400a	15900	9	2.59	Knobby SS (Stimson)	45.3	0.86	11.4	19.1	7.7	5.7	3.1	0.5	93.8
Auchab	01400	5	5.20	Nodular SS	43.5	0.95	11.4	26.4	7.2	5.0	3.2	1.8	99.5
Canico	02401	5	2.52	Nodular SS	42.3	0.95	10.0	21.9	4.9	5.3	3.3	0.7	89.4
Chinchimane	03401	5	2.28	Layered	40.4	1.17	6.1	20.2	11.7	5.2	1.9	1.1	87.9
Oranjemund	03409	10	2.95	Massive/layered	44.1	1.17	6.5	20.1	11.3	5.2	2.1	0.9	91.3
Mariental	05409	5	4.17	Other	48.7	1.05	15.5	17.6	5.2	8.2	3.7	0.4	100.4
*Blackfoot targets*													
Sunburst	02100	10	2.36	Stimson Cluster #1	42.4	0.92	8.2	19.4	12.5	4.7	1.6	0.2	90.0
Jefferson	02102	10	2.94	Other	44.0	1.30	10.2	20.3	9.5	4.4	2.5	0.4	92.5
Lincoln	04102	5	2.96	Other	46.2	1.07	11.7	20.5	7.8	4.5	2.8	0.7	95.3

Dist. = distance; SS = sandstone; see text for definition of other terms.

**Table 2:** Standard deviations between observation-point compositions for major elements in wt% for ChemCam targets in the Bimbe and Blackfoot units.

Group	SiO_2_	TiO_2_	Al_2_O_3_	FeOT	MgO	CaO	Na_2_O	K_2_O	SUM
*Bimbe targets*										
Aussenkehr	Massive	4.5	0.50	2.8	1.9	0.7	3.4	1.0	0.5	5.3
AEGIS_post_1406a	Massive	7.3	0.28	3.9	3.1	0.8	1.8	1.2	0.5	6.5
Lucala	Massive	5.5	0.70	4.8	1.6	0.7	2.8	0.9	0.7	6.4
Sonneblom_CCAM	Massive	6.9	0.26	2.6	3.7	1.3	3.1	0.5	0.4	4.3
Seeis	Massive	6.6	0.56	3.9	4.5	1.0	4.3	0.7	0.8	4.7
Bungo	Conglomerate	2.1	0.09	2.6	1.1	1.3	1.0	0.4	0.1	3.1
Balombo	Conglomerate	6.8	0.13	2.6	2.5	2.5	4.3	1.2	0.3	5.9
Seeheim	Conglomerate	9.7	0.09	3.0	7.3	2.2	2.7	1.7	1.1	3.6
Wilhelmstal	Conglom. vitreous	1.7	0.06	1.0	0.7	0.9	0.6	0.4	0.1	2.1
Cabamba	Conglom. vitreous	3.7	0.07	2.6	1.6	1.4	1.1	0.6	0.3	3.4
AEGIS_post_1400a	Knobby SS (Stimson)	1.6	0.09	2.8	1.0	2.3	0.8	0.5	0.1	3.0
Auchab	Nodular SS	1.9	0.08	2.3	3.2	1.9	0.8	0.6	0.6	2.5
Canico	Nodular SS	6.2	0.15	4.1	5.6	1.8	1.5	1.4	0.1	5.3
Chinchimane	Layered	1.1	0.10	0.2	0.4	0.9	1.0	0.2	0.2	1.0
Oranjemund	Massive/layered	1.7	0.12	0.6	0.6	1.2	1.3	0.3	0.4	1.5
Mariental	Other	4.9	0.39	2.6	5.1	1.3	4.1	0.9	0.3	3.0
*Blackfoot targets*										
Sunburst	Stimson Cluster #1	1.5	0.17	0.8	0.7	0.8	1.3	0.2	0.1	1.9
Jefferson	Other	2.2	0.30	3.2	1.0	2.7	1.0	0.5	0.1	3.3
Lincoln	Other	1.9	0.37	2.7	1.1	3.3	0.8	0.5	0.3	2.6

**Table 3:** Selected trace-element abundances for ChemCam targets in the Bimbe and Blackfoot units. Abundances are in parts per million.

	Group	Li	Rb	Sr
*Bimbe targets*			
Aussenkehr	Massive	32.1	48.8	487
AEGIS_post_1406a	Massive	33.9	67.2	458
Lucala	Massive	23.6	74.5	673
Sonneblom_CCAM	Massive	27.9	<57.9	387
Seeis	Massive	27.8	69.2	473
Bungo	Conglomerate	10.3	<26.0	<98
Balombo	Conglomerate	14.1	<56.3	<133
Seeheim	Conglomerate	8.6	91.8	<167
Wilhelmstal	Conglom. Vitreous	10.7	<26.0	<103
Cabamba	Conglom. Vitreous	10.1	<42.0	<120
AEGIS_post_1400a	Knobby SS (Stimson)	20.1	61.4	<129
Auchab	Nodular SS	11.6	<39.5	<96
Canico	Nodular SS	8.9	<34.6	<168
Chinchimane	Layered	17.4	38.7	173
Oranjemund	Massive/Layered	11.8	52.2	152
Mariental	Other	21.3	<26.9	<115
*Blackfoot targets*^a^				
Sunburst	Stimson Cluster #1	14.6	31.0	<118
Jefferson	Other	10.1	28.7	<147
Lincoln	Other	14.9	–	<96

a Rb abundance for Lincoln was not computed; for Sunburst and Jefferson the results are each based on a single point.

**Table 4:** APXS compositions for targets at Bimbe and Blackfoot.

Element	Sonneblom	Stat.	Zambezi	Stat.	Funda1	Stat.	Funda2	Stat.	Funda3	Stat.	Funda4	Stat.	Badlands	Stat.
Na_2_O	3.92	0.14	4.52	0.2	3.2	0	3.14	0.14	3	0.14	3.21	0.14	3.25	0.14
MgO	4.55	0.17	2.64	0.08	7.27	0.17	7.72	0.17	7.09	0.17	7.26	0.17	8.42	0.25
Al_2_O_3_	9.22	0.19	9.23	0.19	10.42	0.29	10.38	0.29	9.35	0.19	9.5	0.29	8.86	0.19
SiO_2_	54.08	0.64	59.25	0.64	47.22	0.54	46.18	0.54	42.34	0.54	47.99	0.54	43.57	0.54
P_2_O_5_	0.71	0.05	0.57	0.05	1.18	0.07	1.09	0.07	1.03	0.07	1.23	0.07	0.74	0.05
SO_3_	4.3	0.05	2.87	0.05	3.22	0.12	3.23	0.12	9.5	0.22	4.22	0.12	5.08	0.07
Cl	1.02	0.02	0.71	0.02	1.11	0.05	1.31	0.05	1.3	0.06	1.33	0.05	1.52	0.02
K_2_O	2.61	0.08	3.17	0.1	0.61	0.04	0.49	0.02	0.51	0.04	0.62	0.04	0.93	0.04
CaO	3.83	0.04	2.96	0.04	6.55	0.01	7.04	0.01	9.18	0.13	6.83	0.1	5.86	0.07
TiO_2_	0.86	0.03	0.82	0.03	1.22	0.05	1.05	0.05	0.9	0.05	0.97	0.05	0.89	0.03
Cr_2_O_3_	0.08	0.01	0.03	0.01	0.28	0.03	0.26	0.03	0.26	0.03	0.25	0.03	0.39	0.01
MnO	0.22	0.01	0.21	0.01	0.35	0.03	0.39	0.03	0.29	0.03	0.33	0.03	0.40	0.01
FeO	14.37	0.2	12.69	0.13	17.22	0.2	17.56	0.2	15.12	0.2	16.14	0.2	19.87	0.26
Ni	0.012	0.001	0.0056	0.001	0.0239	0.0035	0.0227	0.0035	0.0294	0.004	0.0209	0.003	0.0485	0.003
Zn	0.0184	0.001	0.0169	0.001	0.0605	0.0035	0.0431	0.003	0.0334	0.0025	0.0437	0.0025	0.0449	0.002
Br	0.0208	0.001	0.0033	0.0005	0.024	0.0015	0.0245	0.0015	0.0274	0.0015	0.0209	0.0015	0.0277	0.001

Stat. ¼ statistical uncertainty (precision). Units are weight percent.

**Table 5:** Differing characteristics of the three heterolithic units in this study.

	Blackfoot	Brandberg	Bimbe
Rover studied on sols	1099–1104	1158–1160	1399–1410
Elevation	−4434 to −4432 m	−4435 to −4432 m	−4417 to −4426 m
Approximate size	1730 m^2^	1600 m^2^	16,800 m^2^
Shape	Semi-elliptical	Nearly circular	Irregular
Arcuate ridge at perimeter	No	Yes	No
Steeply-dipping sediments	No	Yes	No
Contains a several-meter ridge?	No	No	Yes
Superposes Stimson formation?	Yes	No	No
Stimson-like concretions?	No	Yes	Perhaps, in Auchab and Canico
Contains mudstone lithic fragments (possible Murray)?	No?	Yes	Yes
Light gray-white sandstones?	Yes	No	Yes
Contains conglomerates?	Yes, small, disaggregated	Possibly	Yes
