# Supplementary material for: Origin and composition of three heterolithic boulder- and cobble-bearing deposits overlying the Murray and Stimson formations, Gale Crater, Mars
Source: Icarus. Author manuscript; Available in PMC 2020 Nov 1. (PMC7326610; doi:10.1016/j.icarus.2020.113897)
Supplement: Supplemental material PDF [file EMS86594-supplement-Supplemental_material_PDF.pdf]

Supplemental Material

ORIGIN AND COMPOSITION OF THREE HETEROLITHIC BOULDER- AND COBBLE-BEARING DEPOSITS OVERLYING THE MURRAY AND STIMSON FORMATIONS, GALE CRATER, MARS

R.C. Wiens et al.

Contents

1. Figures Showing Contour Lines Near the Heterolithic Units.. 2
2. Description of Particle Size Measurements.. 6
3. Description of Reference Points and Contours in Figure 21, and Supporting Figures. 10
4. Description and Results of Equivalence Tests 16
5. Some Unique Compositional Features of the Bimbe Float Rocks 18
6. The Heights of the Murray Buttes 19
7. Additional References for Supplemental Section.. 21
8. Compositions of Individual ChemCam Observation Points, Bimbe.................................... 22
9. Compositions from Individual ChemCam Observation Points: Blackfoot, Brandberg 23
10. Images of ChemCam Heterolithic-Unit Targets Not in the Main Body of the Paper........ 24
11. Images of ChemCam Bradbury Targets Not in the Main Body of the Paper. 36
12. Images of Other Targets from Zabriskie Plateau (Johnnie, South_Park2)........................ 40
13. Designations of Images Used in the Paper 44

1. Figures Showing Contour Lines Near the Heterolithic Units

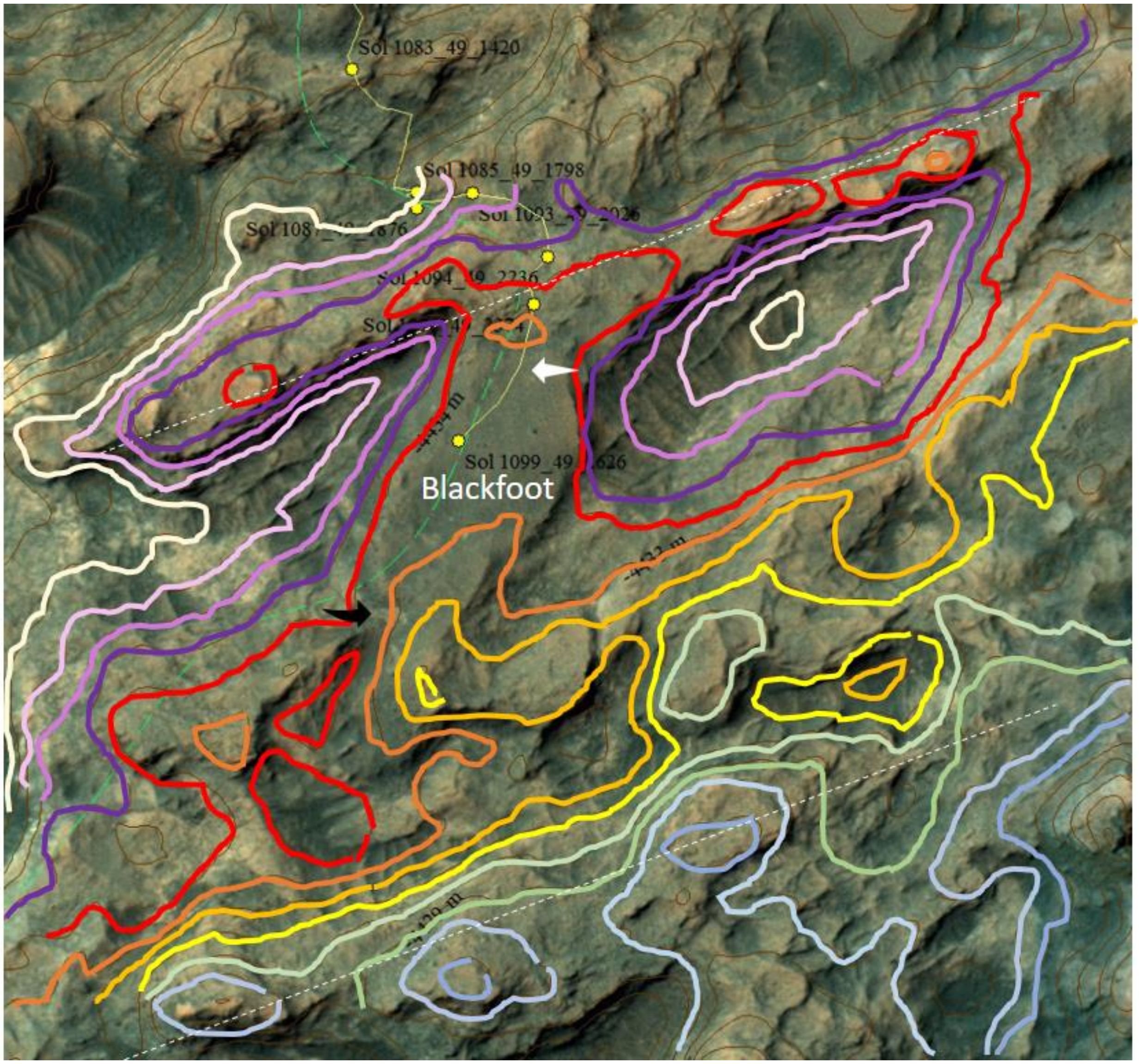

Supplemental Fig. 1-1. HiRISE image mosaic and 1 m elevation contour map (highlighted with brightly colored lines) that shows the Blackfoot deposit to be an erosional remnant that crosses the scour trend of the topography. Blackfoot is the smooth grey-toned deposit (Sol 1099 marks center area). Black and white arrows mark where Blackfoot is overlying the Murray (black) and Stimson (white) formations. Colored contours reveal the parallel ridges (dashed lines) that Blackfoot bridges and the aligned depressions bordering the deposit.

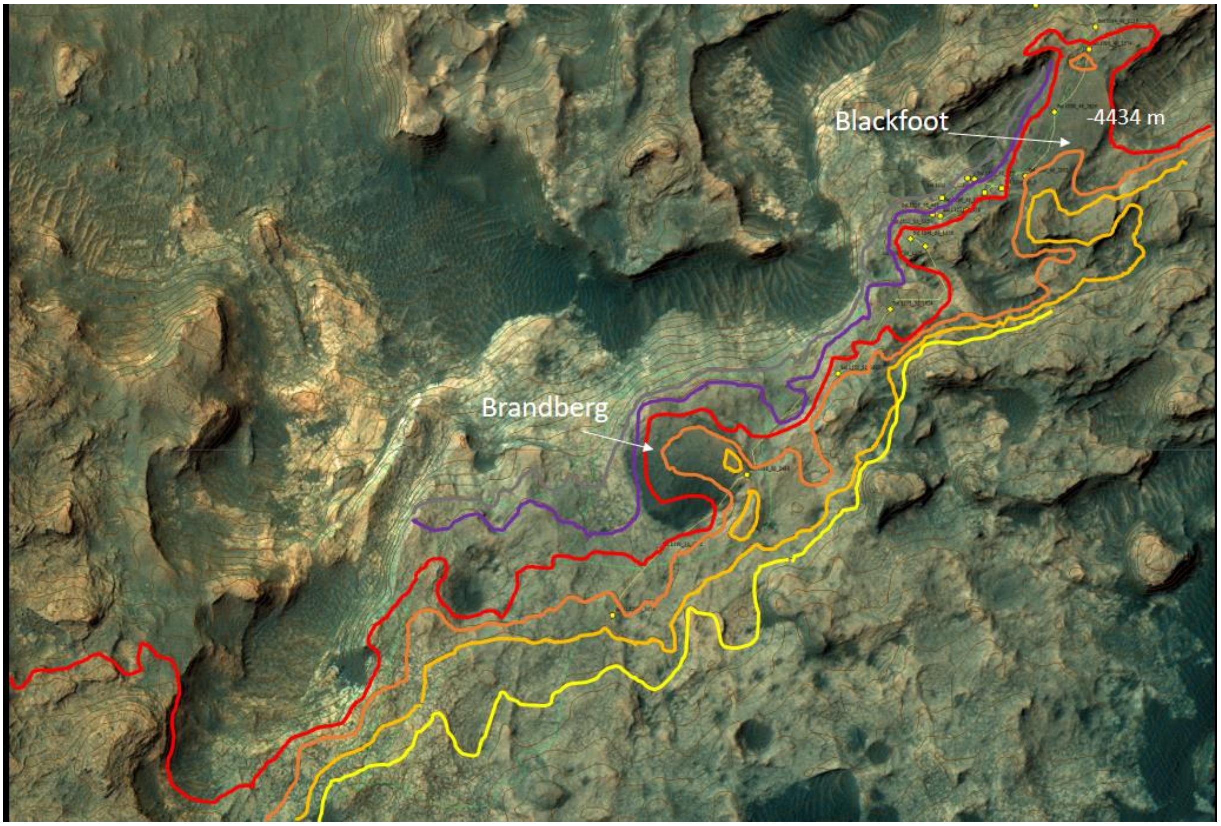

Supplemental Fig. 1-2. HiRISE image mosaic with colored 1 m elevation contours showing the location and elevation of Brandberg relative to Blackfoot.

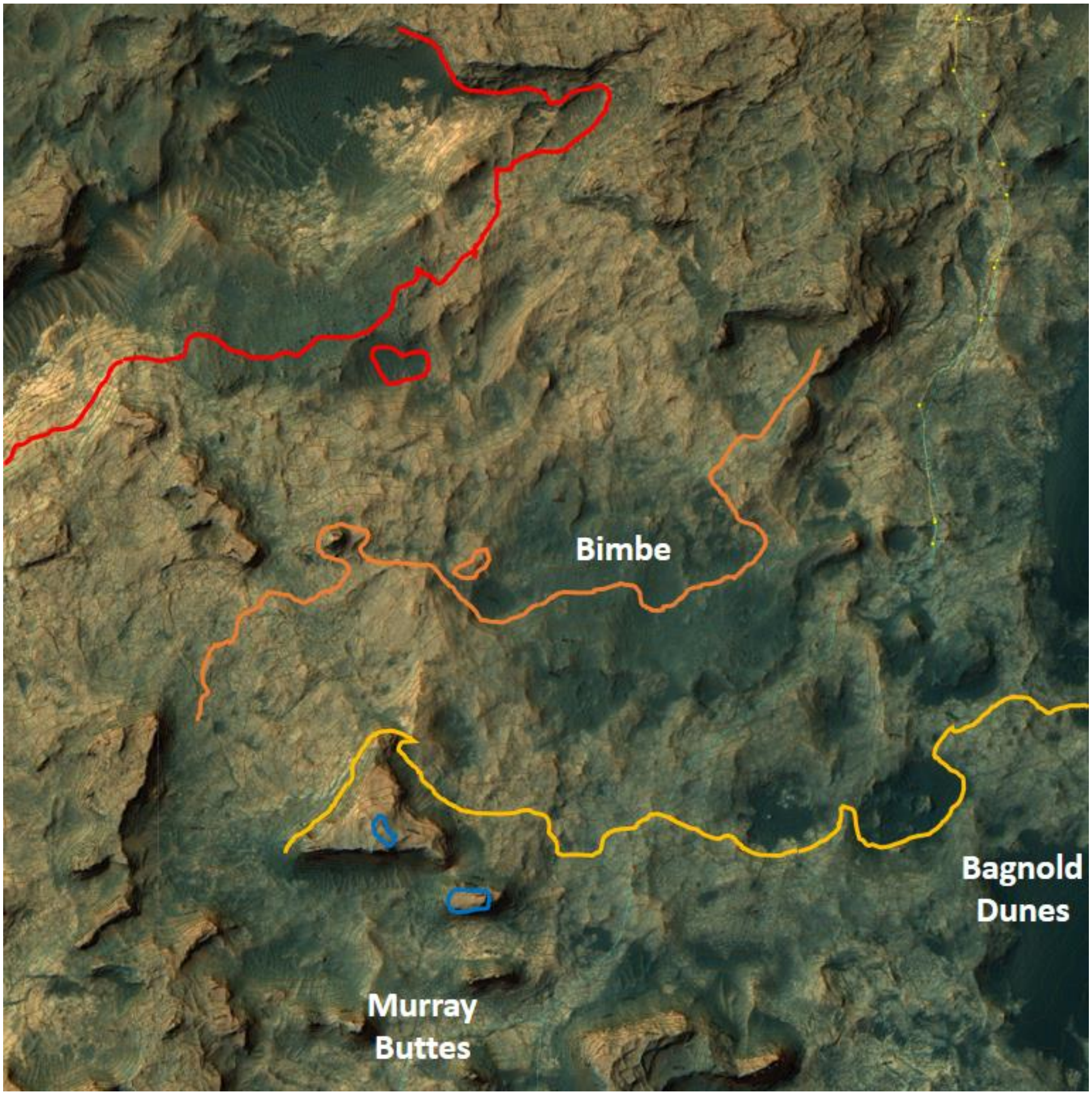

Supplemental Fig. 1-3. HiRISE image with 10 m elevation contour intervals. Orange contour crosses the Bimbe deposit. Red contour crosses a similar deposit to the north.

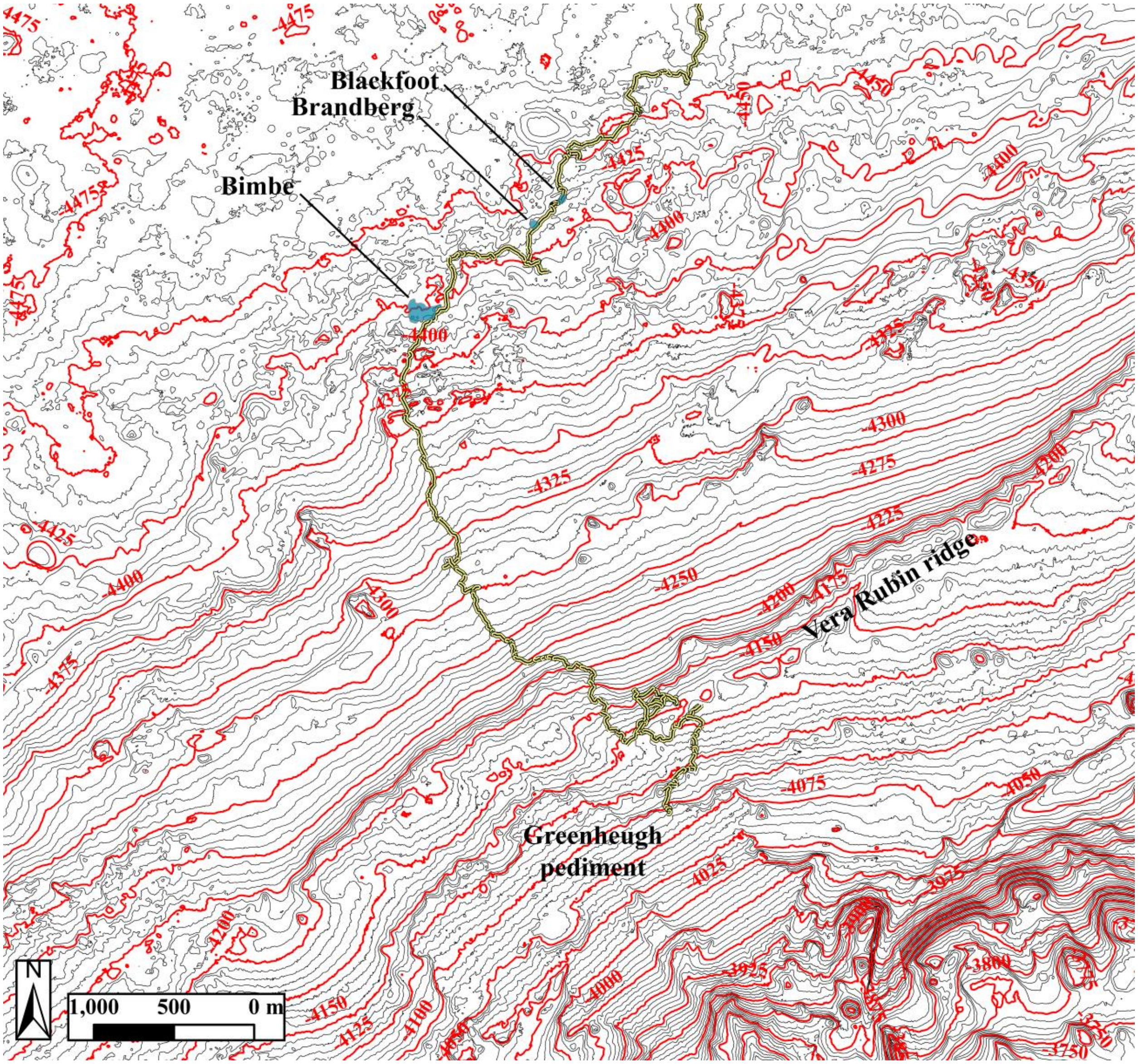

Supplemental Fig. 1-4. Topographic map of a larger area along the rover traverse from the lower edge of Gediz Vallis (lower right center) and Greenheugh pediment to the heterolithic units, showing contour lines at 5 m intervals, and red contour lines at 25 m intervals. Compare with Fig. 1.

2. Description of Particle Size Measurements

Four steps were taken to arrive at a particle size distribution for each heterolithic unit. 1) Mastcam Mosaics that provide extensive close view of particles across a patch were inspected, and then individual photographs were selected for analysis. 2) For each image, a line was drawn across the field of particles. These transects were chosen based on being close to the rover, appearing representative of the deposit, and to be approximately equidistant from the rover at all points. 3) For all stones bigger than three pixels, the major and minor axes of each stone that crossed the transect were measured manually. 4) Conversion from pixels to millimeters was made using Eq. 1 given the Instantaneous Field of View (IFOV) for each camera and a manually-estimated distance from the rover. Measurements of Mastcam-100 (M100) and Mastcam-34 (M34) images were made. The number of stones counted for each heterolithic unit ranged from 150 to 319 (Table 1). The M100 analysis gives a finer size distribution than the M34 dataset. Values used here rely on the more highly resolved M100 data.

$$D_i = 1000(x \tan \alpha) \quad (1)$$

where D = *particle size along dimension i (mm)*
x = *distance from rover (m)*
α = *IFOV for a given camera*

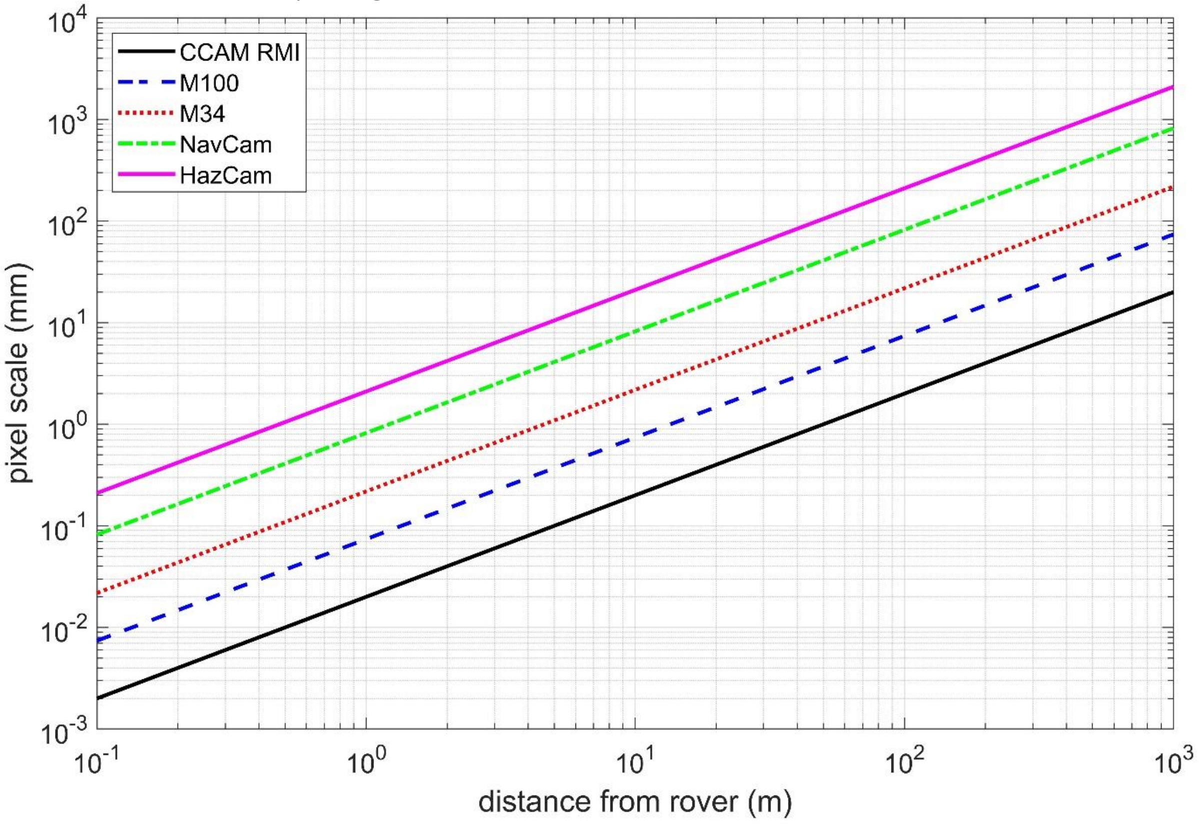

Supplemental Fig. 2-1 IFOV curves for several cameras onboard Curiosity.

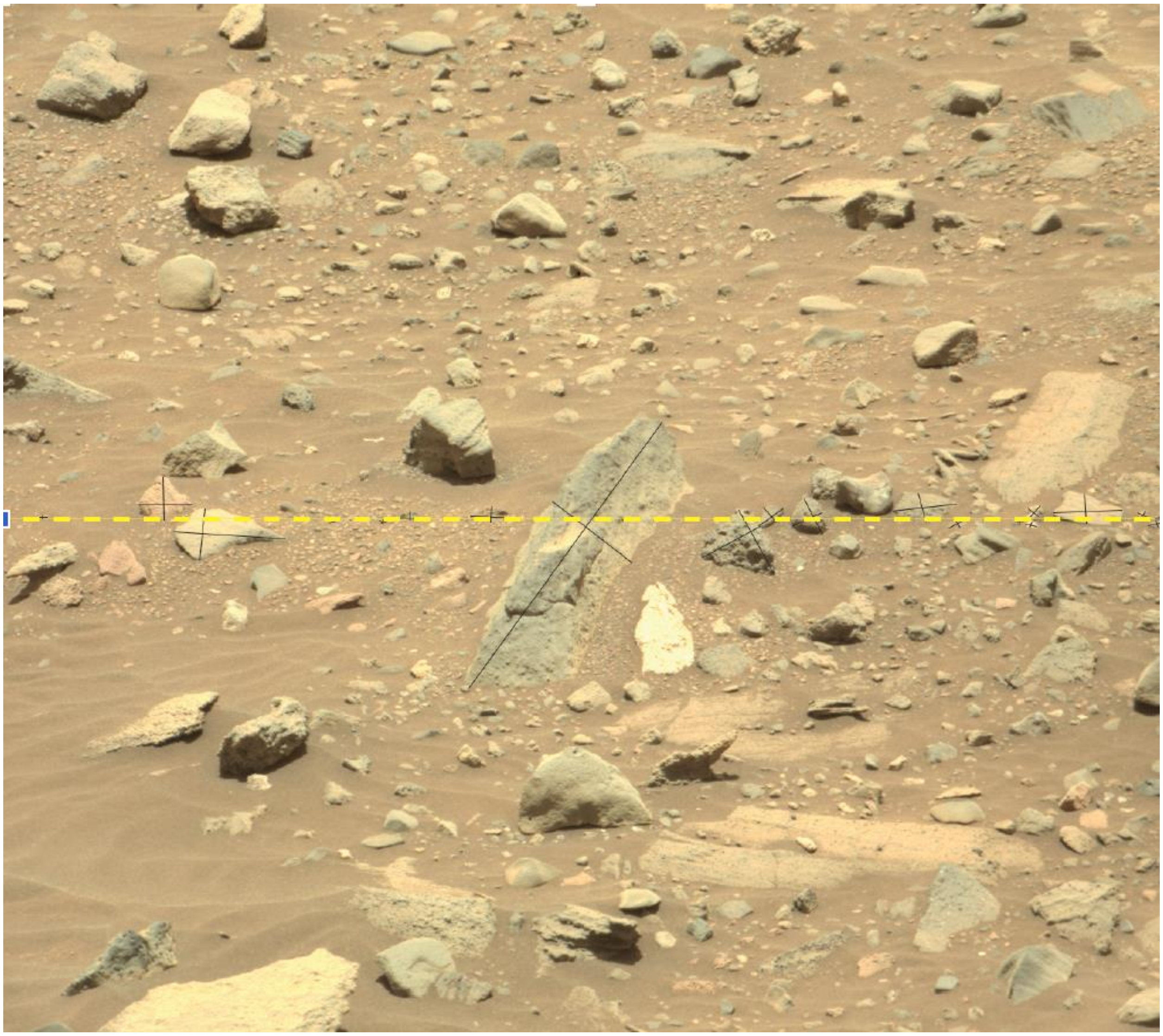

Supplemental Fig. 2-2. Example of measurements made along a transect across the Bimbe deposit.

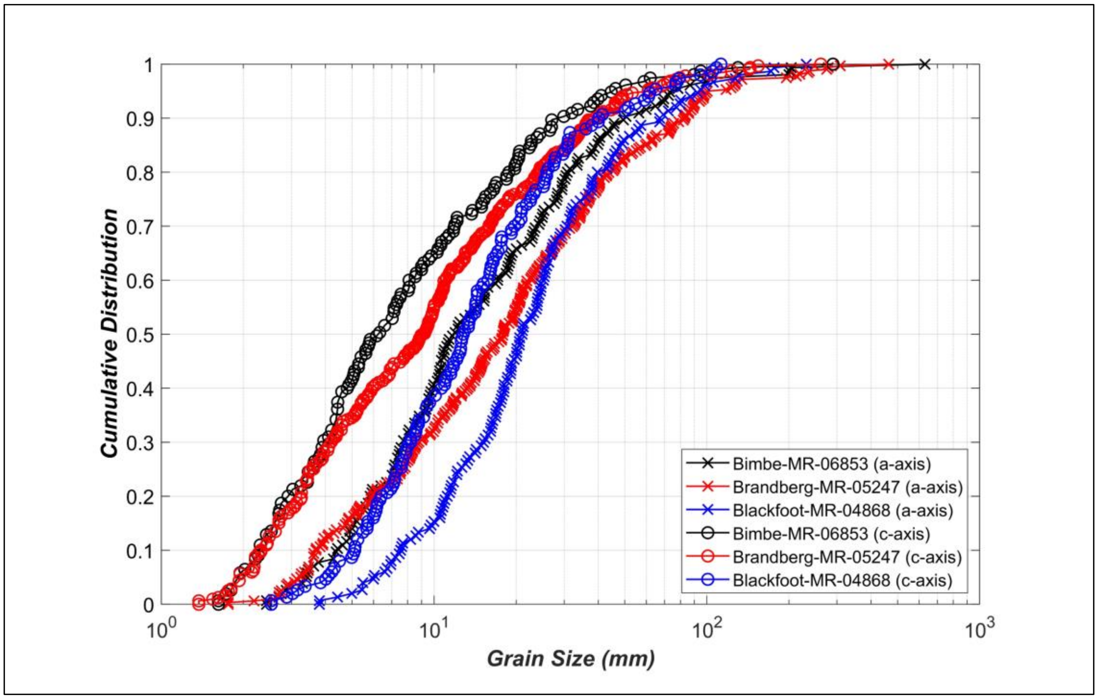

Supplemental Fig. 2-3. Size distribution by short and long axis for the three heterolithic units.

	Bimbe		Brandberg		Blackfoot		Bimbe	Brandberg	Blackfoot
	MR-06853		MR_05247		MR_04868				
	a-axis	c-axis	a-axis	c-axis	a-axis	c-axis	b-axis	b-axis	b-axis
n	155	155	319	319	150	150			
Mean (mm)	27.3	14.6	22.7	17.2	31.6	19.8	21.7	24.3	25.4
D50 (mm)	11.9	6.3	17.9	9.2	21.1	12.9	9.3	13.2	16.0
D84 (mm)	38.3	21.3	55.2	29.6	46.6	30.2	32.9	44.1	39.9
D16 (mm)	5.3	2.7	4.9	2.8	10.5	6.0	3.8	4.3	7.0
Min (mm)	2.4	1.3	1.8	1.4	3.8	2.5	3.0	1.7	3.4
Max (mm)	630.0	290.0	464.6	261.7	231.4	112.6	370.8	409.3	111.8

Table 2-1. Results of particle size analysis. The intermediate axis was calculated by estimating the Corey Shape Factor (CSF) to be 0.6 and solving for the intermediate axis in the definition (CSF = $c/(ab)^{1/2}$).

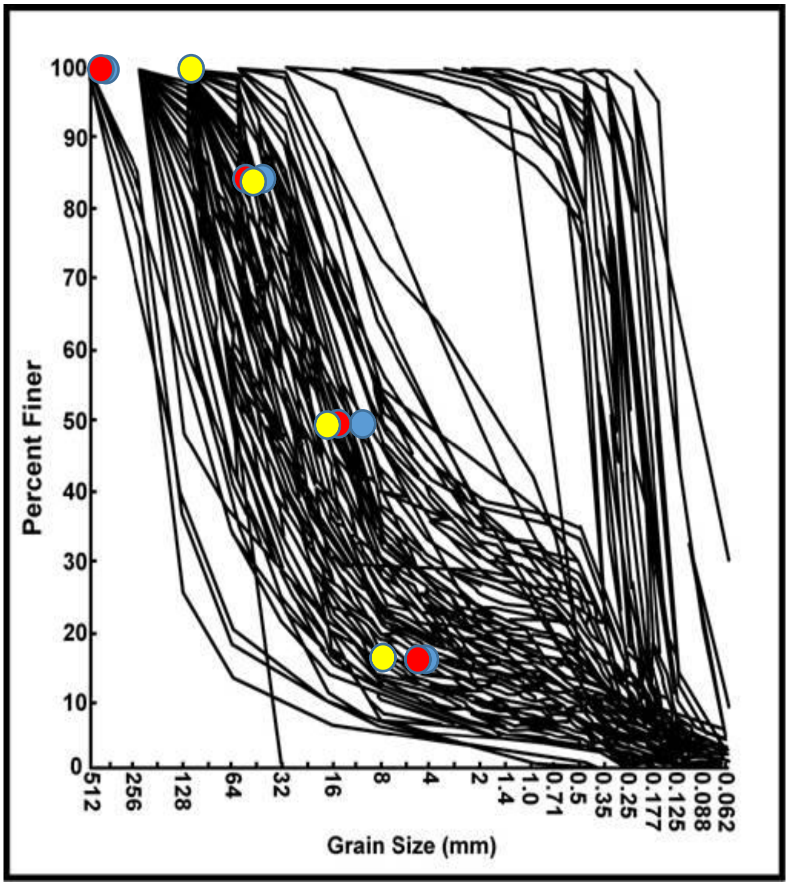

Supplemental Fig. 2-4. Size analysis results for the three heterolithic units, Blackfoot (blue), Brandberg (red), and Bimbe (yellow), compared to 174 river bed deposits analyzed in 12 rivers in Alberta, Canada (reported by Shaw and Kellerhals, 1982). Scattered boulders, not counted in the line transects analyzed, occurred in all three deposits.

3. Description of Reference Points and Contours in Figure 21, and Supporting Figures.

Targets were chosen to represent typical chemistry of the formations (eolian Stimson and lacustrine Murray) and soil in Gale crater. A few targets were chosen to represent each class by picking homogeneous targets without obvious diagenetic textures (nodules, high-silica halos, etc.), within 4 m target distance of the instrument at the time of analysis, and major-element oxide totals close to 100% (except soils), while removing points in bedrock targets that hit Ca-sulfate veins, soil, and relatively rare out-of-focus points. Each plotted reference point is the average composition of the ChemCam observation points.

Murray formation: To represent Hartmann's Valley (HV) Murray, 33 points from the following targets were used: "Andara" (sol 1375), "Okoloti" (sol 1375), "Khorixas" (sol 1375), "Koes" (sol 1380), "Onawa" (sol 1380), "Rundu" (sol 1382), "Aegis_post_1383a," and "Epembe" (sol 1385). These targets represent the area from just above the "Oudam" drill location to a location due east from Bimbe between Bimbe and Helgas Dune. HV Murray is stratigraphically just below Bimbe and serves as a comparison of the Murray bedrock that underlies Bimbe. The soil data point is represented by 20 points from three soils observed throughout the mission up to Sol 1108: "Portage" (Sol 89), "Kings_Peak" (Sol 778), and "Utopia" (Sol 1108).

Stimson formation compositions are based on ChemCam observation points targeting both normal and concretion-rich Stimson facies at the Emerson plateau (sols 990-1154) and Naukluft plateau (sols 1279-1352) localities. ChemCam observation points of the Stimson formation that targeted obvious alteration features such as calcium-sulfate mineral veins or fracture associated halos were excluded from the dataset, as were those outside the 95–105 % total sum of oxide range. Contours for FeO_T, Al_2O_3, MgO, CaO, Na_2O, K_2O, SiO_2 and total alkalis used 331 points for observations at distances < 4 m.

The density contour plots in Fig. 21 show the density distribution of a dataset between x and y composition variables, similar to a 2D histogram. Targets within 4 m of the instrument were used. Due to the small sampling footprint of the ChemCam LIBS laser (350–550 µm for distances of 2–7 m from the rover mast, Maurice et al., 2016), analyses of sedimentary targets with a heterogeneous mineral assemblage are often not representative of whole rock compositions, particularly if the target is coarse-grained (grain diameter >1 mm; Cousin et al., 2017). Density contours generate a better approximation of the bulk composition than the mean as the focus is not skewed by extreme outlying values. Density contours can also highlight subgroups of data with unique compositions aiding in the identification of endmembers within the dataset. This method has proven useful to illustrate the compositional foci and geochemical trends across stratigraphic groups in Gale crater (Bedford et al., 2019), in addition to determining volcanic/magmatic endmember compositions for Gale igneous float and clast (Edwards et al., 2017).

Contours for the ChemCam Stimson formation are generated using the MATLAB `dscatter` algorithm (MathWorks Inc, 2003-2004). Density is calculated by the number of data points within each pixel (bin) of a grid and smoothed according to the methods of Eilers and Goeman (2004). For the Stimson formation dataset we generated the density contours using a bin size of

100 x 100 and a smoothing factor of 20 using a total of 331 ChemCam observation points. Contour lines are defined as the number of smoothed data points within each bin and are based on their level step which is set in this study to $1x10^{-3}$. The average number of smoothed data points per bin represented by the contour can be calculated by multiplying the sample number by the level step and contour number. For example, the fourth contour towards the focus for the Stimson formation will have an average of $331 * 1x10^{-3} * 4 = 1.3$ smoothed data points per bin.

The Stimson formation bedrock has a high density of concretions in certain areas. Overall, the concretions do not show much deviation from bulk rock composition but for FeO_T there are a number of extreme outliers to high iron concentrations, particularly in the Naukluft plateau locality (see the boxplot below). These concretions seem to relate to preferential cementation of the sandstone. If this is true, the sandstone of the Stimson formation is predominately iron-oxide forming from olivine diagenesis when Stimson formation precursor material was buried (e.g., Hausrath et al., 2018). So if the nodular Bimbe is derived from overlying Stimson then it would be possible that it could have Fe-rich concretions based on what has been analyzed and interpreted for the concretions at the Emerson and Naukluft plateaus. If these features don't distort any sedimentary structures (like laminations) then they are more likely to be concretions.

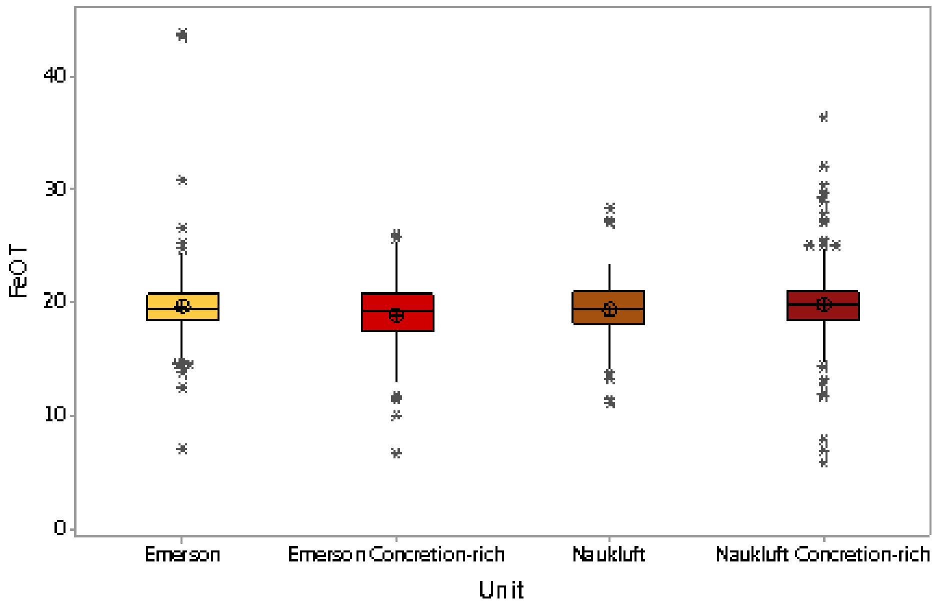

Supplemental Fig. 3-1. Boxplot showing distribution of FeO_T in different populations of Stimson formation targets. Circles with crosses indicate the means; asterisk symbols indicate outliers.

The boxplot figures below are provided to supplement Fig. 21. Circles with crosses indicate the means; asterisk symbols indicate outliers. L = layered; M = massive.

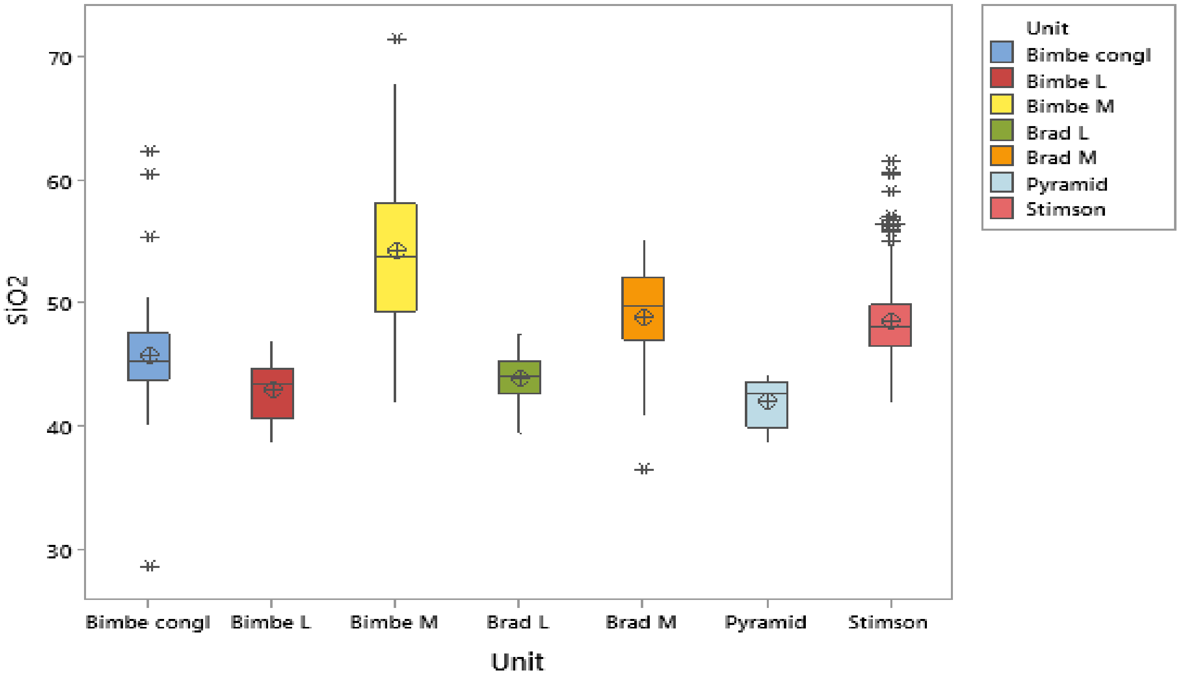

Supplemental Fig. 3-2. Boxplot of SiO_2. See details above.

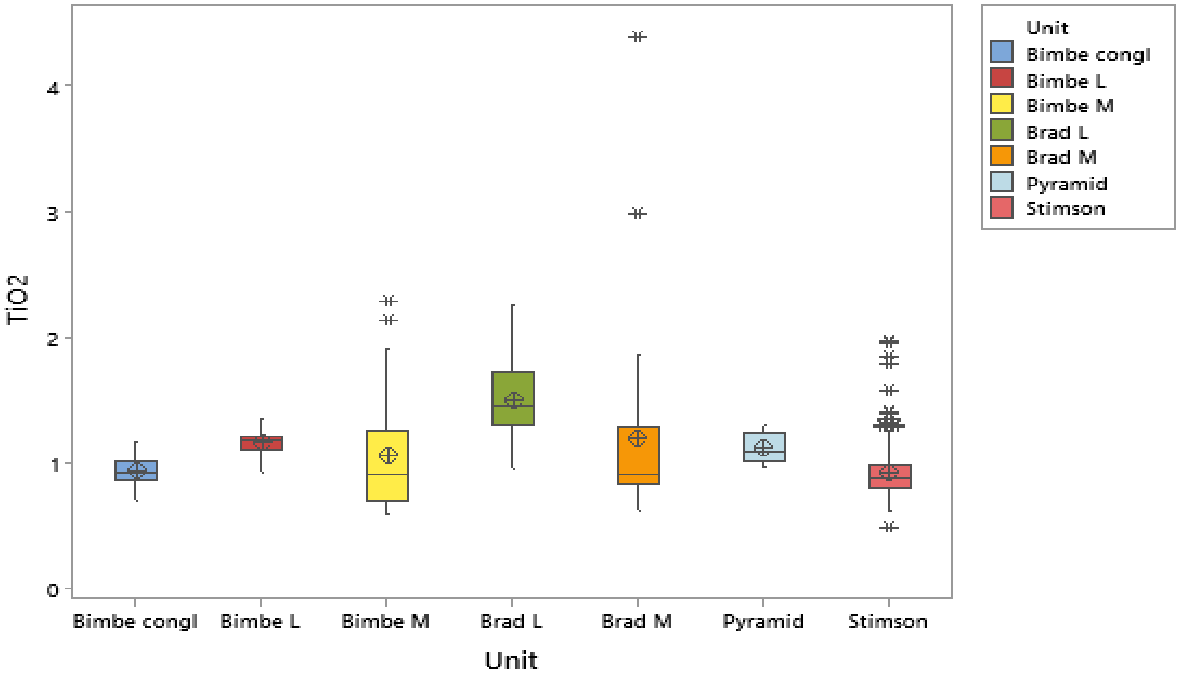

Supplemental Fig. 3-3. Boxplot of TiO_2. See details above.

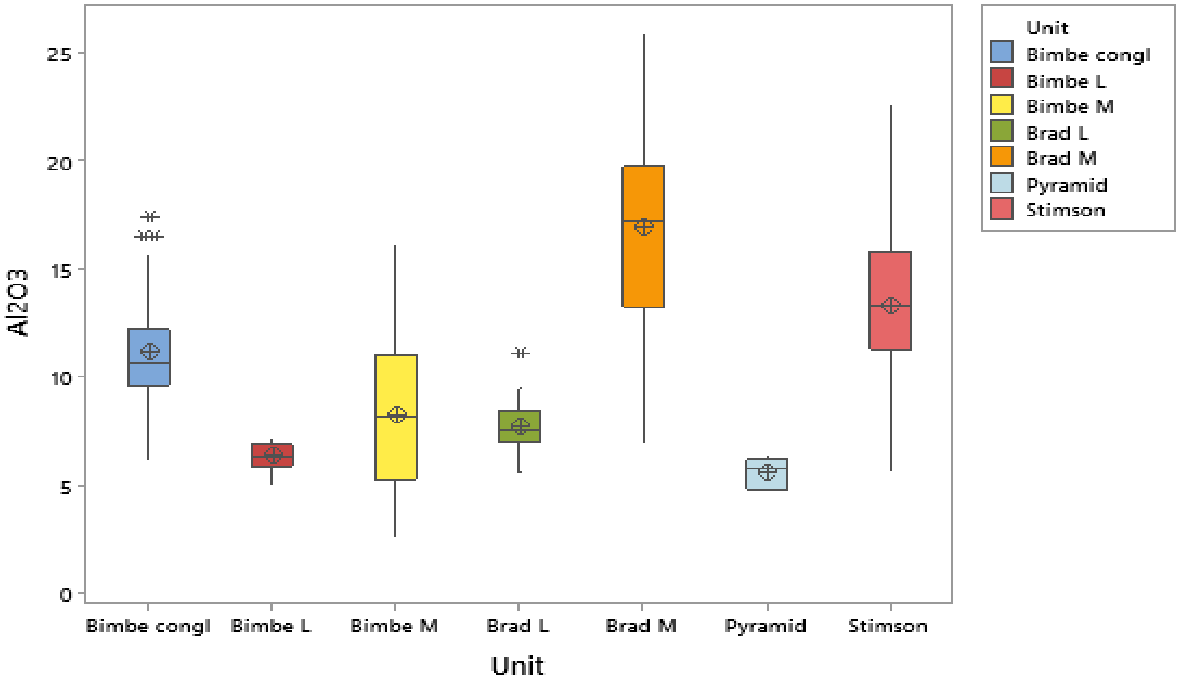

Supplemental Fig. 3-4. Boxplot of Al_2O_3. See details above.

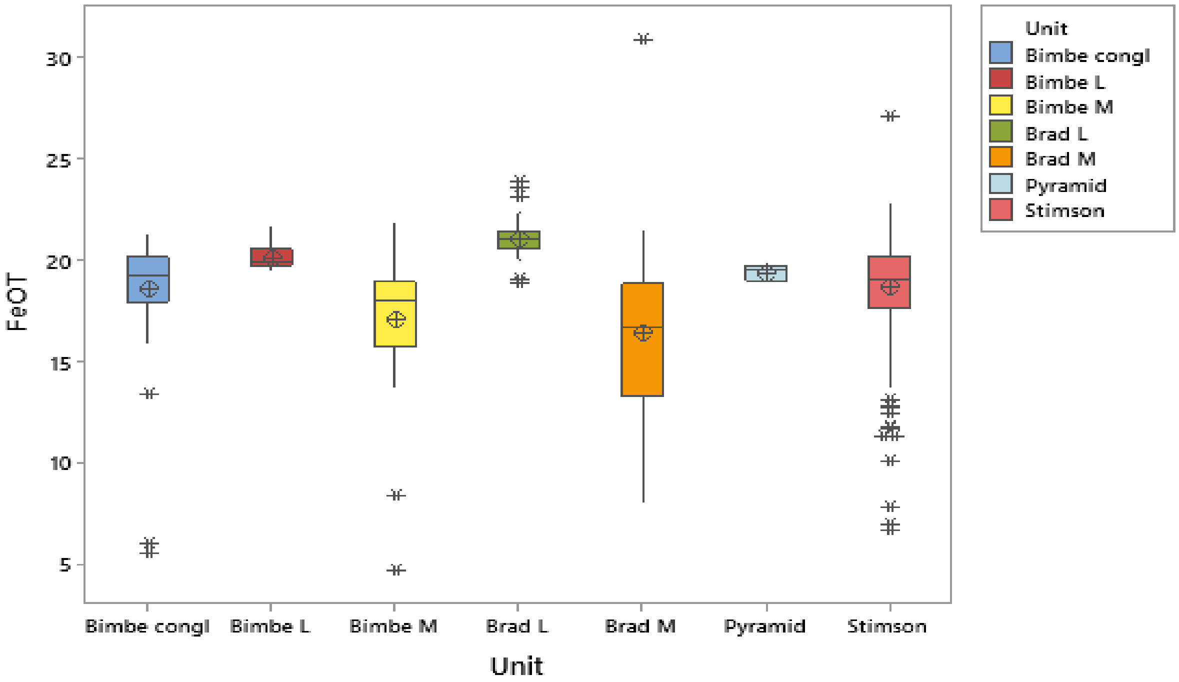

Supplemental Fig. 3-5. Boxplot of FeO_T. See details above.

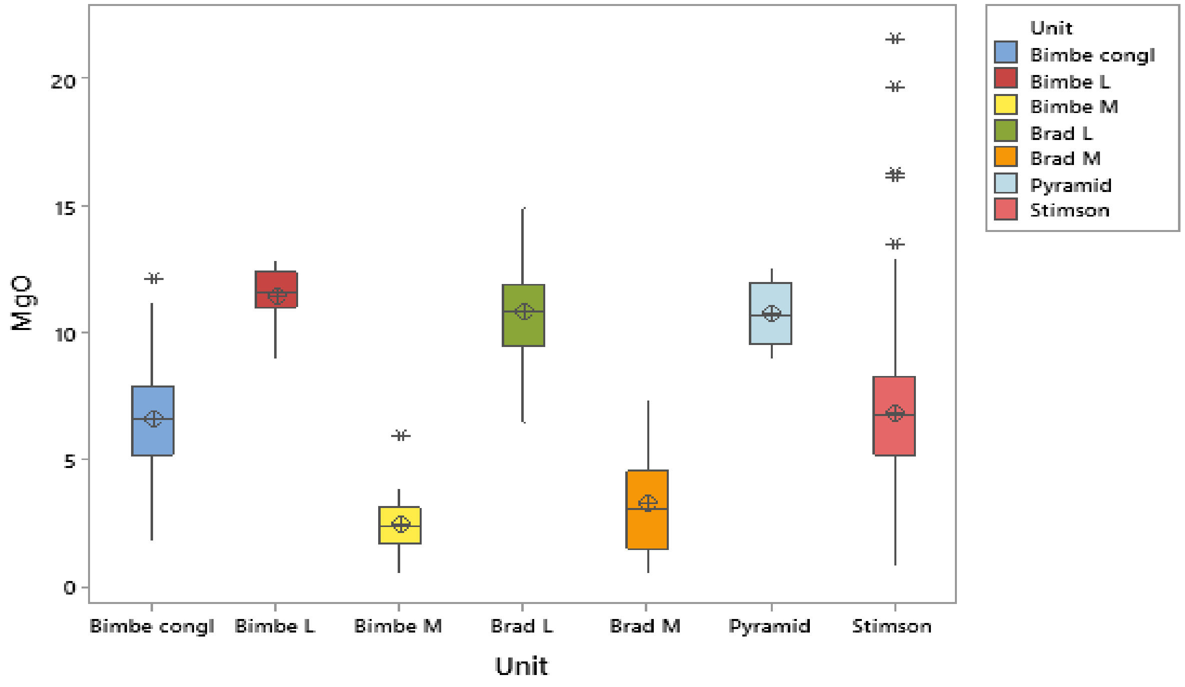

Supplemental Fig. 3-6. Boxplot of MgO. See details above.

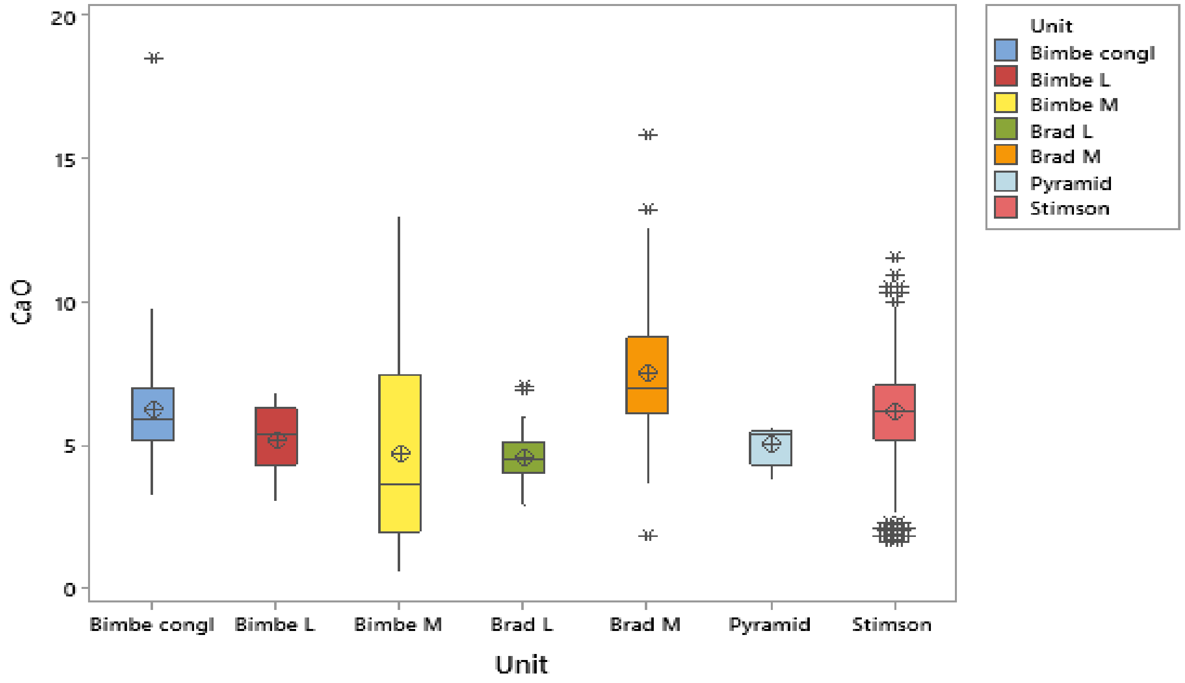

Supplemental Fig. 3-7. Boxplot of CaO. See details above.

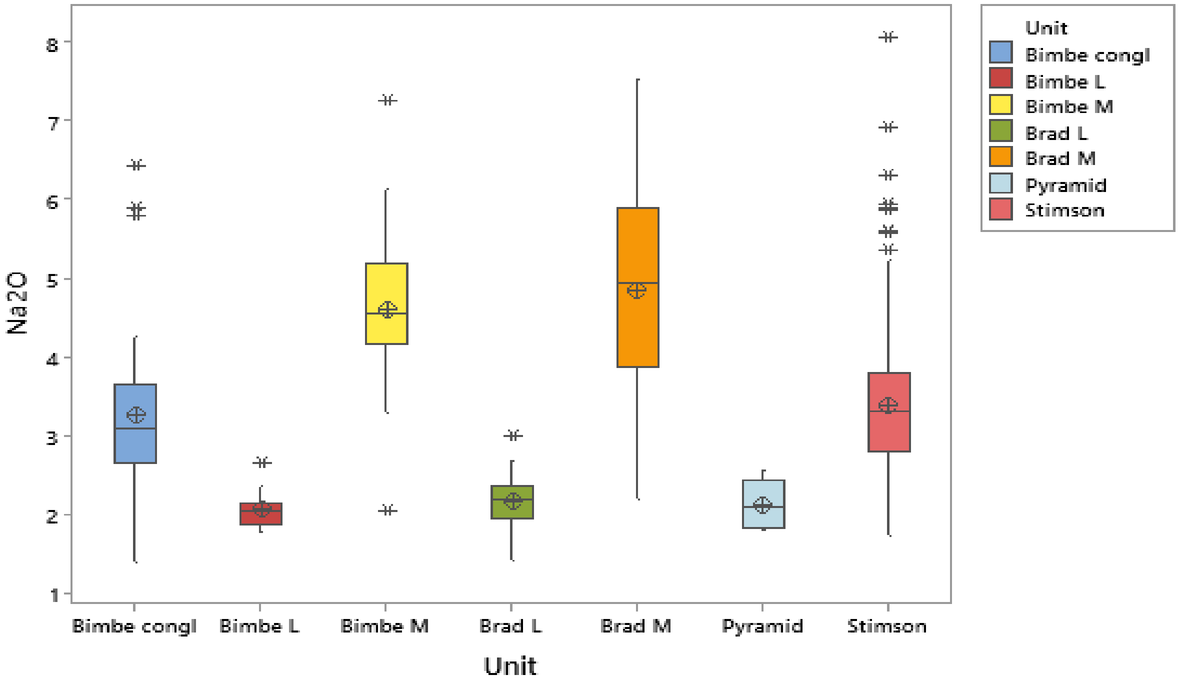

Supplemental Fig. 3-8. Boxplot of Na_2O. See details above.

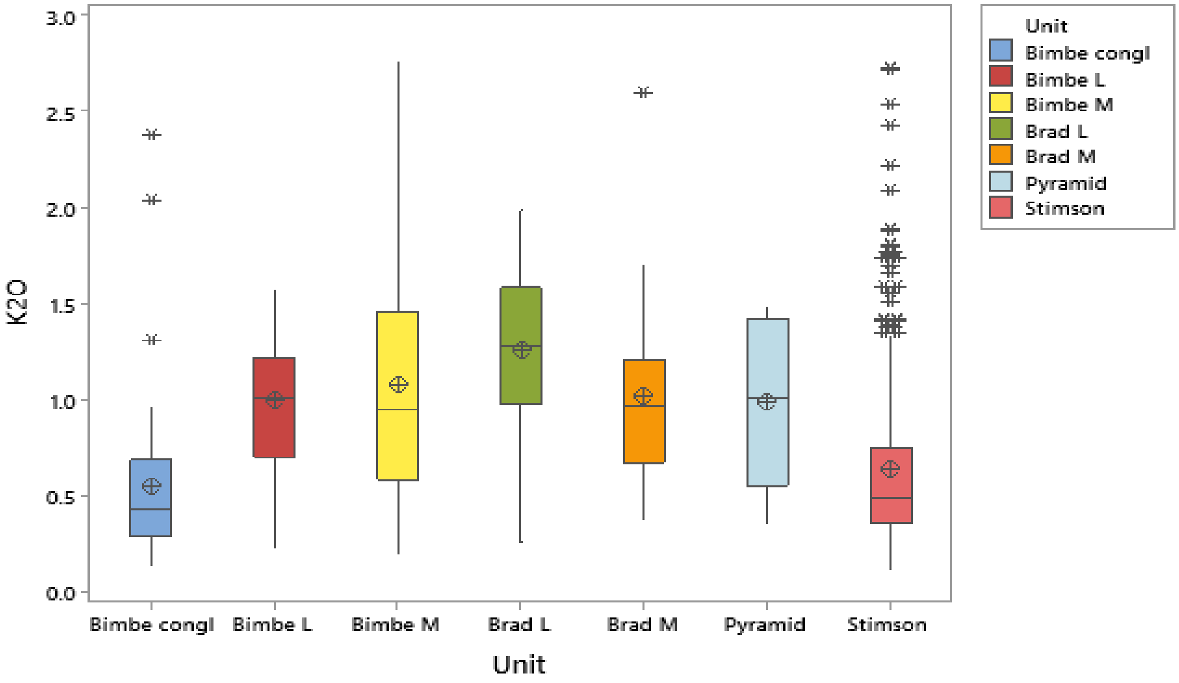

Supplemental Fig. 3-9. Boxplot of K_2O. See details above.

4. Description and Results of Equivalence Tests

Two-sample equivalence tests were conducted using Minitab v17 for each major element oxide (SiO_2, TiO_2, Al_2O_3, FeO_T, MgO, CaO, Na_2O, and K_2O) that defines the datasets. More details are given in Appendix D of Bedford et al. (2019). The precision of the measurements were based on the standard deviations of the Sheepbed Mudstones, a large ensemble of measurements on a fine-grained homogeneous bedrock at Yellowknife Bay (Mangold et al. 2015). Equivalence tests were carried out between Bimbe Massive and Bradbury Massive sandstone targets, Bimbe Layered and Bradbury Layered targets, and Bimbe conglomerates and Stimson sandstones. The results are given below. Although Fig. 21 and the boxplots in the previous section indicate significant similarities between the two layered groups and between Bimbe conglomerates and Stimson, the equivalence test does not give a unanimously positive response. In the case of the layered groups, care must be exercised due to the small sample size for Bimbe.

Table 4-1, Bimbe conglomerates and Stimson (reference).

	Diff of means	SE of diff	EI	Difference ≤ -EI T-value	Difference ≤ -EI P-value	Difference ≥ -EI T-Value	Difference ≥ -EI P-value	Equiv (Y/N)
SiO_2	-2.79	0.69	±3.30	0.74	0.23	- 8.85	0.00	N
TiO_2	-0.01	0.02	±0.20	12.15	0.00	-10.59	0.00	Y
Al_2O_3	-2.21	0.39	±0.90	-3.37	1.00	-8.00	0.00	N
FeO_T	-0.11	0.43	±1.20	2.55	0.01	-3.07	0.00	Y
MgO	-0.28	0.32	±1.40	3.48	0.00	-5.22	0.00	Y
CaO	-0.09	0.32	±1.00	3.44	0.00	-2.89	0.00	Y
Na_2O	-0.12	0.13	±0.30	1.40	0.08	-3.16	0.00	N
K_2O	-0.09	0.06	±0.20	1.86	0.03	-4.61	0.00	Y

Table 4-2, Bimbe massive and Bradbury massive sandstone targets (reference).

	Diff of means	SE of diff	EI	Difference ≤ -EI T-value	Difference ≤ -EI P-value	Difference ≥ -EI T-Value	Difference ≥ -EI P-value	Equiv (Y/N)
SiO_2	5.54	1.23	±3.30	7.19	0.00	1.82	0.96	N
TiO_2	-0.13	0.15	±0.20	0.47	0.32	-2.25	0.02	N
Al_2O_3	-8.76	1.02	±0.90	-7.73	1.00	-9.50	0.00	N
FeO_T	-0.67	0.94	±1.20	2.00	0.03	-0.57	0.29	N
MgO	-0.81	0.34	±1.40	1.75	0.04	-6.49	0.00	Y
CaO	-2.83	0.69	±1.00	-2.63	1.00	-5.52	0.00	N
Na_2O	-0.24	0.27	±0.30	0.23	0.41	-1.97	0.03	N
K_2O	0.06	0.11	±0.20	2.27	0.01	-1.25	0.11	N

Table 4-3, Bimbe layered and Bradbury layered targets (reference).

	Diff of means	SE of diff	EI	Difference ≤ -EI T-value	Difference ≤ -EI P-value	Difference ≥ -EI T-Value	Difference ≥ -EI P-value	Equiv (Y/N)
SiO_2	-0.98	0.64	±3.30	3.62	0.00	-6.67	0.00	Y
TiO_2	-0.33	0.04	±0.20	-3.21	1.00	-12.87	0.00	N
Al_2O_3	-1.35	0.18	±0.90	-2.51	0.99	-12.63	0.00	N
FeO_T	-0.92	0.17	±1.20	1.61	0.06	-12.36	0.00	N
MgO	-0.64	0.35	±1.40	5.87	0.00	-2.17	0.02	Y
CaO	0.61	0.32	±1.00	5.06	0.00	-1.23	0.12	N
Na_2O	-0.11	0.07	±0.30	2.68	0.01	-5.94	0.00	Y
K_2O	-0.26	0.10	±0.20	-0.58	0.72	-4.46	0.00	N

5. Some Unique Compositional Features of the Bimbe Float Rocks

ChemCam observation point #3 of the conglomerate Balombo revealed one of the stronger fluorine peaks observed by ChemCam, calibrating to ~1.5 wt. % F. This corresponded to a relatively strong increase in calcium, to ~18 wt. % CaO, suggesting the presence of a small amount of CaF_2 in this observation. Among the 30 spectra obtained on this observation point (one per laser pulse), the compositions remain steady, indicating that the Ca-F enrichment region is deeper than the laser pit. The pit itself appears light toned. Fluorine has been observed in other locations in Gale, including in soils and sediments as well as in igneous float rocks. It was often observed associated with Al and P, suggesting fluorapatite, but other times associated only with Ca, suggesting fluorite (Forni et al., 2015; Cousin et al., 2017; Nachon et al., 2016).

6. The Heights of the Murray Buttes

Measurements were made to estimate the heights of the Murray Buttes using the MSL WebGIS. The topography is from Parker and Calef (2016), derived from HiRISE stereo pair DEMs registered to MGS MOLA elevations. An example plot below shows how the measurements were made, along with a table of the results. The butte designations are those adopted by the MSL team. The rover path is also shown, from north (top), past Bimbe, and to the south. A key issue is that all of the Murray Buttes occur on a slope; thus, the terrain north of each butte is lower than the terrain south of each butte. This means that the maximum height of a given butte is really from the north side to the highest point on the butte top. Instead of doing just a "maximum" height, a minimum and maximum were estimated. Overall, with one exception, these range from about 7 m (minimum) to 17 m (maximum) with an average of 8 m (min) to12 m (max).

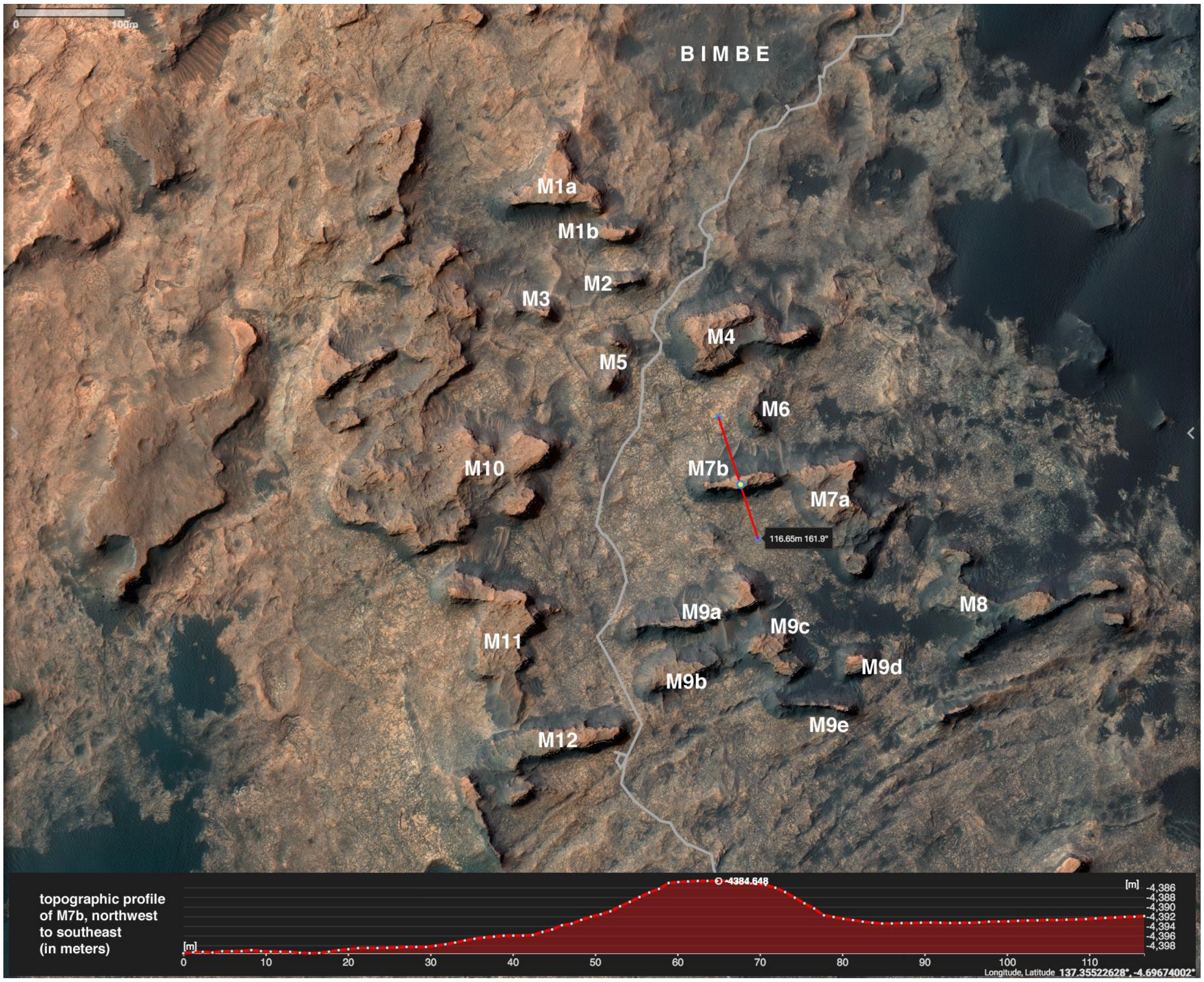

Butte name	highest elevation on butte (m)	low elevation on one side (m)	low el. on other side (m)	max height (m)	min height (m)
**M1a**	-4404	-4419	-4416	**15**	**12**
**M1b**	-4402	-4410	-4409	**8**	**7**
**M2**	-4400	-4408	-4409	**8**	**9**
**M3**	-4400	-4412	-4408	**12**	**8**
**M4**	-4392	-4407	-4402	**15**	**10**
**M5**	-4395	-4407	-4402	**12**	**7**
**M6**	-4389	-4398	-4398	**9**	**9**
**M7a**	-4379.4	-4396.7	-4383.8	**17.3**	**4.4**
**M7b**	-4384.6	-4398.5	-4393.4	**13.9**	**8.8**
**M8**	-4369.7	-4385.4	-4374.7	**15.7**	**5.0**
**M9a**	-4376.6	-4388.0	-4386.0	**11.4**	**9.4**
**M9b**	-4374.4	-4386.2	-4381.2	**11.8**	**6.8**
**M9c**	-4373.4	-4385.5	-4381.8	**12.1**	**8.4**
**M9d**	-4370.6	-4380.7	-4377.3	**10.1**	**6.7**
**M9e**	-4369.7	-4378.2	-4377.2	**8.5**	**7.5**
**M10**	-4389.4	-4406.2	-4395.9	**16.8**	**6.5**
**M11**	-4380.0	-4394.0	-4388.3	**14.0**	**8.3**
**M12**	-4370.5	-4383.5	-4379.5	**13.0**	**9.0**
			Averages	**12.4**	**7.9**

7. Additional References for Supplemental Section

Bedford C.C., Bridges J.C., Schwenzer S.P., Wiens R.C., Rampe E., Frydenvang J., and Gasda P.J., 2019. Alteration trends and geochemical source region characteristics preserved in the fluviolacustrine sedimentary record of Gale crater, Mars. Geochim. Cosmochim. Acta 246, 234-266, doi:10.1016/j.gca.2018.11.031.

Edwards P.H., et al., 2017. Basalt-trachybasalt samples from Gale crater, Mars. Met. Planet. Sci. 52, 2031-2410, doi:10.1111/maps.12953.

Eilers P.H.C., and Goeman J.J., 2004. Enhancing scatterplots with smoothed densities: Bioinformatics 20(5), 623-628, doi:10.1016/j.sab.2013.05.003.

Forni O., et al., 2013. Independent component analysis classification of laser induced breakdown spectroscopy spectra. Spectrochim. Acta B 86, 31–41.

Shaw J. and Kellerhals R., 1982. The composition of recent alluvial gravels in Alberta river beds, Alberta Research Council Bulletin 41, 151 pp., https://ags.aer.ca/publications/BUL_041.html.

8. Compositions of Individual ChemCam Observation Points, Bimbe

Also included are accuracies and standard deviations of the individual spectra (25) within each observation point. Observation points are arranged consecutively for each target.

Target	SiO2	acc	stdev	TiO2	acc	stdev	Al2O3	acc	stdev	FeOT	acc	stdev	MgO	acc	stdev	CaO	acc	stdev	Na2O	acc	stdev	K2O	acc	stdev	Total
Auchab	45.9	5	0.8	1.04	0.5	0.03	15.2	3.6	0.5	24.1	4.5	1.3	3.9	1.8	0.1	5.3	1.8	0.3	4.24	0.74	0.1	2.81	1.17	0.08	102.47
Auchab	43.8	5.1	0.5	1.02	0.5	0.04	11.7	3.6	0.6	28.2	4.7	1.1	8.8	2.4	0.4	3.9	1.4	0.2	3.17	0.63	0.17	1.54	1.09	0.08	102.09
Auchab	41.6	5.3	0.5	0.91	0.47	0.02	9.2	3.5	0.3	29.7	4.8	0.7	7.7	2.2	0.4	4.5	1.6	0.4	2.81	0.62	0.07	1.73	1.14	0.11	98.17
Auchab	41.6	5.3	0.7	0.87	0.47	0.04	9.8	3.5	0.5	28	4.7	1.3	7.5	2.2	0.3	5.6	1.9	0.5	2.98	0.62	0.14	1.59	1.11	0.13	98.08
Auchab	44.7	5	0.6	0.89	0.47	0.04	11	3.5	0.4	22.1	4.3	0.8	8.1	2.3	0.4	5.7	1.9	0.5	2.97	0.62	0.11	1.43	1.06	0.08	96.92
aegis_post_1400a	45.3	5	0.7	0.84	0.46	0.02	11.3	3.5	0.7	19.2	4	0.5	6.9	2.1	0.2	5.9	1.9	0.5	3.65	0.67	0.19	0.67	0.72	0.03	93.73
aegis_post_1400a	44.8	5	0.3	0.84	0.46	0.02	8.3	3.4	0.2	20.8	4.2	0.2	10.3	2.6	0.8	4.1	1.5	0.9	2.64	0.62	0.09	0.65	0.71	0.1	92.33
aegis_post_1400a	42	5.2	0.5	1.02	0.5	0.04	10.1	3.5	0.3	19.9	4.1	0.2	5.5	1.9	0.1	5.1	1.7	0.4	3.15	0.63	0.2	0.63	0.7	0.06	87.46
aegis_post_1400a	48.1	4.9	1.2	0.82	0.45	0.03	15.5	3.6	2.5	17.8	3.8	1	5.5	1.9	0.7	6	2	0.5	3.48	0.65	0.33	0.41	0.61	0.03	97.7
aegis_post_1400a	45	5	0.5	0.81	0.45	0.02	10	3.5	0.6	18.8	3.9	0.4	8.8	2.4	0.3	6.7	2.2	0.3	3.05	0.63	0.15	0.38	0.6	0.03	93.67
aegis_post_1400a	46.7	4.9	0.3	0.87	0.47	0.03	14.8	3.6	1.4	18.8	3.9	0.3	6	2	0.3	5.9	2	0.2	3.35	0.64	0.15	0.5	0.65	0.02	96.98
aegis_post_1400a	44.7	5	0.5	0.7	0.42	0.01	7.5	3.4	0.3	18	3.8	0.5	12.1	2.9	0.5	6.8	2.2	0.4	2.12	0.63	0.09	0.48	0.64	0.03	92.39
aegis_post_1400a	45.5	5	0.7	0.94	0.48	0.07	13.6	3.6	1.5	19.7	4	0.7	6.5	2.1	0.4	5.3	1.8	0.6	3.3	0.64	0.28	0.49	0.64	0.06	95.27
aegis_post_1400a	45.4	5	0.3	0.9	0.47	0.02	11.9	3.6	0.2	18.6	3.9	0.3	8	2.3	0.6	5.3	1.8	0.4	3.5	0.65	0.16	0.7	0.73	0.03	94.33
Aussenkehr	48.5	4.9	1.2	1.46	0.61	0.09	5.3	3.1	0.5	20.3	4.1	1.1	2.1	1.6	0.1	4.6	1.6	1.2	4.38	0.76	0.21	0.93	0.84	0.19	87.56
Aussenkehr	51.8	5	1.7	0.63	0.4	0.06	8.2	3.4	0.8	19	3.9	1.4	2.1	1.6	0.3	2.6	1.1	0.7	3.47	0.65	0.24	0.83	0.79	0.1	88.46
Aussenkehr	54.6	5.1	1.9	1.11	0.52	0.12	9.4	3.5	0.7	15.1	3.4	0.9	1.4	1.5	0.1	3.4	1.3	0.5	5.45	0.93	0.42	0.96	0.85	0.17	91.42
Aussenkehr	42.1	5.2	0.6	0.69	0.42	0.02	2.7	2.8	0.3	19.4	4	0.2	3.5	1.7	0.1	12.9	3.2	0.6	2.05	0.63	0.1	0.2	0.54	0.06	83.65
Aussenkehr	56.1	5.2	1.7	1.01	0.5	0.08	11.6	3.5	0.5	18.9	3.9	0.6	1.3	1.5	0.2	3.6	1.3	0.6	4.61	0.8	0.36	1.71	1.14	0.16	98.81
Aussenkehr	48.6	4.9	0.7	1.8	0.7	0.39	8.2	3.4	0.8	15.8	3.5	0.7	2.1	1.6	0.2	6.9	2.2	1	4.27	0.74	0.15	1.21	0.97	0.11	88.91
Aussenkehr	55.8	5.2	1.4	0.63	0.4	0.03	9.6	3.5	0.8	15.3	3.4	1.7	1.4	1.5	0.2	7.7	2.4	0.6	5.47	0.93	0.43	1.61	1.11	0.26	97.54
Aussenkehr	56.2	5.2	1.2	0.6	0.39	0.04	11.7	3.6	0.8	18.5	3.9	0.8	1.4	1.5	0.1	2.4	1.1	0.5	4.54	0.79	0.26	1.75	1.15	0.2	97.13
Aussenkehr	52.2	5	0.5	1.91	0.73	0.22	6.1	3.2	0.3	16.6	3.6	0.9	1.7	1.6	0.1	1.6	1	0.2	4.83	0.84	0.12	1.12	0.93	0.1	86.1
Aussenkehr	49.4	4.9	1.1	0.79	0.45	0.08	8.8	3.5	0.9	16.3	3.6	1.6	1.7	1.6	0.4	5.7	1.9	0.7	4.16	0.72	0.23	0.93	0.84	0.24	87.84
Canico	40	5.5	0.8	1.05	0.5	0.04	8.1	3.4	0.4	23	4.4	1	5	1.9	0.2	5.1	1.7	0.6	2.54	0.62	0.08	0.66	0.71	0.06	85.47
Canico	36.4	6	0.5	1.11	0.52	0.04	8.7	3.5	0.3	28.5	4.7	0.8	5	1.9	0.2	3	1.2	0.4	2.73	0.62	0.1	0.86	0.81	0.06	86.38
Canico	44.7	5	0.5	0.78	0.44	0.02	8.1	3.4	0.2	19.7	4	0.4	7.3	2.2	0.2	5	1.7	0.5	2.75	0.62	0.11	0.73	0.75	0.05	89
Canico	38.5	5.7	0.4	1	0.49	0.04	8	3.4	0.2	24.6	4.5	1.1	5.1	1.9	0.1	6.8	2.2	0.9	2.54	0.62	0.1	0.76	0.76	0.05	87.45
Canico	52	5	0.4	0.81	0.45	0.01	17.3	3.7	0.4	13.5	3.2	0.5	2.1	1.6	0.1	6.4	2.1	0.4	5.77	0.96	0.16	0.73	0.75	0.04	98.66
Chinchimane	40.4	5.4	0.5	1.32	0.57	0.06	6	3.2	0.6	20.3	4.1	0.2	12.4	3	0.7	4.3	1.5	0.4	2.01	0.63	0.1	1.15	0.94	0.11	87.95
Chinchimane	41.8	5.2	0.8	1.18	0.53	0.05	6.4	3.2	0.5	19.7	4	0.2	11.3	2.8	0.4	4.9	1.7	0.4	2.14	0.62	0.09	1.56	1.1	0.11	88.99
Chinchimane	40.4	5.4	0.6	1.1	0.52	0.05	5.9	3.2	0.3	20.6	4.1	0.3	12	2.9	0.8	5.5	1.8	0.6	1.78	0.64	0.09	0.98	0.86	0.11	88.27
Chinchimane	38.8	5.6	0.6	1.2	0.54	0.06	5.9	3.2	0.3	20.5	4.1	0.6	12.6	3	1.2	4.5	1.6	0.7	1.77	0.64	0.08	0.99	0.87	0.13	86.3
Chinchimane	40.7	5.4	0.4	1.05	0.5	0.06	6.2	3.2	0.4	19.9	4.1	0.3	10.4	2.7	0.9	6.8	2.2	0.6	2.03	0.63	0.12	1.01	0.88	0.13	88.02
aegis_post_1406a	56.4	5.2	0.7	0.64	0.4	0.01	4.5	3	0.3	18.4	3.9	0.6	3	1.7	0.1	3.3	1.3	0.2	5.46	0.93	0.18	0.7	0.73	0.06	92.42
aegis_post_1406a	58.4	5.2	1.5	0.98	0.49	0.04	4.6	3	0.6	20.9	4.2	1.7	2.5	1.6	0.1	3.7	1.4	0.3	7.27	1.04	0.23	0.55	0.67	0.16	98.81
aegis_post_1406a	71.5	5.5	2.2	0.8	0.45	0.07	8.6	3.4	1.3	14.1	3.2	2.3	1.8	1.6	0.2	2.2	1.1	0.4	4.43	0.77	0.43	1.26	0.99	0.27	104.64
aegis_post_1406a	51.1	5	0.4	1.56	0.63	0.12	6.3	3.2	0.6	17.8	3.8	0.9	3.3	1.7	0.2	6	2	0.6	4.93	0.86	0.3	0.83	0.79	0.09	91.85
aegis_post_1406a	63.4	5.3	0.8	0.88	0.47	0.11	13.4	3.6	1.8	13.8	3.2	1.8	1.2	1.5	0.2	1.8	1	0.5	5.44	0.93	0.43	2.07	1.19	0.22	102.05
aegis_post_1406a	57.1	5.2	2.8	0.83	0.46	0.04	8.7	3.5	0.8	21.8	4.3	0.8	2.5	1.6	0.7	0.7	0.9	0.5	4.14	0.72	0.52	1.23	0.98	0.18	97.02
aegis_post_1406a	47.7	4.9	0.5	1.2	0.54	0.08	8.6	3.4	0.2	20.9	4.2	1.2	3.5	1.7	0.3	1.8	1	0.3	3.29	0.64	0.27	0.87	0.81	0.1	87.8
aegis_post_1406a	49.7	4.9	0.4	1.22	0.54	0.08	6.7	3.3	0.5	18.5	3.9	0.7	3.7	1.7	0.3	2.6	1.1	0.2	3.4	0.65	0.26	0.93	0.84	0.2	86.72
aegis_post_1406a	56.9	5.2	0.8	0.93	0.48	0.03	16.1	3.7	0.9	14.5	3.3	1	2.1	1.6	0.3	5.8	1.9	1	4.45	0.77	0.33	1.3	1	0.15	102.04
Lucala	59.6	5.3	2.8	0.62	0.4	0.02	11.5	3.5	1.4	13.7	3.2	2.8	2.2	1.6	0.6	1.9	1	0.6	5.19	0.9	0.59	1.69	1.13	0.32	96.54
Lucala	47.5	4.9	0.9	1.87	0.72	0.15	4.4	3	0.7	18.3	3.8	0.5	3.1	1.7	0.2	7.9	2.5	0.5	4.28	0.74	0.34	0.59	0.68	0.07	87.92
Lucala	51.8	5	0.6	2.13	0.79	0.13	2.9	2.8	0.4	15.6	3.4	0.5	2.6	1.6	0.1	3.7	1.4	0.4	6.13	0.98	0.19	0.5	0.65	0.05	85.34
Lucala	58	5.2	0.8	1.73	0.68	0.12	12.8	3.6	0.7	15.7	3.5	0.7	1.3	1.5	0.1	3.1	1.2	0.4	5.31	0.91	0.2	1.99	1.19	0.2	99.9
Lucala	60.1	5.3	5	0.69	0.42	0.27	12.5	3.6	1.5	15.8	3.5	3.5	2.3	1.6	0.9	0.6	0.9	0.2	3.78	0.68	0.15	1.54	1.09	0.34	97.3
Cabamba	40.9	5.3	0.9	1.04	0.5	0.03	10	3.5	1.4	20.6	4.1	0.3	6.4	2	0.9	6.7	2.2	0.4	2.54	0.62	0.31	0.29	0.57	0.06	88.3
Cabamba	46.8	4.9	0.9	1.01	0.5	0.02	13.6	3.6	1.2	19	3.9	0.6	4.3	1.8	0.1	5.7	1.9	0.3	3.62	0.66	0.15	0.82	0.79	0.1	94.79
Cabamba	46.2	5	1	1.1	0.52	0.03	11	3.5	0.3	20.4	4.1	0.3	4	1.8	0.1	5.9	1.9	0.4	3.72	0.67	0.12	0.91	0.83	0.11	93.22
Cabamba	49.9	5	0.4	1.16	0.53	0.04	11.8	3.6	0.5	19.3	4	0.3	4.3	1.8	0.2	4.8	1.7	0.2	3.84	0.68	0.11	0.74	0.75	0.04	95.96
Cabamba	49.1	4.9	0.4	1.03	0.5	0.03	17.4	3.7	0.4	16.2	3.5	0.8	2.7	1.6	0.3	7.2	2.3	0.3	3.64	0.66	0.2	0.3	0.57	0.03	97.56
Cabamba	49.4	4.9	0.3	1.11	0.52	0.05	12.2	3.6	0.6	20.2	4.1	0.2	4.7	1.8	0.2	4.5	1.6	0.2	4.19	0.73	0.12	0.96	0.85	0.04	97.22
Cabamba	49.5	4.9	0.5	1.02	0.5	0.04	15.3	3.6	0.4	17.7	3.8	0.4	3.9	1.7	0.1	5.4	1.8	0.2	4.1	0.72	0.21	0.79	0.77	0.1	97.68
Cabamba	50.4	5	1.2	1.04	0.5	0.03	10.7	3.5	0.7	20.3	4.1	0.4	5.3	1.9	0.2	3.9	1.4	0.3	3.26	0.64	0.24	0.63	0.7	0.19	95.58
Cabamba	40.3	5.4	0.5	0.98	0.49	0.02	9.4	3.5	0.4	21.2	4.2	0.3	6.4	2.1	0.6	7.3	2.3	1.1	2.58	0.62	0.11	0.3	0.57	0.02	88.49
Cabamba	49	4.9	1.1	0.91	0.47	0.02	10.2	3.5	0.7	17.4	3.7	0.6	7	2.1	0.2	5.1	1.7	0.2	3.59	0.66	0.08	0.54	0.66	0.05	93.67
Bungo	43.2	5.1	0.4	0.91	0.47	0.03	10.5	3.5	0.5	20.2	4.1	0.2	9	2.4	0.2	5.1	1.7	0.4	2.38	0.62	0.08	0.14	0.52	0.01	91.39
Bungo	46	5	0.9	0.83	0.46	0.02	10.1	3.5	0.8	18.1	3.8	0.6	7.1	2.1	0.6	6.6	2.1	1.2	2.68	0.62	0.18	0.27	0.56	0.11	91.62
Bungo	45.7	5	0.7	0.89	0.47	0.01	7.9	3.4	0.8	20.1	4.1	0.5	8.7	2.4	0.5	5.4	1.8	0.5	2.54	0.62	0.09	0.22	0.55	0.03	91.49
Bungo	43.9	5.1	0.4	0.88	0.47	0.03	8.5	3.4	0.7	19.2	4	0.4	6.2	2	0.2	7.1	2.3	0.9	2.96	0.62	0.2	0.21	0.55	0.03	89.01
Bungo	44.6	5	0.3	0.89	0.47	0.03	8.2	3.4	0.2	19.1	4	0.3	7	2.1	0.2	6.9	2.2	0.3	2.82	0.62	0.1	0.34	0.59	0.02	89.94
Bungo	48.3	4.9	0.4	0.88	0.47	0.02	16.5	3.7	0.7	17.7	3.8	0.4	4.8	1.9	0.1	6.4	2.1	0.5	3.65	0.67	0.1	0.37	0.6	0.02	98.68
Bungo	43.6	5.1	0.5	0.96	0.49	0.03	11.4	3.5	0.8	19.1	4	0.4	6.3	2	0.4	8.1	2.5	0.9	2.8	0.62	0.16	0.21	0.55	0.04	92.46
Bungo	42.7	5.2	0.7	0.92	0.48	0.03	8.7	3.5	0.6	19.8	4	0.5	7.8	2.3	0.6	7.8	2.4	1.1	2.36	0.62	0.2	0.23	0.55	0.07	90.21
Bungo	43.8	5.1	0.4	0.92	0.48	0.02	8.9	3.5	0.9	20.8	4.2	0.5	8.5	2.4	0.4	5.6	1.9	0.7	2.48	0.62	0.11	0.17	0.53	0.02	91.14
Bungo	40.5	5.4	1.1	1.16	0.53	0.17	7.6	3.4	0.5	20.8	4.2	0.8	6.6	2.1	1	7.6	2.4	1.4	2.48	0.62	0.38	0.15	0.53	0.05	86.71
Sonneblom_ccam	53.6	5.1	1.1	0.93	0.48	0.06	6.4	3.2	0.6	17.3	3.7	0.2	3.2	1.7	0.1	7.9	2.5	0.5	4.55	0.79	0.29	0.73	0.75	0.06	94.61
Sonneblom_ccam	48.8	4.9	0.9	1.4	0.59	0.16	5.3	3.1	1.1	19.9	4.1	0.3	3.5	1.7	0.2	7.6	2.4	0.5	3.64	0.66	0.2	0.99	0.87	0.3	91.08
Sonneblom_ccam	53.9	5.1	2.3	0.77	0.44	0.01	9.3	3.5	1.6	16.6	3.6	1	2.2	1.6	0.3	6.9	2.2	1.9	4.18	0.73	0.26	1.36	1.03	0.41	95.11
Sonneblom_ccam	49.1	4.9	0.7	0.8	0.45	0.02	3.9	2.9	0.8	18.6	3.9	0.3	3.3	1.7	0.1	8.9	2.7	0.4	5.34	0.92	0.31	0.54	0.66	0.12	90.51
Sonneblom_ccam	61	5.3	2.4	0.59	0.39	0.02	7.1	3.3	1.2	15.7	3.5	1.7	2.2	1.6	0.3	4	1.4	0.6	5.1	0.89	0.46	0.89	0.82	0.15	96.56
Sonneblom_ccam	46.3	4.9	0.9	0.84	0.46	0.06	5.8	3.2	0.3	20	4.1	1.1	5.9	2	0.7	1	0.9	0.2	4.39	0.76	0.46	0.39	0.61	0.05	84.56
Sonneblom_ccam	50.6	5	0.4	1.2	0.54	0.06	3.9	2.9	0.3	19.9	4.1	0.3	3.3	1.7	0	7.2	2.3	0.2	5.16	0.89	0.21	0.46	0.63	0.04	91.78
Sonneblom_ccam	67.7	5.4	0.9	0.61	0.39	0.01	10.9	3.5	0.8	8.3	2.4	1.4	1.5	1.5	0.1	2	1	0.3	4.5	0.78	0.21	1.37	1.03	0.09	96.84
Sonneblom_ccam	58.5	5.2	2	0.96	0.49	0.07	10.4	3.5	0.8	19.6	4	1.7	1.9	1.6	0.6	1.5	1	0.3	4.91	0.85	0.28	0.8	0.78	0.13	98.61
Balombo	41.8	5.2	1.1	0.96	0.49	0.04	9.1	3.5	1.2	21.1	4.2	0.3	9.3	2.5	0.9	3.9	1.4	0.7	2.52	0.62	0.27	0.56	0.67	0.1	89.29
Balombo	44.3	5	1	1.17	0.53	0.07	11.6	3.6	2	19.7	4	0.6	6.4	2.1	0.6	6.5	2.1	0.4	3.02	0.63	0.22	0.58	0.68	0.09	93.28
Balombo	28.5	7	0.8	0.84	0.46	0.05	6.2	3.2	0.4	16.2	3.5	0.5	4.5	1.8	0.3	18.5	3.8	0.7	1.4	0.62	0.05	0.25	0.56	0.03	76.39
Balombo	42	5.2	0.6	0.94	0.48	0.03	10	3.5	0.4	20.1	4.1	0.5	7.4	2.2	0.4	7.9	2.5	0.8	2.65	0.62	0.13	0.64	0.71	0.09	91.54
Balombo	43.9	5.1	1	1.02	0.5	0.06	10	3.5	1.4	20.2	4.1	0.2	9.2	2.5	0.9	4.1	1.5	0.4	2.83	0.62	0.2	0.8	0.78	0.13	92.1
Balombo	44.1	5	0.6	0.86	0.46	0.06	11	3.5	1.2	17	3.7	0.4	6.8	2.1	0.7	9.7	2.8	0.5	3.05	0.63	0.23	0.76	0.76	0.11	93.37
Balombo	55.4	5.1	1.5	0.7	0.42	0.02	12.3	3.6	1.5	15.9	3.5	0.9	5	1.9	0.8	4.1	1.5	0.8	3.68	0.67	0.38	1.31	1.01	0.27	98.28
Balombo	44.4	5	0.4	0.96	0.49	0.05	9.9	3.5	0.7	19.9	4.1	0.4	8.3	2.3	1.2	6	2	0.8	2.86	0.62	0.16	0.81	0.78	0.05	93.07
Balombo	44.4	5	0.7	0.86	0.46	0.04	7.1	3.3	0.5	18.5	3.9	0.3	11.1	2.8	0.6	7	2.2	1.5	2.27	0.62	0.1	0.78	0.77	0.07	92.07
Balombo	50.1	5	0.5	0.86	0.46	0.02	15.6	3.6	0.3	13.3	3.1	0.3	3.1	1.7	0.2	6.9	2.2	0.3	5.9	0.97	0.14	0.43	0.62	0.03	96.13
Seeheim	60.5	5.3	0.3	0.77	0.44	0.02	16.5	3.7	0.3	5.5	2.1	0.4	2.7	1.6	0.2	3.3	1.3	0.1	6.44	1	0.09	2.37	1.2	0.06	98.11
Seeheim	62.3	5.3	0.6	0.82	0.45	0.02	15.3	3.6	0.5	6	2.1	0.5	1.9	1.6	0.1	3.3	1.3	0.3	5.81	0.96	0.2	2.03	1.19	0.11	97.47
Seeheim	47.1	4.9	0.5	0.91	0.47	0.02	13.9	3.6	1	17.1	3.7	0.5	5.2	1.9	0.2	7.1	2.3	0.6	3.93	0.69	0.13	0.48	0.64	0.09	95.78
Seeheim	45.9	5	0.5	0.95	0.48	0.04	9.7	3.5	0.8	19.5	4	0.3	7.3	2.2	0.3	7.2	2.3	0.3	3.1	0.63	0.23	0.28	0.57	0.04	93.92
Seeheim	40.2	5.4	0.8	1	0.49	0.04	10.5	3.5	0.9	20.2	4.1	0.3	5.2	1.9	0.2	9.4	2.8	0.4	2.6	0.62	0.27	0.15	0.53	0.04	89.24
Wilhelmstal	46.7	4.9	0.4	0.93	0.48	0.03	10.9	3.5	0.8	19.8	4	0.4	6.7	2.1	0.3	4.9	1.7	0.5	3.62	0.66	0.11	0.4	0.61	0.02	93.93
Wilhelmstal	43.7	5.1	0.7	0.99	0.49	0.04	10	3.5	0.8	20.2	4.1	0.3	7.2	2.2	0.2	5.2	1.8	0.3	3.79	0.68	0.1	0.35	0.59	0.03	91.38
Wilhelmstal	48.1	4.9	0.6	0.9	0.47	0.03	11.1	3.5	0.4	17.7	3.8	0.4	6.8	2.1	0.1	5.9	1.9	0.1	4.26	0.74	0.17	0.67	0.72	0.01	95.44
Wilhelmstal	45.3	5	1.5	0.88	0.47	0.06	10	3.5	1.1	19	3.9	0.9	8.8	2.4	0.4	5.5	1.8	0.6	2.92	0.62	0.12	0.31	0.58	0.02	92.62
Wilhelmstal	47.1	4.9	0.6	0.88	0.47	0.03	11.6	3.6	0.9	18.7	3.9	0.5	7.2	2.2	0.3	6.1	2	0.4	3.14	0.63	0.17	0.29	0.57	0.01	95
Wilhelmstal	46.2	5	0.3	0.91	0.47	0.02	12.7	3.6	0.9	18.9	3.9	0.3	6.6	2.1	0.3	5.7	1.9	0.3	3.42	0.65	0.13	0.37	0.6	0.01	94.76
Wilhelmstal	44	5.1	0.4	1.02	0.5	0.03	9.5	3.5	0.4	19.9	4.1	0.3	7.5	2.2	0.2	6.1	2	0.2	3.02	0.63	0.19	0.34	0.59	0.01	91.35
Wilhelmstal	45.6	5	0.4	1	0.49	0.03	11.6	3.6	0.8	19.2	4	0.3	8.2	2.3	0.2	7	2.3	0.2	2.96	0.62	0.1	0.25	0.56	0.02	95.89
Wilhelmstal	43.2	5.1	0.3	1.03	0.5	0.02	11.6	3.6	0.7	19.1	3.9	0.3	6	2	0.1	5.9	1.9	0.2	3.17	0.63	0.06	0.35	0.59	0.01	90.25
Oranjemund	40.6	5.4	0.7	1.16	0.53	0.06	6.5	3.2	0.2	21.6	4.3	0.3	11.2	2.8	0.3	4.4	1.5	0.6	2.66	0.62	0.11	0.23	0.55	0.06	88.37
Oranjemund	43.5	5.1	0.5	0.93	0.48	0.04	6.9	3.3	0.2	19.7	4	0.4	12.4	2.9	1.1	6	2	1.2	1.9	0.63	0.06	0.68	0.72	0.07	92.01
Oranjemund	42	5.2	0.4	1.11	0.52	0.08	5.1	3.1	0.1	20.3	4.1	0.2	12.8	3	0.3	5.4	1.8	0.4	2.23	0.62	0.09	0.42	0.62	0.09	89.32
Oranjemund	45.1	5	1.4	1.21	0.54	0.08	6.8	3.3	0.7	20.3	4.1	0.3	11.1	2.8	0.6	4.1	1.5	0.9	2.36	0.62	0.14	1.22	0.97	0.19	92.19
Oranjemund	44.8	5	0.6	1.31	0.57	0.08	7	3.3	0.3	19.8	4	0.3	9.6	2.5	0.3	5.8	1.9	0.5	2.1	0.63	0.17	1.21	0.97	0.13	91.58
Oranjemund	44.5	5	0.4	1.22	0.54	0.13	6.1	3.2	0.3	19.7	4	0.3	11.6	2.8	0.8	6.3	2.1	0.7	1.77	0.64	0.09	0.7	0.73	0.05	91.79
Oranjemund	44.7	5	1.1	1.35	0.58	0.13	7	3.3	0.2	19.5	4	0.3	9	2.4	0.6	6.7	2.2	0.8	2.04	0.63	0.1	1.27	0.99	0.1	91.61
Oranjemund	44.6	5	0.9	1.05	0.5	0.04	5.9	3.2	0.4	19.5	4	0.2	11	2.8	0.5	6.4	2.1	1.3	2.14	0.62	0.25	0.98	0.86	0.09	91.6
Oranjemund	46.9	4.9	0.7	1.12	0.52	0.02	7.1	3.3	0.1	19.7	4	0.3	12.3	2.9	0.6	3.1	1.2	0.3	2.06	0.63	0.13	1.42	1.05	0.16	93.82
Oranjemund	44.4	5	0.5	1.19	0.54	0.04	6.3	3.2	0.3	20.5	4.1	0.3	12.3	2.9	0.3	3.4	1.3	0.3	1.88	0.63	0.08	1.08	0.91	0.08	91.02
Seeis	54.1	5.1	2.8	0.7	0.42	0.02	9.6	3.5	1.4	14.8	3.3	0.8	2.7	1.6	0.3	8.6	2.6	1.8	4.86	0.85	0.19	1.44	1.06	0.23	96.85
Seeis	51.2	5	2.2	1.67	0.66	0.17	5.2	3.1	0.5	18.8	3.9	1.4	3.1	1.7	0.5	2.7	1.2	1	5.17	0.9	0.42	0.27	0.56	0.06	88.14
Seeis	65.6	5.3	1.8	0.7	0.42	0.02	15.4	3.6	2.1	4.6	2	2.8	0.6	1.5	0.3	0.9	0.9	0.5	5.5	0.94	0.28	2.75	1.17	0.18	96.11
Seeis	51.8	5	1.4	2.29	0.85	0.42	7.1	3.3	0.7	18.8	3.9	0.2	2.7	1.6	0.2	7.4	2.3	1.3	4.76	0.83	0.25	1.4	1.04	0.22	96.28
Seeis	50	5	1	0.8	0.45	0.04	4.8	3	0.8	18.1	3.8	0.4	2.9	1.7	0.1	7.9	2.5	0.3	4.95	0.86	0.18	0.44	0.62	0.06	90.02
Seeis	64.4	5.3	1.3	0.76	0.44	0.15	12.6	3.6	1.4	16.8	3.6	1.5	1.2	1.5	0.3	0.7	0.9	0.3	3.85	0.68	0.32	1.52	1.09	0.18	101.8
Seeis	55.1	5.1	4.4	1.21	0.54	0.41	12.1	3.6	1.7	18.5	3.9	1.9	2.5	1.6	1	2.6	1.1	1.3	4.33	0.75	0.73	1.59	1.11	0.45	97.89
Seeis	46.9	4.9	0.9	0.66	0.41	0.01	5.1	3.1	0.5	17.5	3.7	0.3	2.8	1.6	0.1	11.8	3.1	0.5	3.39	0.65	0.2	0.37	0.6	0.09	88.47
Seeis	48.5	4.9	0.4	0.94	0.48	0.06	6.8	3.3	0.4	18.3	3.8	0.2	3.8	1.7	0.1	10.8	3	0.3	4.34	0.75	0.2	0.54	0.66	0.07	93.93
Mariental	43.8	5.1	0.4	0.85	0.46	0.05	12.3	3.6	0.2	17.8	3.8	0.2	5	1.9	0.1	12.9	3.2	0.3	2.51	0.62	0.06	0.14	0.52	0.01	95.4
Mariental	44.1	5	0.4	1.16	0.53	0.03	17.6	3.7	0.3	23.8	4.5	0.7	3.7	1.7	0.1	4.8	1.7	0.8	4.85	0.84	0.09	0.7	0.73	0.04	100.82
Mariental	51.4	5	0.7	0.71	0.42	0.01	18.5	3.7	0.6	9.7	2.6	1.1	4.8	1.8	0.4	12.2	3.2	1.5	4.21	0.73	0.31	0.18	0.54	0.07	101.75
Mariental	55.2	5.1	1	1.68	0.67	0.23	13.6	3.6	0.9	18.6	3.9	1	5.3	1.9	0.1	4.6	1.6	0.4	3.56	0.66	0.21	0.72	0.74	0.05	103.23
Mariental	48.8	4.9	0.8	0.84	0.46	0.03	15.6	3.6	0.5	18.1	3.8	0.2	7.3	2.2	0.2	6.4	2.1	0.3	3.46	0.65	0.2	0.44	0.62	0.04	100.81

9. Compositions from Individual ChemCam Observation Points: Blackfoot, Brandberg

Major-element abundances (wt. %) of all ChemCam observation points in (and in some cases near) the Blackfoot and Brandberg units, as well as several targets on Zabriskie Plateau that appear related to the Bimbe Layered targets. Columns are included for accuracies and for standard deviations of the 25 individual spectra within each observation point. Observation points are arranged consecutively for each target. Data from target Gibeon were of low quality and were not archived.

Sequence	Target	Location	Dist. (m)	SiO2	acc	stdev	TiO2	acc	stdev	Al2O3	acc	stdev	FeOT	acc	stdev	MgO	acc	stdev	CaO	acc	stdev	Na2O	acc	stdev	K2O	acc	stdev	Total
01100	Swan	Blackfoot	2.83	50.9	5	0.9	1.16	0.53	0.04	14.6	3.6	1.1	16.5	3.6	0.9	3.9	1.7	0.4	7	2.3	0.3	4.08	0.71	0.34	1.49	1.06	0.18	99.62
01100	Swan	Blackfoot	2.83	51.6	5	0.4	1.25	0.55	0.14	15.1	3.6	0.5	15.6	3.5	0.3	4.3	1.8	0.2	6.5	2.1	0.4	4.16	0.72	0.22	1.53	1.07	0.12	100.13
01100	Swan	Blackfoot	2.83	48.9	4.9	0.7	1.42	0.59	0.08	12.8	3.6	0.3	19.6	4.2	0.5	5.1	1.9	0.3	5.1	1.7	0.5	3.15	0.63	0.16	1.65	1.1	0.14	97.82
01100	Swan	Blackfoot	2.83	47.4	4.9	0.7	1.09	0.51	0.06	11.3	3.5	0.4	17.5	3.8	0.4	6.3	2	0.5	7.9	2.5	0.3	2.43	0.61	0.11	0.69	0.74	0.05	94.63
01100	Swan	Blackfoot	2.83	47	5	1.2	1.53	0.62	0.07	11	3.5	0.7	19.4	4.1	0.8	4.9	1.8	0.3	6.8	2.2	0.6	2.58	0.61	0.16	1.36	1.01	0.17	94.58
01100	Swan	Blackfoot	2.83	46.7	5	0.6	1.47	0.61	0.09	12.2	3.6	0.5	20.9	4.3	0.5	4.8	1.8	0.2	5.2	1.7	0.4	2.61	0.61	0.14	1.18	0.94	0.12	95.08
01100	Swan	Blackfoot	2.83	45.7	5	0.6	1.41	0.59	0.13	10.1	3.5	0.5	18.2	3.9	0.2	7.2	2.1	0.4	5.1	1.7	0.7	2.36	0.61	0.1	1.06	0.89	0.07	91.28
01100	Swan	Blackfoot	2.83	49.3	5	0.7	0.82	0.45	0.07	16	3.7	0.3	17.2	3.7	0.3	4.6	1.8	0.3	7.1	2.3	0.3	3.32	0.64	0.18	0.56	0.68	0.05	98.85
01100	Swan	Blackfoot	2.83	43.8	5.1	0.4	1.95	0.74	0.17	10.2	3.5	0.2	20.9	4.3	0.2	6.6	2.1	0.2	6.3	2.1	0.3	2.4	0.61	0.08	0.91	0.83	0.06	93.02
01100	Swan	Blackfoot	2.83	47.6	4.9	1.2	1.3	0.56	0.12	13.4	3.6	0.5	19.1	4.1	0.7	5.6	1.9	0.2	5.4	1.8	0.4	2.89	0.62	0.11	1.06	0.89	0.19	96.42
02100	Sunburst	Blackfoot	2.37	42.1	5.2	0.8	0.83	0.46	0.05	9	3.5	0.8	20.2	4.3	0.2	14.2	3.2	0.7	3.5	1.3	0.5	1.54	0.65	0.12	0.19	0.55	0.04	91.55
02100	Sunburst	Blackfoot	2.37	41.6	5.3	0.7	1.31	0.57	0.11	8.1	3.4	0.2	19.6	4.2	0.2	11.8	2.9	0.4	3.6	1.3	0.1	1.67	0.65	0.14	0.23	0.57	0.05	87.93
02100	Sunburst	Blackfoot	2.37	41.8	5.3	0.5	1.01	0.5	0.05	7.3	3.3	0.3	19.8	4.2	0.2	13.2	3.1	0.4	3.7	1.3	0.3	1.48	0.64	0.09	0.14	0.54	0.02	88.48
02100	Sunburst	Blackfoot	2.37	43	5.1	0.7	0.77	0.44	0.02	7.8	3.4	0.4	18.9	4	0.5	12.4	3	1.4	6.1	2	1.1	1.6	0.65	0.14	0.12	0.53	0.03	90.74
02100	Sunburst	Blackfoot	2.37	41.5	5.3	0.8	0.91	0.47	0.04	8.5	3.4	0.5	18.9	4.1	0.6	12.7	3	0.7	4.5	1.6	0.9	1.56	0.65	0.16	0.17	0.55	0.06	88.89
02100	Sunburst	Blackfoot	2.37	41.9	5.2	0.5	0.9	0.47	0.06	7.8	3.4	0.1	19.1	4.1	0.2	12.5	3	0.3	4.2	1.5	0.2	1.64	0.65	0.04	0.21	0.56	0.02	88.26
02100	Sunburst	Blackfoot	2.37	45	5	0.6	0.82	0.45	0.07	7.9	3.4	0.3	18.5	4	0.2	12.7	3	0.5	5.2	1.7	0.8	1.53	0.65	0.08	0.31	0.59	0.06	91.9
02100	Sunburst	Blackfoot	2.37	45.2	5	0.5	0.94	0.48	0.04	10.1	3.5	0.4	19.6	4.2	0.4	11.3	2.8	0.3	4.1	1.4	0.5	2.15	0.62	0.07	0.33	0.6	0.02	93.75
02100	Sunburst	Blackfoot	2.37	41.8	5.3	0.7	0.7	0.42	0.02	7.4	3.3	0.9	18.7	4	0.3	11.9	2.9	0.4	7.7	2.4	1.6	1.35	0.63	0.2	0.13	0.54	0.02	89.57
02100	Sunburst	Blackfoot	2.37	40.4	5.4	0.5	0.96	0.49	0.07	8.5	3.4	0.4	20.6	4.3	0.2	12	2.9	0.5	4.8	1.6	0.4	1.7	0.65	0.09	0.24	0.57	0.04	89.1
01102	Madison	Blackfoot	4.56	55.2	5.1	1.2	1.34	0.57	0.07	17.4	3.7	1.5	19.6	4.2	0.8	2.1	1.6	0.3	2.6	1.1	0.3	4.55	0.79	0.28	2.2	1.18	0.17	104.93
01102	Madison	Blackfoot	4.56	6.7	6.7	3	0.2	0.25	0.11	1.3	2.6	0.3	1	1.6	1.1	1.6	1.5	0.3	45	8.2	1.4	0.43	0.38	0.05	0.17	0.55	0.03	56.39
01102	Madison	Blackfoot	4.56	8.3	6.7	4.2	0.46	0.34	0.13	0.7	2.6	0.1	2.8	1.8	1.6	2	1.6	0.3	43.5	8	2.4	0.44	0.38	0.04	0.18	0.55	0.06	58.23
01102	Madison	Blackfoot	4.56	9.8	6.7	2.7	0.5	0.36	0.03	1	2.6	0.2	4.1	1.9	1	2.2	1.6	0.3	42.5	7.8	2	0.59	0.43	0.09	0.21	0.56	0.04	60.87
01102	Madison	Blackfoot	4.56	10.6	6.7	3.2	0.53	0.37	0.08	0.7	2.6	0.1	4.6	2	1.4	2.4	1.6	0.2	39.3	7.3	1.9	0.51	0.4	0.1	0.13	0.54	0.03	58.71
02102	Jefferson	Blackfoot	2.97	41.9	5.2	0.6	1.38	0.58	0.12	7.4	3.4	0.4	21.5	4.4	0.7	11.1	2.8	0.5	3.2	1.2	0.4	2.2	0.62	0.19	0.35	0.61	0.08	89.11
02102	Jefferson	Blackfoot	2.97	44.9	5	0.6	1.26	0.55	0.07	8.7	3.5	0.7	20.8	4.3	0.2	9.2	2.5	1.2	4.3	1.5	0.6	2.63	0.61	0.18	0.36	0.61	0.04	92.08
02102	Jefferson	Blackfoot	2.97	46	5	0.7	1.4	0.59	0.11	12.9	3.6	0.8	19.6	4.2	0.2	6.8	2.1	0.5	5.6	1.9	0.3	3.04	0.62	0.23	0.6	0.7	0.08	95.88
02102	Jefferson	Blackfoot	2.97	43.4	5.1	0.4	0.86	0.46	0.1	10.3	3.5	0.6	20.5	4.3	0.3	11.8	2.9	1.4	4.4	1.5	0.3	2.18	0.62	0.12	0.26	0.58	0.07	93.82
02102	Jefferson	Blackfoot	2.97	44.2	5.1	0.7	1.31	0.57	0.07	10.4	3.5	1	20.4	4.3	0.2	9.5	2.5	1.3	4.3	1.5	0.4	2.15	0.62	0.18	0.26	0.58	0.05	92.49
02102	Jefferson	Blackfoot	2.97	41.5	5.3	0.6	0.87	0.47	0.03	6.9	3.3	0.4	21	4.4	0.3	14.3	3.2	1.3	4.3	1.5	0.6	1.92	0.64	0.09	0.24	0.57	0.03	91.13
02102	Jefferson	Blackfoot	2.97	45.1	5	1.3	0.97	0.49	0.06	10	3.5	1.5	20	4.2	0.4	9	2.4	1.1	5.5	1.8	0.4	2.42	0.61	0.25	0.43	0.63	0.06	93.46
02102	Jefferson	Blackfoot	2.97	42.2	5.2	1	1.96	0.74	0.19	8.7	3.5	0.9	20.7	4.3	0.4	8.8	2.4	0.5	2.3	1.1	0.2	2.47	0.61	0.17	0.5	0.66	0.06	87.71
02102	Jefferson	Blackfoot	2.97	42.5	5.2	0.7	1.29	0.56	0.06	9	3.5	0.8	20.1	4.2	0.3	10.2	2.6	0.9	5.1	1.7	0.4	2.17	0.62	0.27	0.38	0.62	0.06	90.8
02102	Jefferson	Blackfoot	2.97	48.6	4.9	0.5	1.35	0.58	0.09	17.8	3.7	0.7	17.9	3.9	0.6	4.4	1.8	0.5	4.7	1.6	0.2	3.68	0.66	0.1	0.62	0.71	0.12	98.97
04102	Lincoln	Blackfoot	2.97	43.7	5.1	0.6	1.73	0.68	0.09	11.4	3.5	0.5	20.7	4.3	1	4.7	1.8	0.1	4.7	1.6	0.3	3.32	0.64	0.1	0.83	0.79	0.05	91.08
04102	Lincoln	Blackfoot	2.97	48.7	4.9	1.4	0.98	0.49	0.06	10.6	3.5	0.9	20.5	4.3	0.5	9	2.4	0.8	3.4	1.3	0.3	2.68	0.61	0.24	0.98	0.86	0.19	96.96
04102	Lincoln	Blackfoot	2.97	45.2	5	0.6	0.89	0.47	0.04	8.8	3.5	0.3	21.6	4.4	0.2	12.8	3	0.6	4.1	1.4	0.4	2.08	0.63	0.1	0.42	0.63	0.08	95.88
04102	Lincoln	Blackfoot	2.97	47	5	0.5	0.91	0.47	0.03	11.3	3.5	0.4	18.7	4	0.3	7.5	2.2	0.6	5.5	1.8	0.2	2.87	0.62	0.13	0.82	0.79	0.06	94.65
04102	Lincoln	Blackfoot	2.97	46.3	5	0.5	0.85	0.46	0.02	16.2	3.7	0.1	20.9	4.3	0.3	5.1	1.9	0.4	5	1.7	0.3	3.25	0.64	0.14	0.21	0.56	0.02	97.85
01160	Roter_Kamm	Brandberg	3.76	44.2	5.1	0.8	0.99	0.49	0.11	11.7	3.6	0.4	21.7	4.4	0.4	10.1	2.6	1.1	3	1.2	0.9	2.69	0.61	0.1	1.52	1.07	0.12	95.9
01160	Roter_Kamm	Brandberg	3.76	49.7	5	0.7	0.82	0.45	0.06	13.3	3.6	0.4	21.3	4.4	0.3	7.6	2.2	0.6	3.2	1.2	0.4	3.13	0.63	0.15	1.88	1.15	0.1	100.98
01160	Roter_Kamm	Brandberg	3.76	45.6	5	0.7	0.93	0.48	0.2	13.3	3.6	0.5	20.5	4.3	0.6	3.7	1.7	0.2	8.7	2.7	1	2.83	0.62	0.1	1.36	1.01	0.14	96.93
01160	Roter_Kamm	Brandberg	3.76	53.8	5.1	0.5	0.92	0.48	0.05	15.2	3.6	0.7	19.1	4.1	0.6	6.6	2.1	0.4	3.4	1.3	0.4	3.64	0.66	0.09	1.76	1.13	0.06	104.42
01160	Roter_Kamm	Brandberg	3.76	52.8	5	0.5	0.87	0.47	0.04	16.7	3.7	0.3	19	4.1	0.5	5.2	1.9	0.2	4.7	1.6	0.5	3.61	0.66	0.18	1.61	1.09	0.1	104.45
02160	Hoba	Brandberg	4.56	50	5	0.6	0.79	0.45	0.02	16.4	3.7	0.5	21	4.4	0.6	7	2.1	0.2	2.3	1	0.4	3.67	0.66	0.08	2.03	1.17	0.11	103.21
02160	Hoba	Brandberg	4.56	51.8	5	0.6	0.83	0.46	0.03	17.1	3.7	0.6	19.7	4.2	0.8	7.1	2.1	0.3	2.7	1.1	0.3	3.95	0.69	0.08	1.83	1.14	0.08	104.91
02160	Hoba	Brandberg	4.56	52.3	5	0.6	0.88	0.47	0.04	16.8	3.7	0.9	20.1	4.2	0.8	5.7	1.9	0.2	3.2	1.2	0.3	3.58	0.66	0.13	2.18	1.18	0.15	104.76
02160	Hoba	Brandberg	4.56	41.6	5.3	4.5	0.78	0.44	0.1	9.9	3.5	0.7	20.4	4.3	0.8	5.7	1.9	1.1	9.4	2.8	2.8	2.1	0.62	0.42	0.57	0.69	0.12	90.42
02160	Hoba	Brandberg	4.56	48.8	4.9	1.2	0.88	0.47	0.05	14.8	3.6	0.8	21.1	4.4	0.8	9	2.4	0.3	2.2	1	0.3	2.96	0.62	0.15	1.7	1.11	0.12	101.45
01692	Pyramid Hills	Zabriskie Plat.	2.26	38.8	5.6	0.4	1.05	0.5	0.11	6.1	3.2	0.4	18.9	4	0.2	10.7	2.7	0.4	5.4	1.8	0.4	2.56	0.61	0.11	0.36	0.61	0.04	83.9
01692	Pyramid Hills	Zabriskie Plat.	2.26	41.1	5.3	0.8	0.97	0.49	0.05	4.8	3	0.3	19.8	4.2	0.2	12.5	3	0.5	3.8	1.4	0.2	1.87	0.64	0.06	0.74	0.76	0.06	85.54
01692	Pyramid Hills	Zabriskie Plat.	2.26	44.1	5.1	0.5	1.18	0.53	0.11	5.8	3.2	0.2	19	4.1	0.1	10.1	2.6	0.2	4.8	1.7	0.5	2.29	0.62	0.07	1.48	1.05	0.16	88.67
01692	Pyramid Hills	Zabriskie Plat.	2.26	43.1	5.1	0.4	1.09	0.51	0.11	6.3	3.2	0.3	19.6	4.2	0.3	9	2.4	0.2	5.4	1.8	0.4	2.1	0.62	0.12	1.01	0.87	0.09	87.62
01692	Pyramid Hills	Zabriskie Plat.	2.26	42.7	5.2	1.7	1.3	0.56	0.18	4.9	3	0.6	19.5	4.1	0.3	11.5	2.8	0.4	5.6	1.9	0.5	1.8	0.64	0.09	1.36	1.01	0.12	88.69
01694	Johnnie	Zabriskie Plat.	4.26	45.8	5	0.5	0.8	0.45	0.02	8.6	3.5	0.5	19.6	4.2	0.4	11.3	2.8	0.8	6.9	2.2	0.4	2.68	0.61	0.2	0.76	0.76	0.09	96.44
01694	Johnnie	Zabriskie Plat.	4.26	41.3	5.3	0.9	0.86	0.46	0.03	8.4	3.4	0.4	24.5	4.6	1.2	13.1	3.1	0.6	2.9	1.2	0.3	2.38	0.61	0.19	0.99	0.86	0.1	94.49
01694	Johnnie	Zabriskie Plat.	4.26	40.1	5.5	0.5	1.42	0.59	0.05	7.5	3.4	0.2	28.3	4.9	0.8	10.2	2.6	0.6	4.4	1.5	0.5	2.5	0.61	0.1	0.86	0.81	0.08	95.3
01694	Johnnie	Zabriskie Plat.	4.26	45.1	5	0.4	1.02	0.5	0.05	9.1	3.5	0.4	21.7	4.4	0.5	11.5	2.8	0.5	3.4	1.3	0.2	2.73	0.61	0.12	1.15	0.93	0.08	95.78
01694	Johnnie	Zabriskie Plat.	4.26	42.1	5.2	0.5	1.89	0.72	0.06	9.5	3.5	0.3	27.8	4.9	0.7	6.7	2.1	0.4	4.2	1.5	0.4	2.99	0.62	0.11	0.91	0.83	0.08	96.19
02694	South_Park2	Zabriskie Plat.	2.24	39.6	5.5	0.2	0.87	0.47	0.02	5.1	3.1	0.3	19.2	4.1	0.2	13.4	3.1	0.3	5.5	1.8	0.3	1.73	0.65	0.05	0.19	0.55	0.02	85.58
02694	South_Park2	Zabriskie Plat.	2.24	39.4	5.5	0.3	0.87	0.47	0.04	6.5	3.2	0.6	18.9	4.1	0.5	10.9	2.7	0.6	6.3	2.1	0.9	1.73	0.65	0.17	0.23	0.57	0.04	84.88
02694	South_Park2	Zabriskie Plat.	2.24	44.3	5.1	1	1.02	0.5	0.08	8.5	3.4	0.3	18.5	4	0.6	6.1	2	0.2	6.8	2.2	0.6	2.99	0.62	0.14	0.51	0.66	0.03	88.79
02694	South_Park2	Zabriskie Plat.	2.24	43.7	5.1	0.7	0.89	0.47	0.03	6.8	3.3	0.7	19.4	4.1	0.3	12.9	3	1.4	3.1	1.2	0.7	2.37	0.61	0.15	0.32	0.6	0.03	89.42
02694	South_Park2	Zabriskie Plat.	2.24	42.6	5.2	0.7	0.73	0.43	0.03	8.4	3.4	0.5	17.8	3.9	0.4	10.2	2.6	1.4	6.9	2.2	1	2.73	0.61	0.14	0.61	0.7	0.06	90.06

10. Images of ChemCam Heterolithic-Unit Targets Not in the Main Body of the Paper.

These images are available with the given target names in the Planetary Data System at https://pds-geosciences.wustl.edu/msl/msl-m-chemcam-libs-4_5-rdr-v1/mslccm_1xxx/extras/

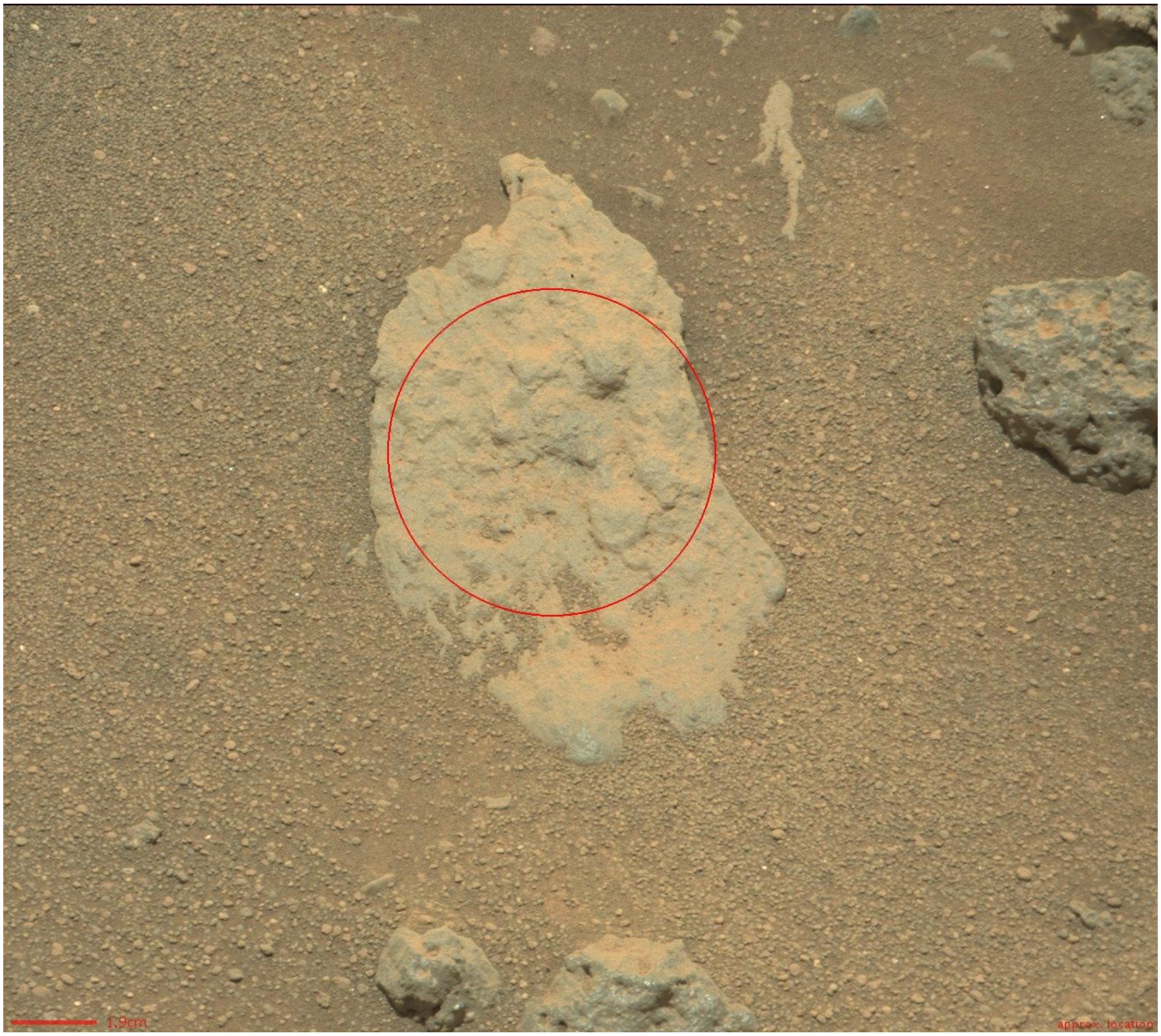

AEGIS_post_1400a, Mastcam image.

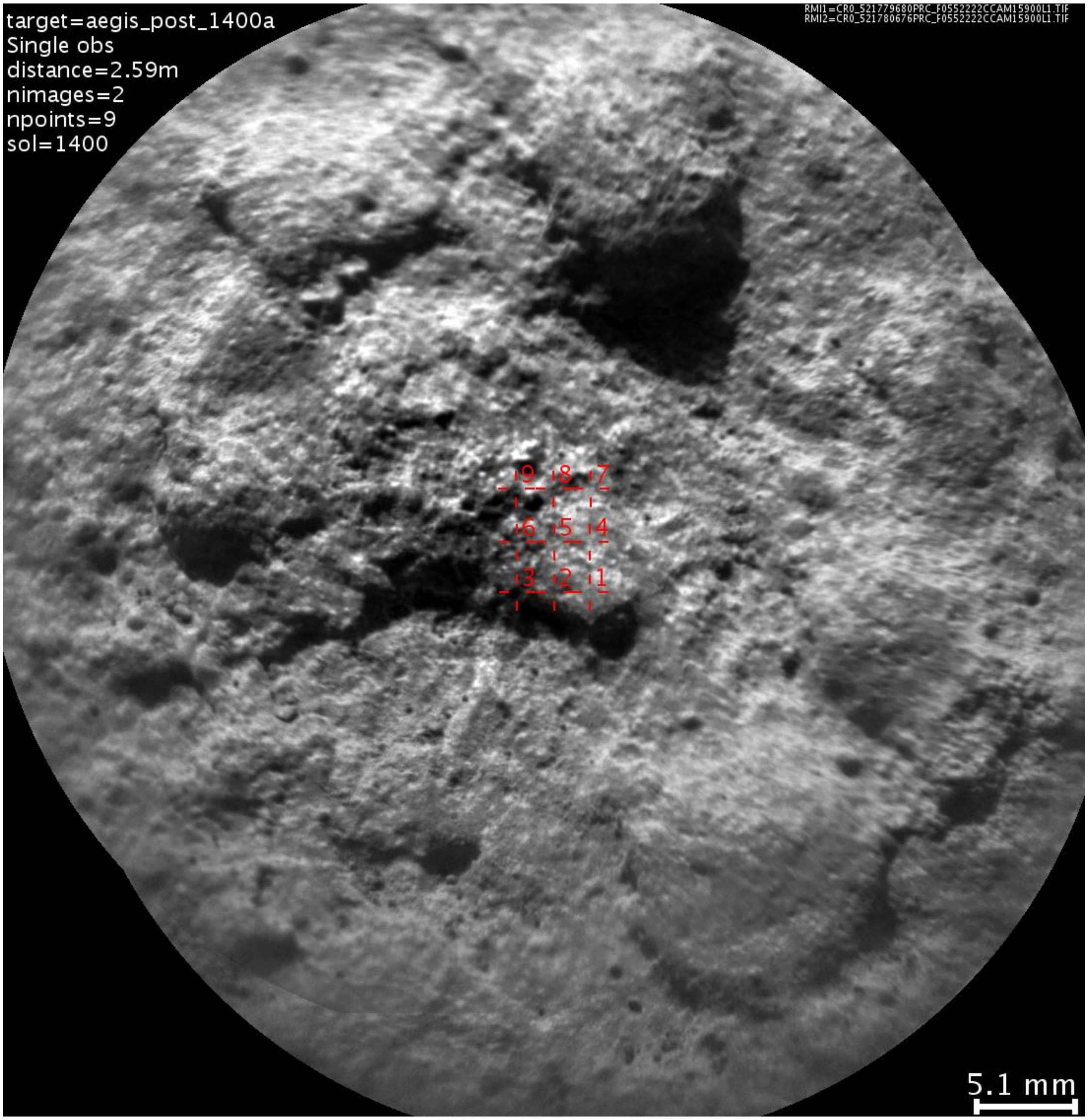

AEGIS_post_1400a, RMI mosaic.

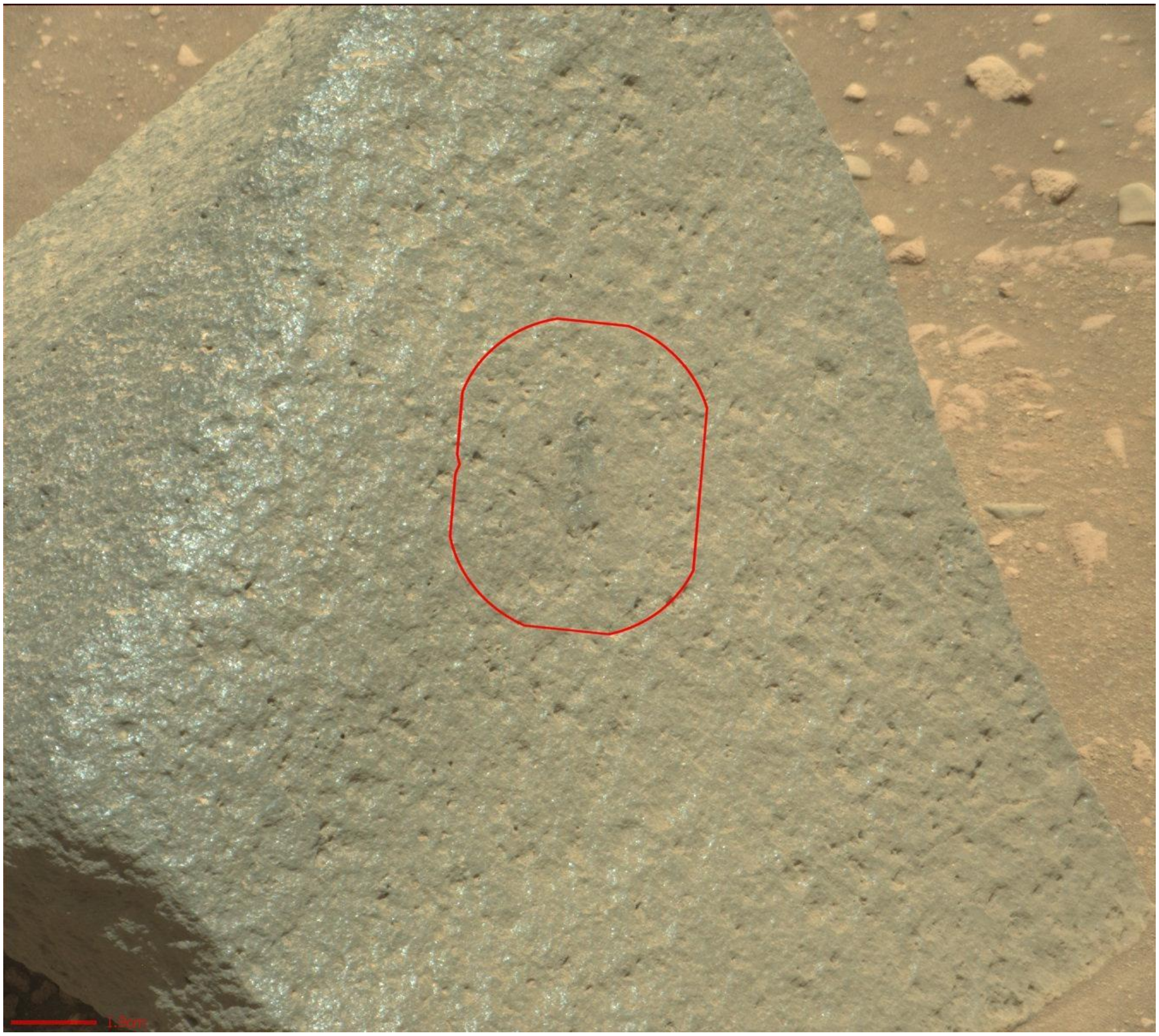

Aussenkehr, Mastcam image.

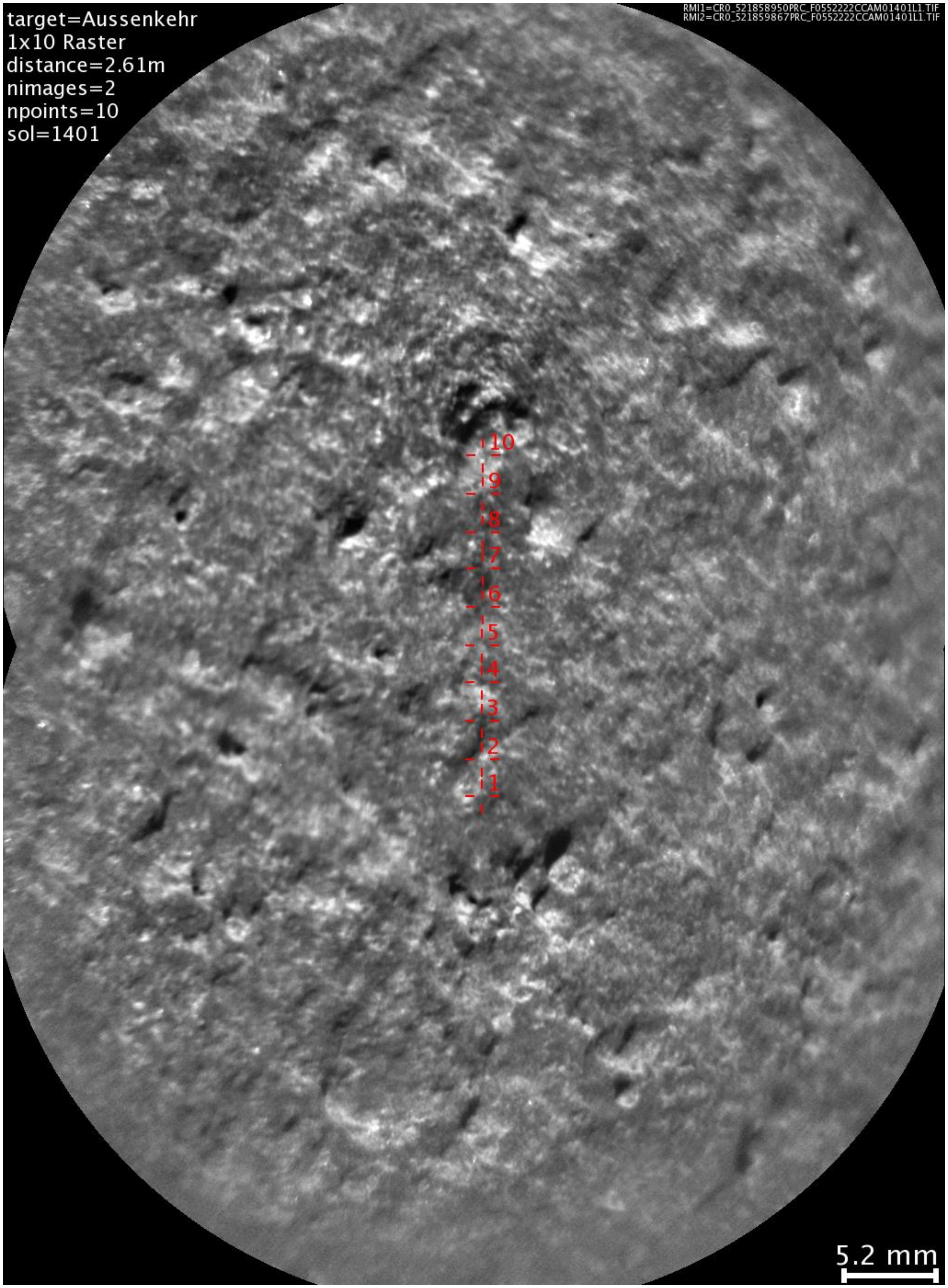

Aussenkehr, RMI mosaic.

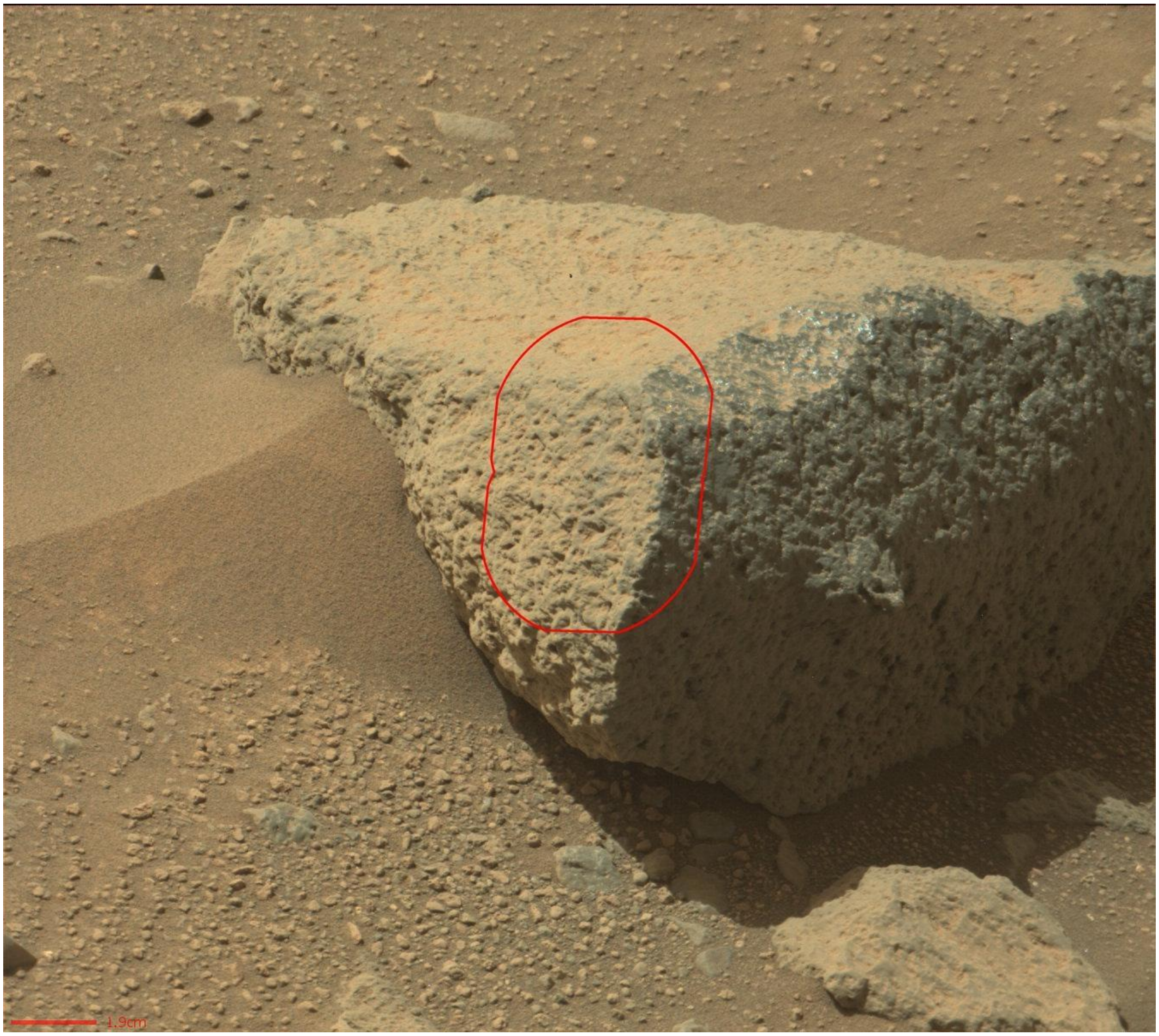

Lucala, Mastcam image.

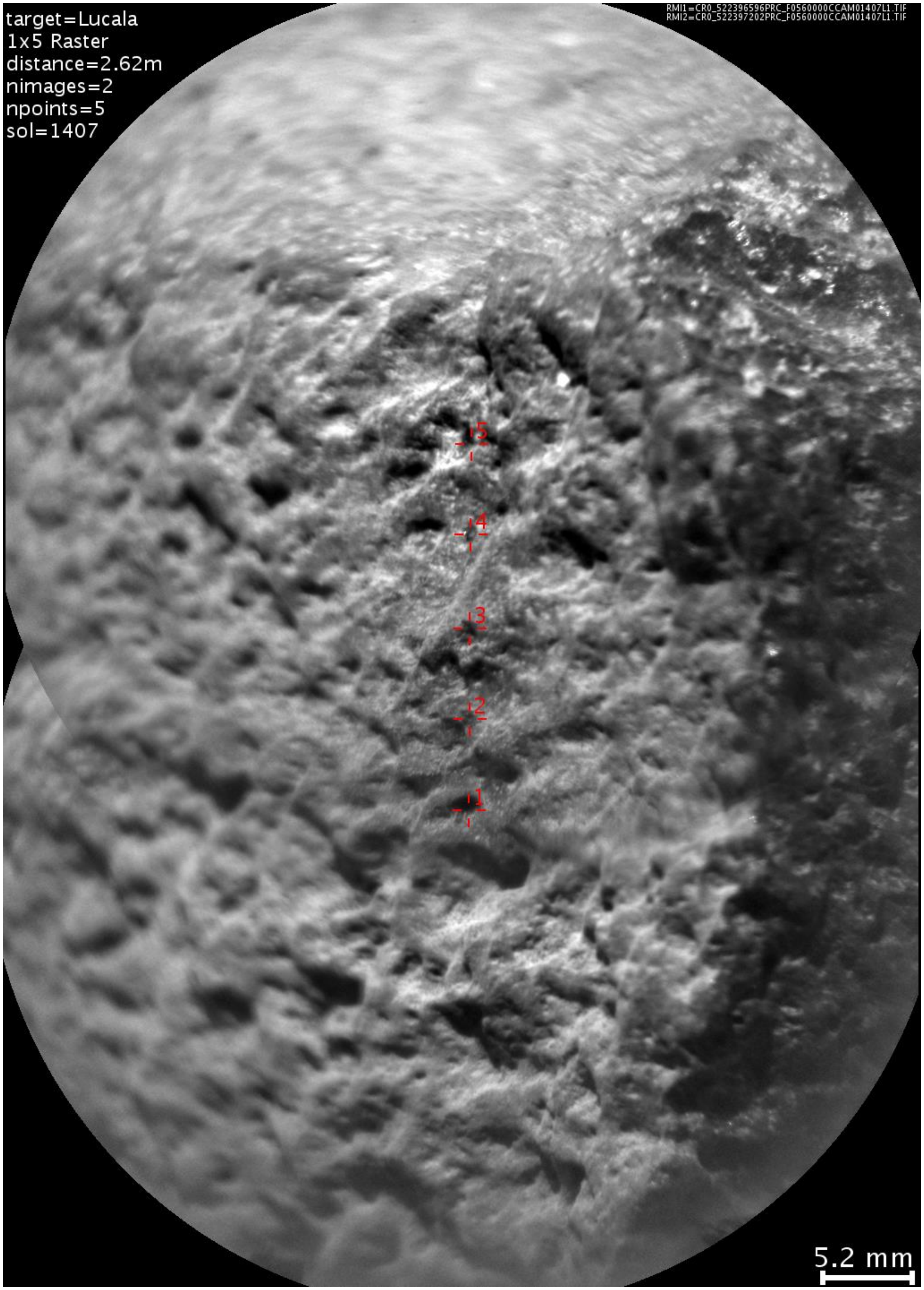

Lucala, RMI mosaic.

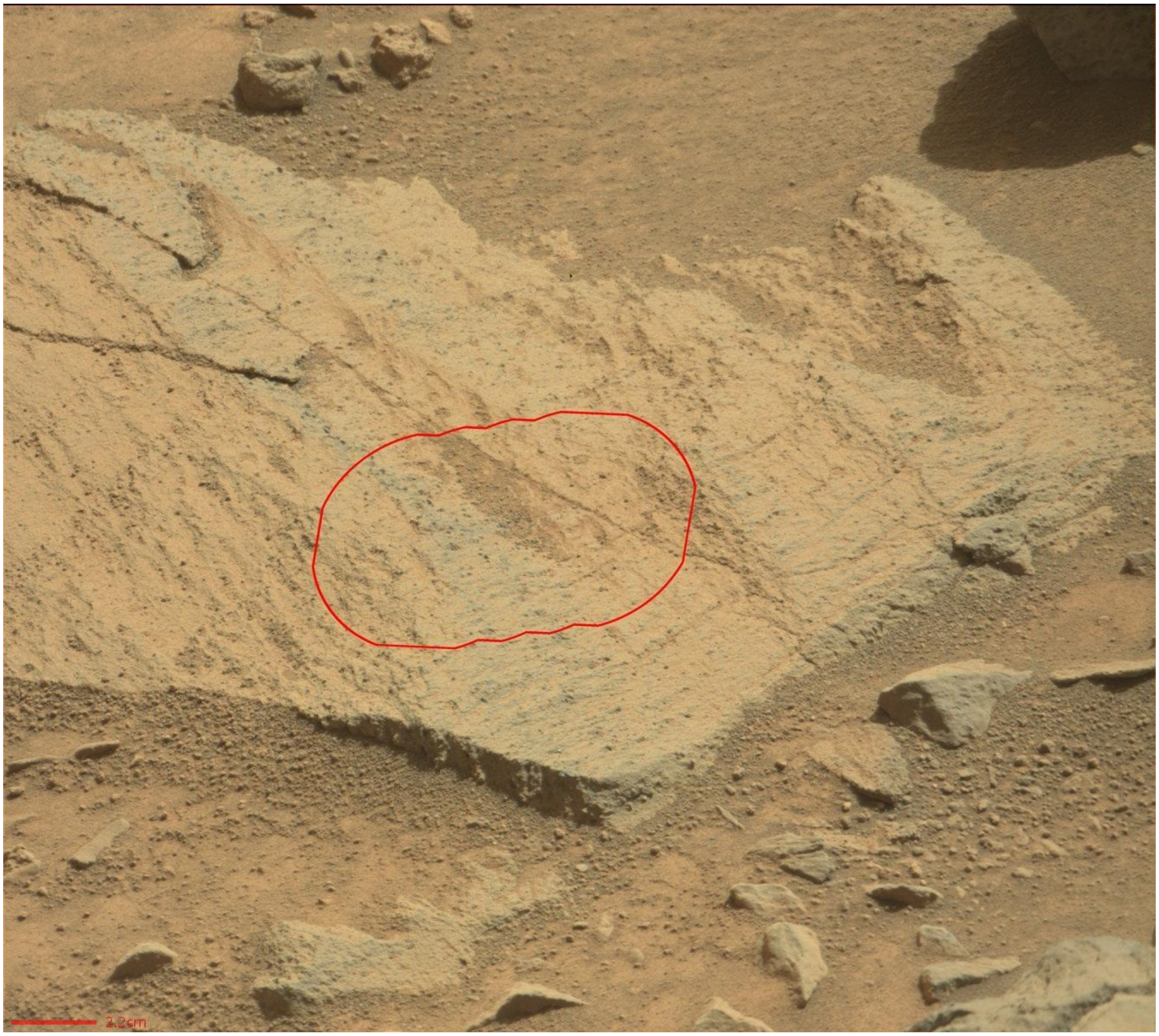

Jefferson, Mastcam image.

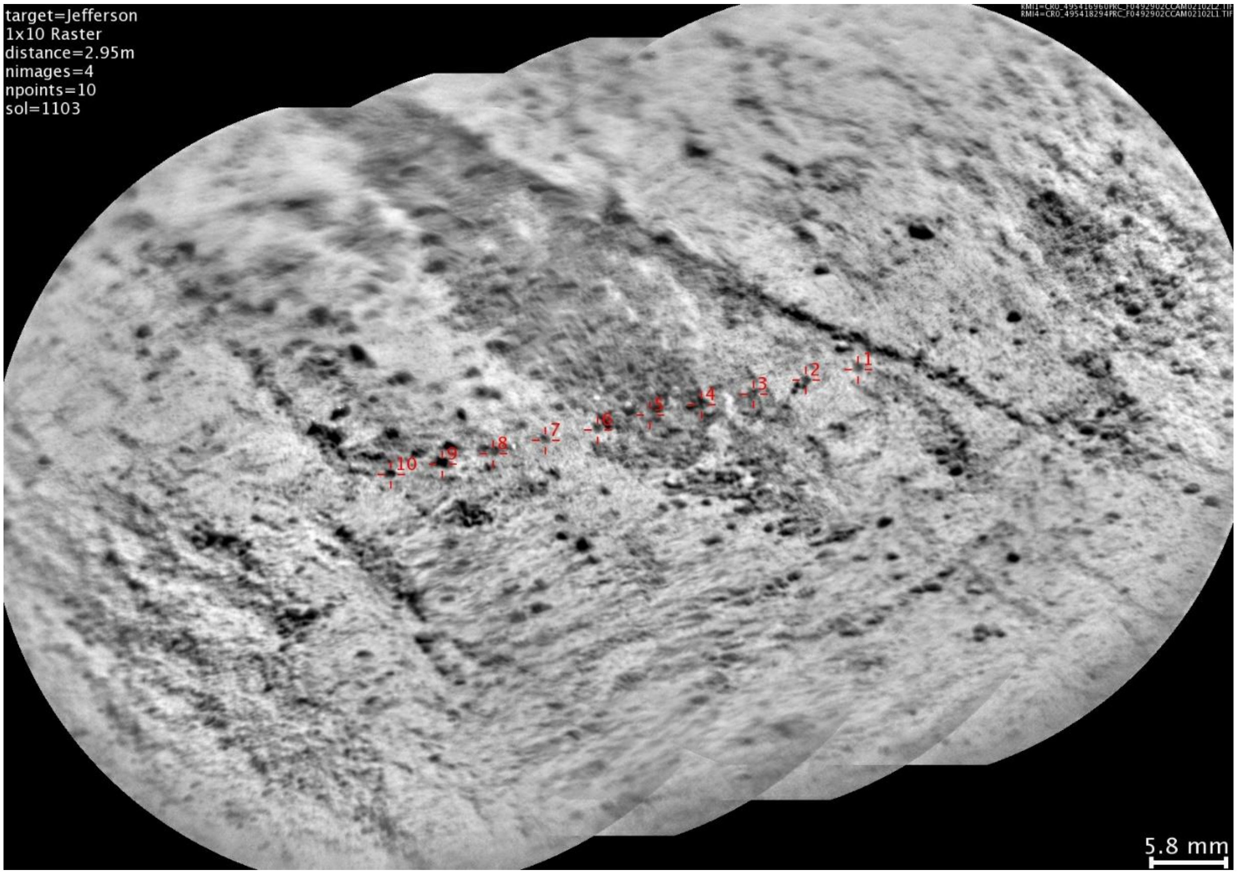

Jefferson, RMI mosaic.

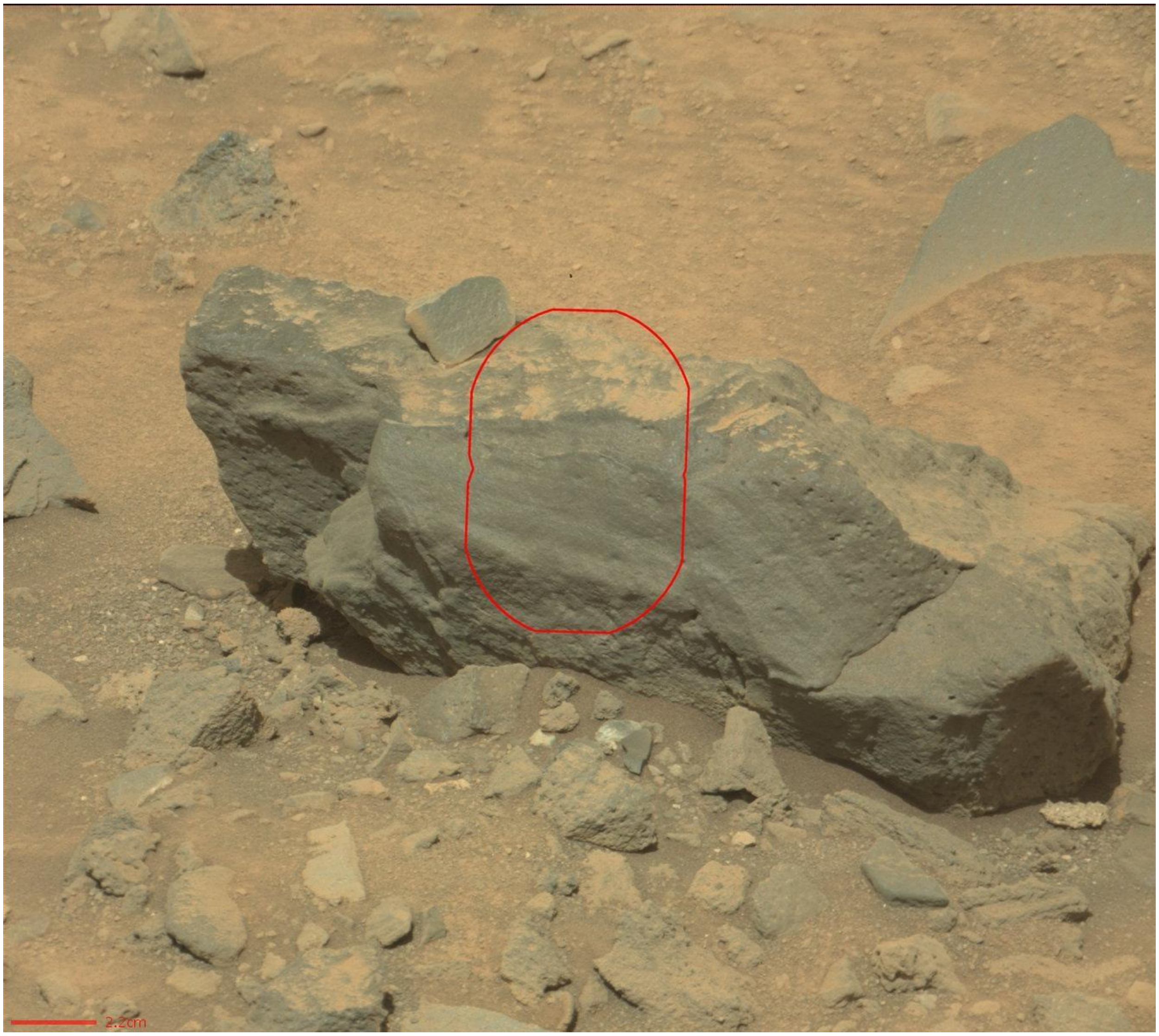

Lincoln, Mastcam image.

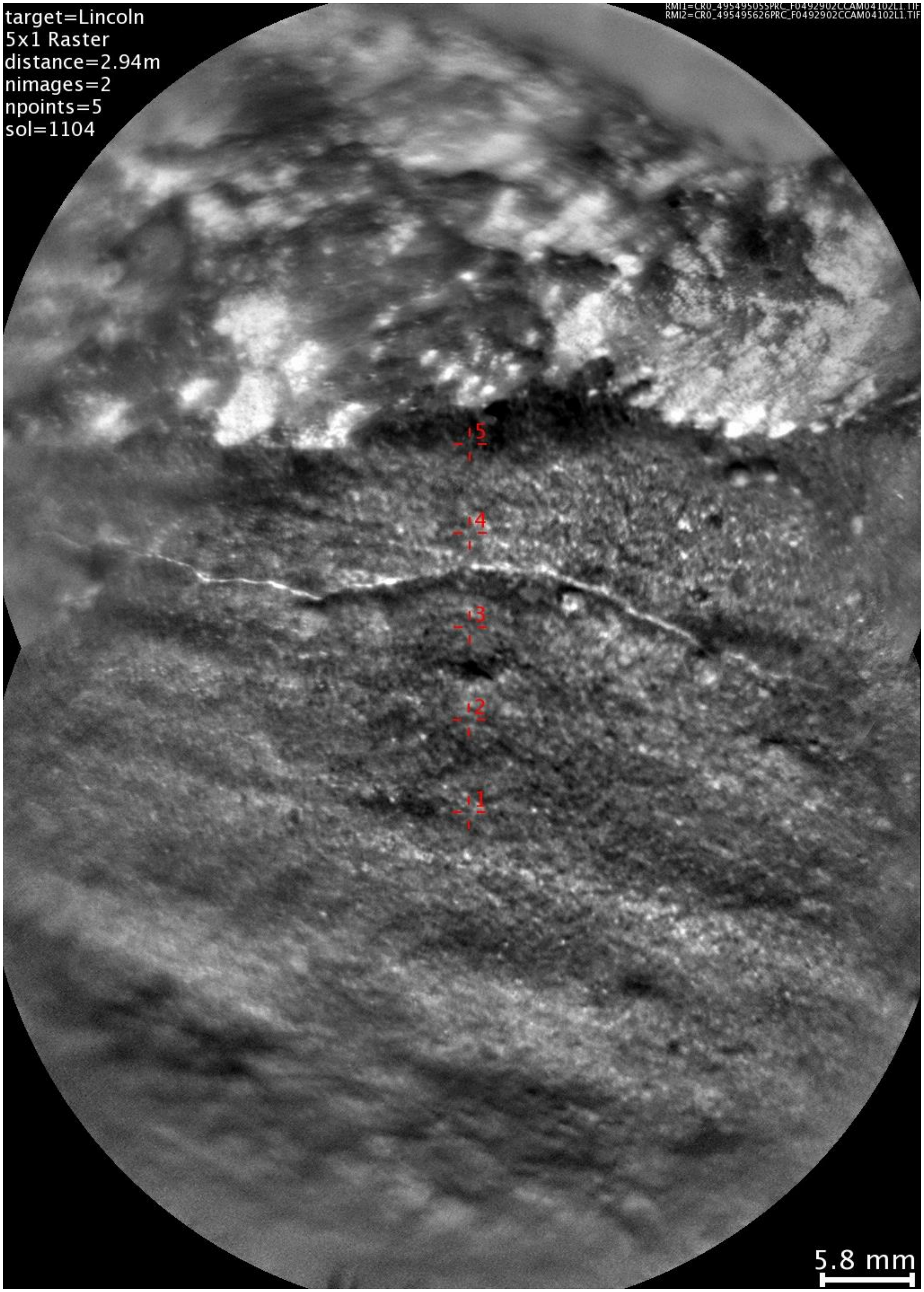

Lincoln, RMI mosaic

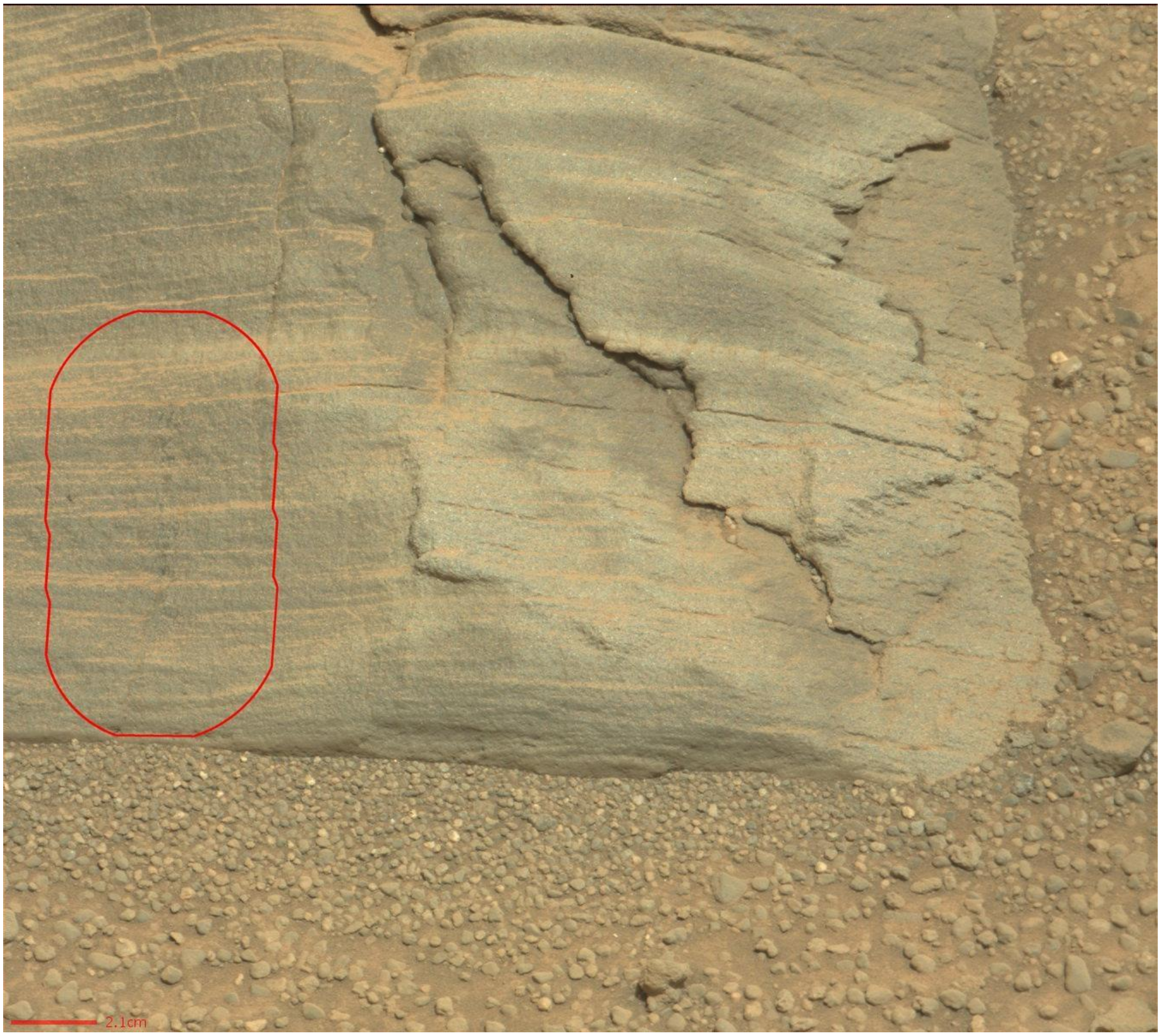

Swan, Mastcam image.

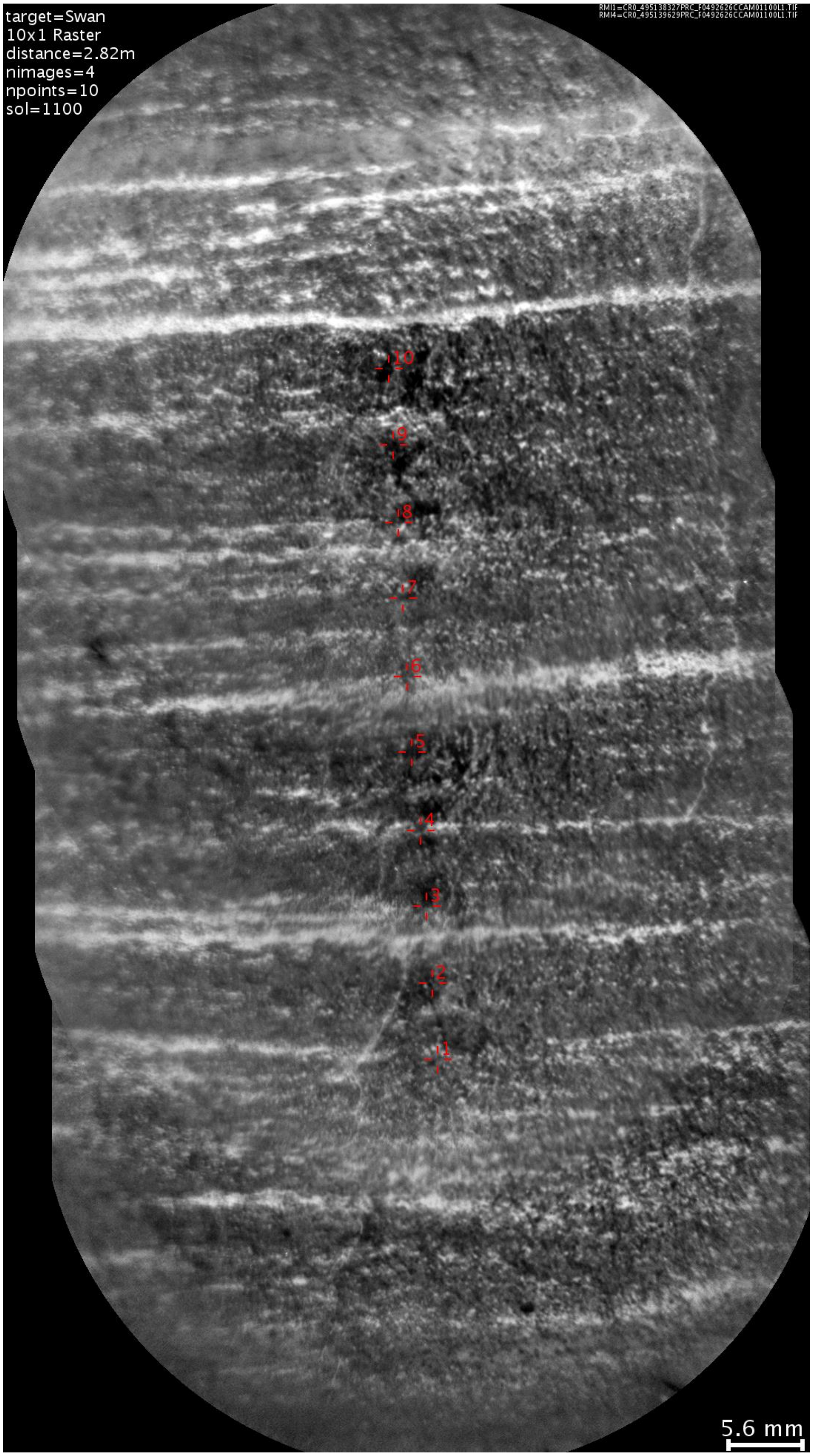

Swan, RMI mosaic.

11. Images of ChemCam Bradbury Targets Not in the Main Body of the Paper.

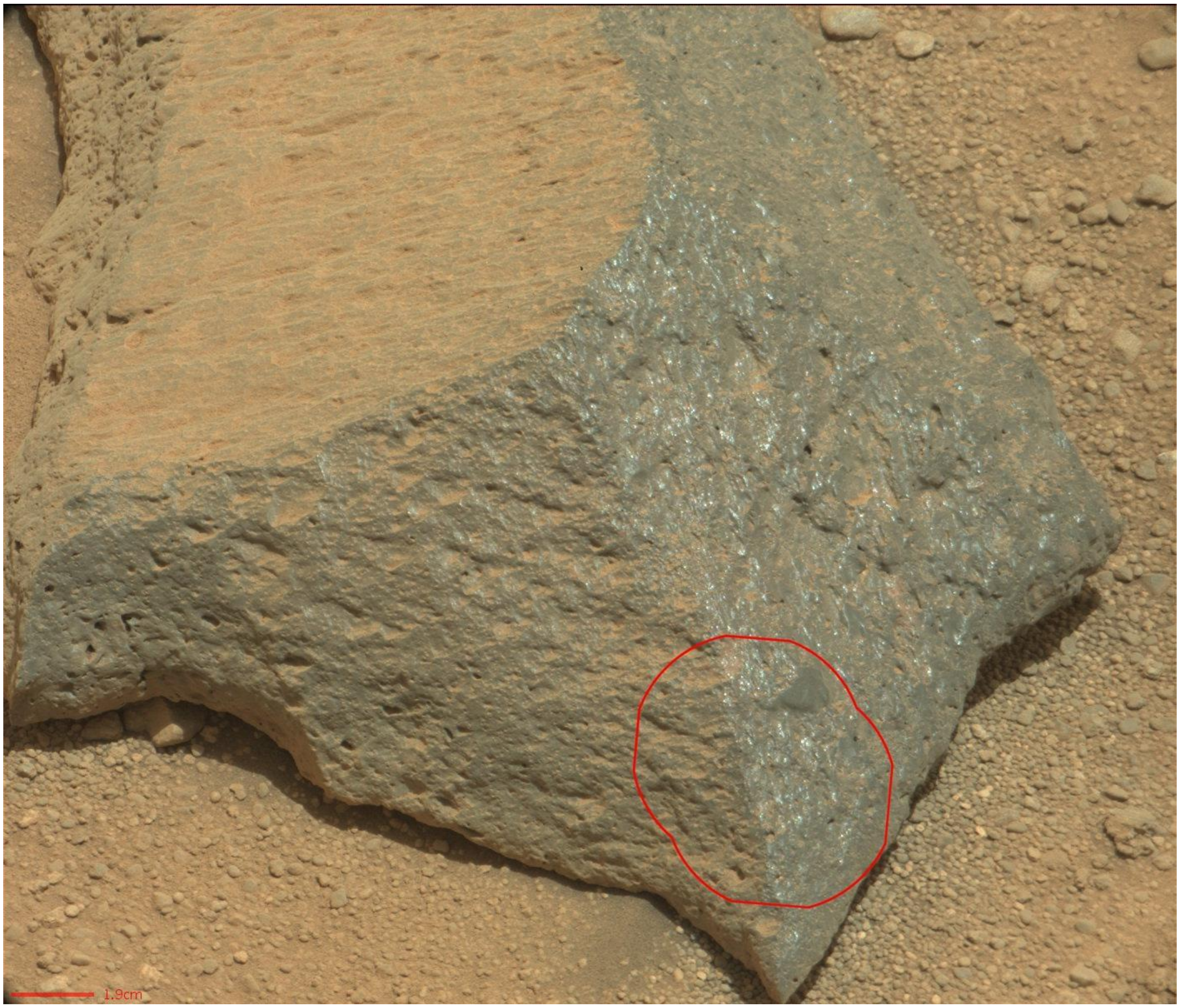

Black Trout, Mastcam Image

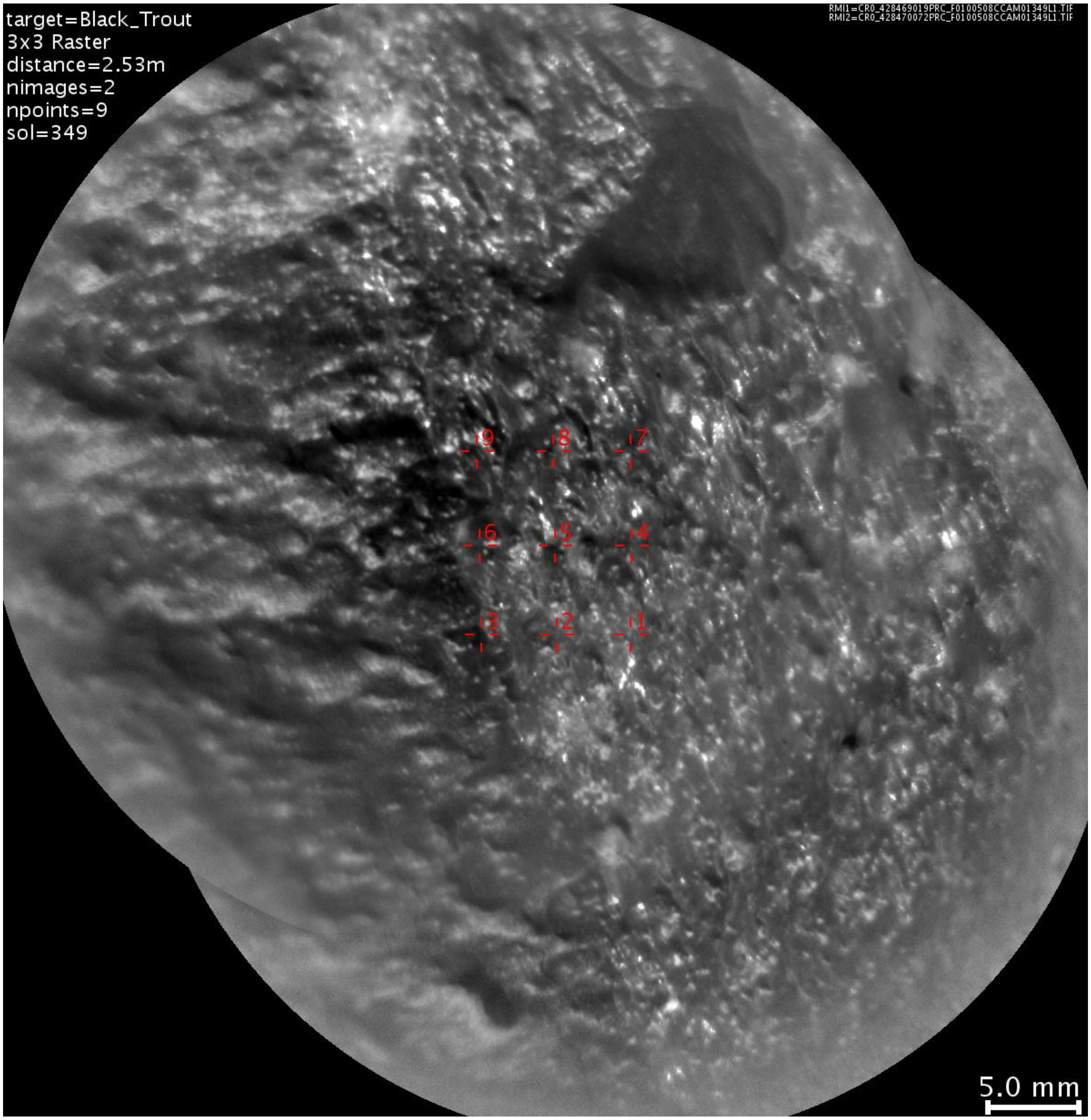

Black Trout, RMI mosaic.

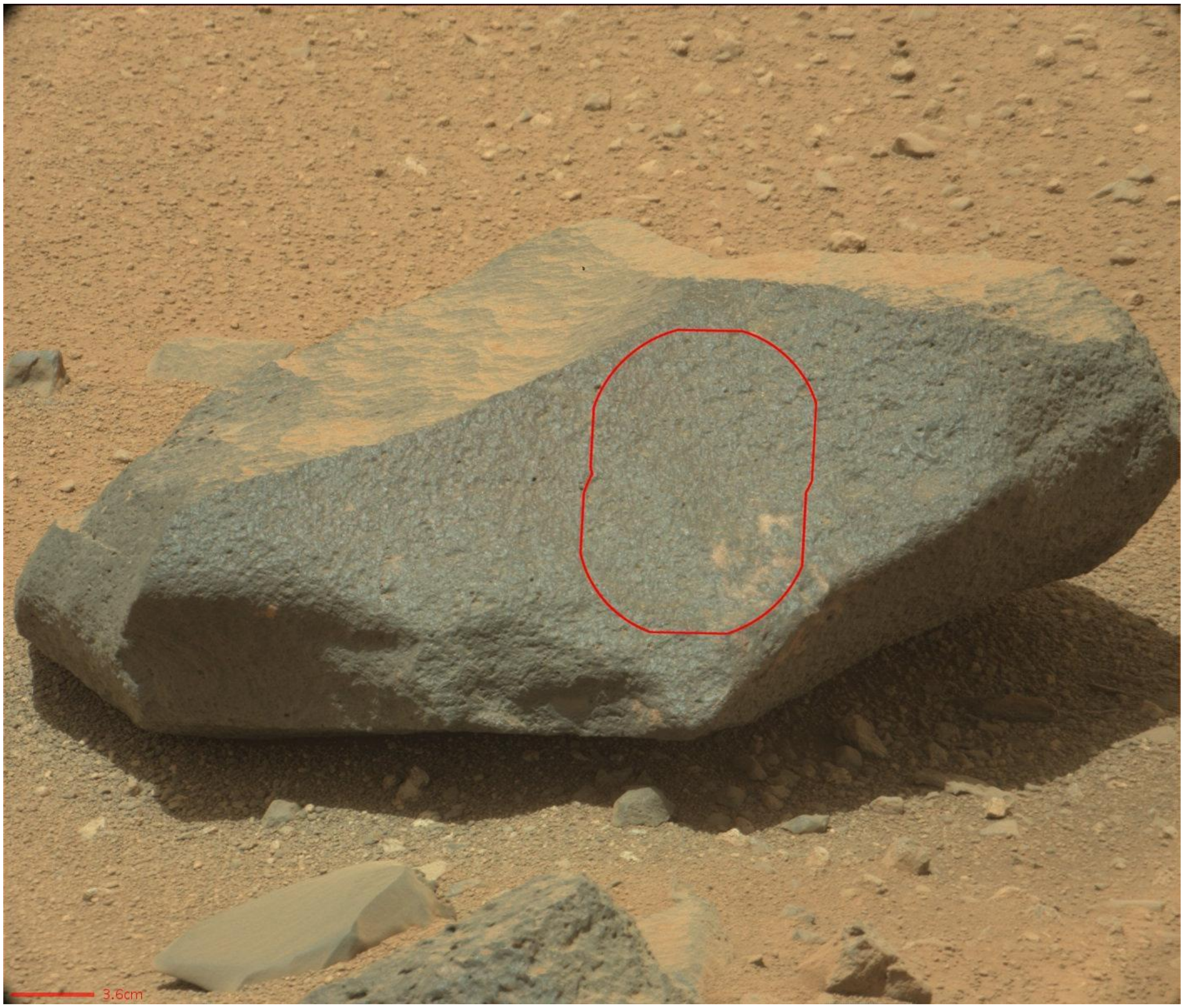

Mallard Lake, Mastcam image

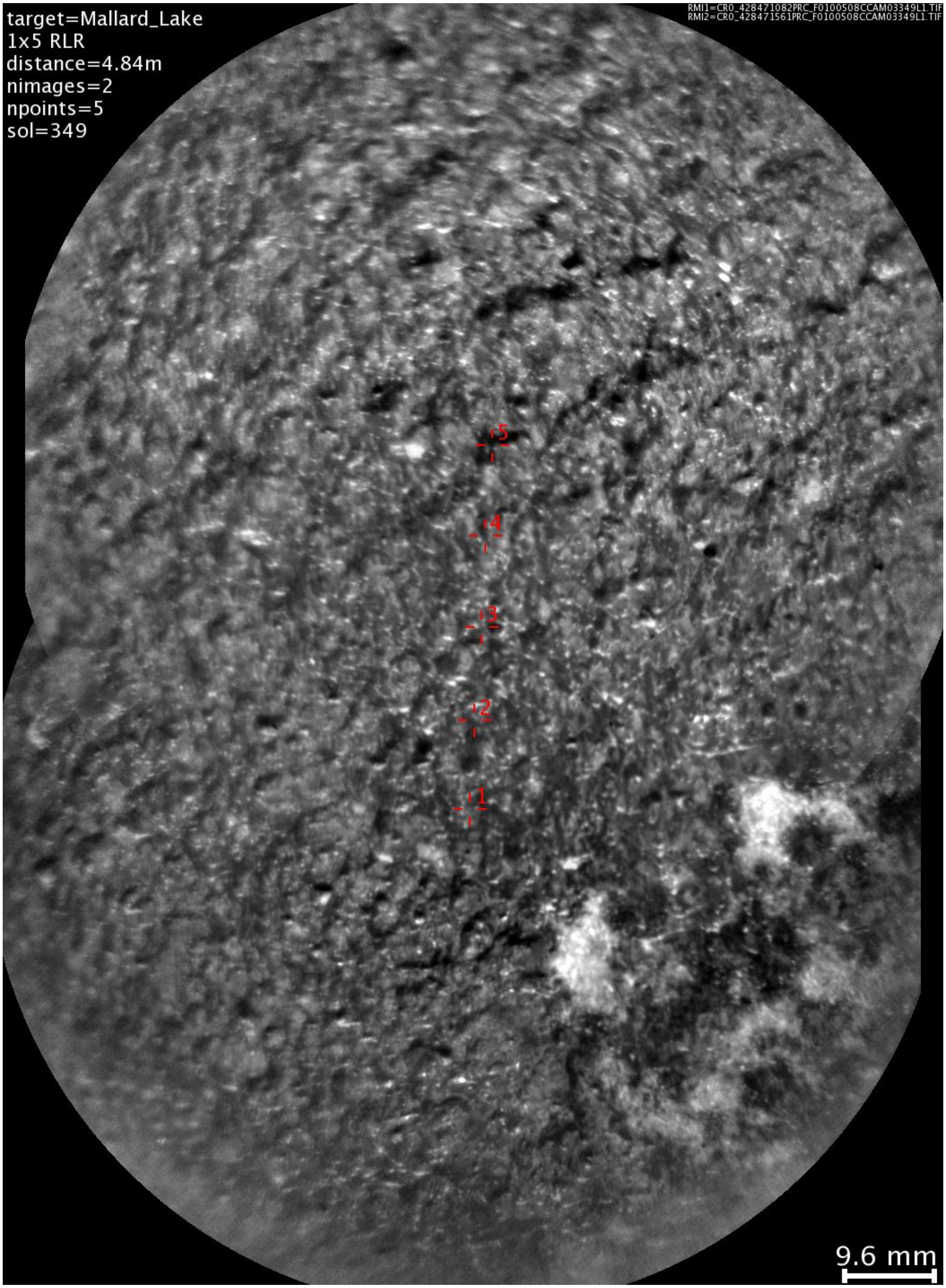

Mallard Lake, RMI mosaic.

12. Images of Other Targets from Zabriskie Plateau (Johnnie, South_Park2)

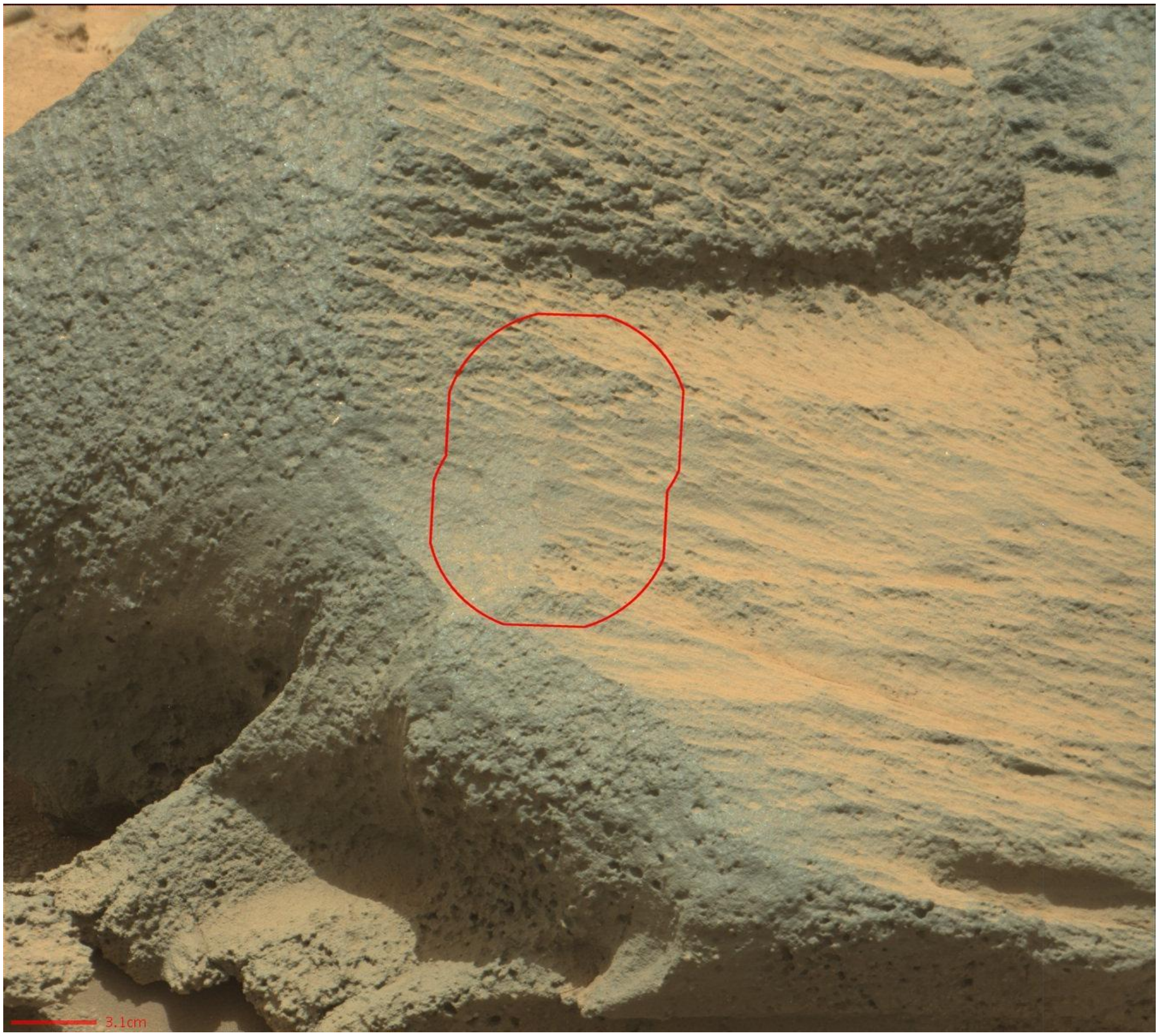

Johnnie, Mastcam image.

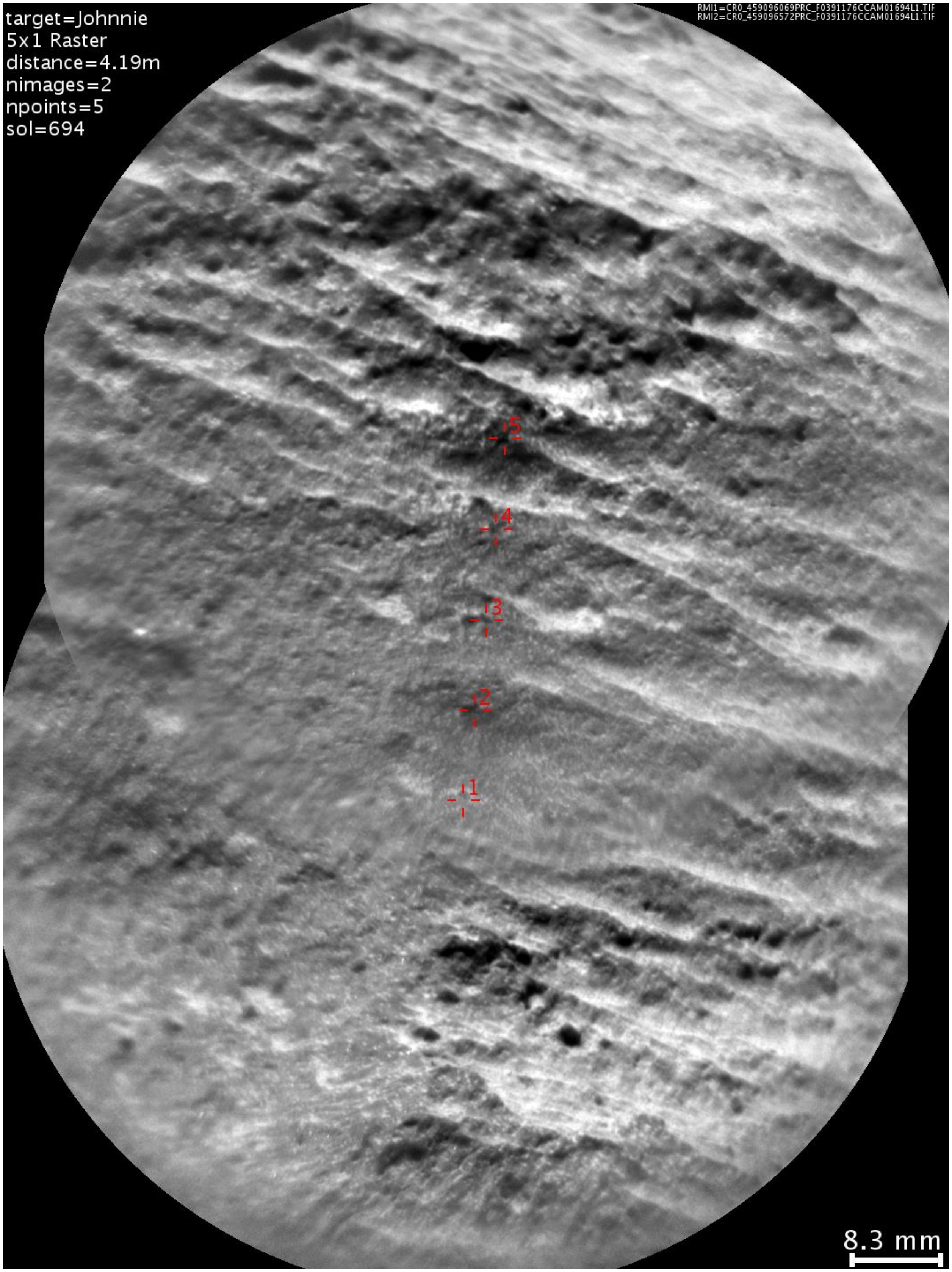

Johnnie, RMI mosaic.

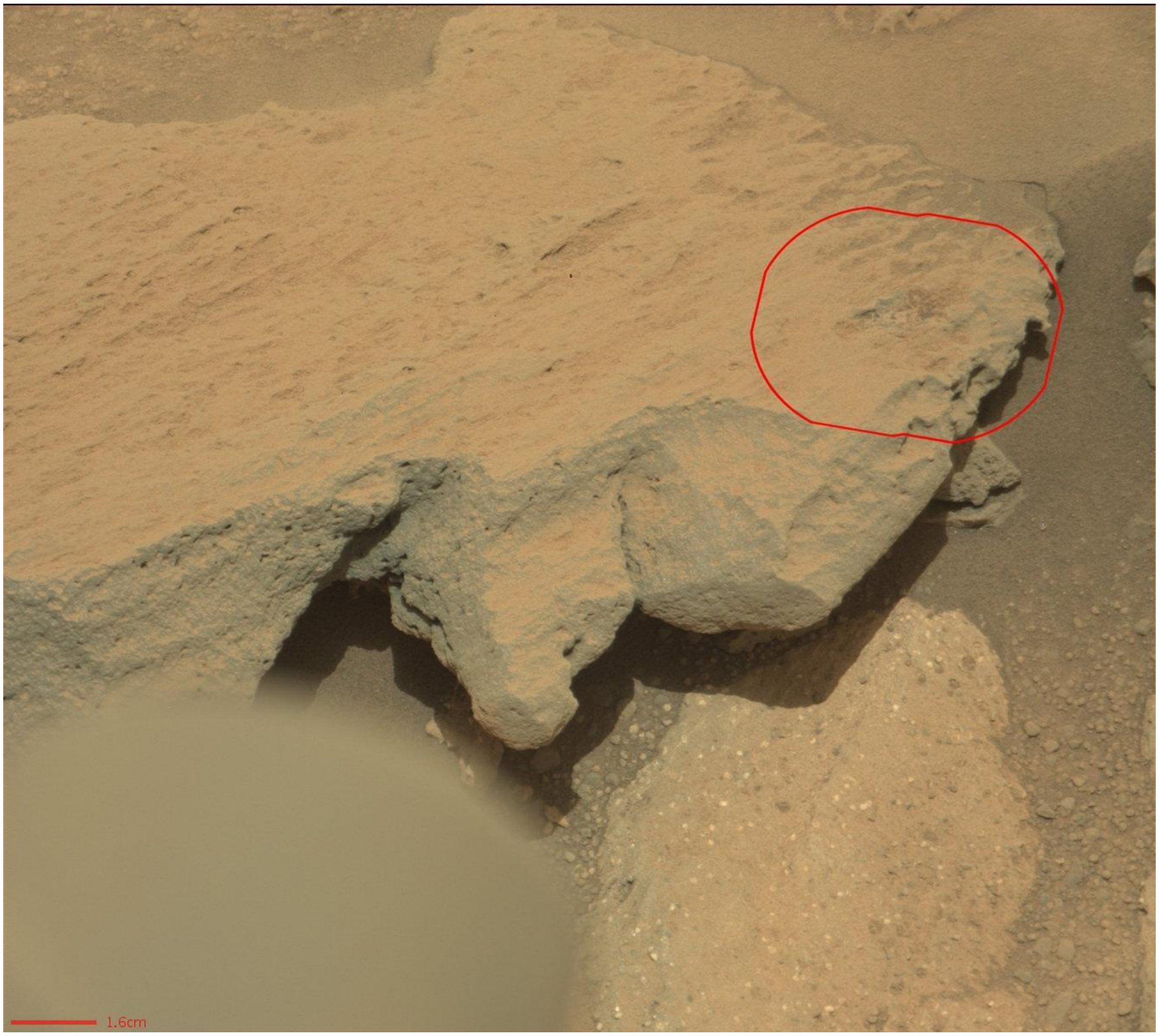

South_Park2, Mastcam image.

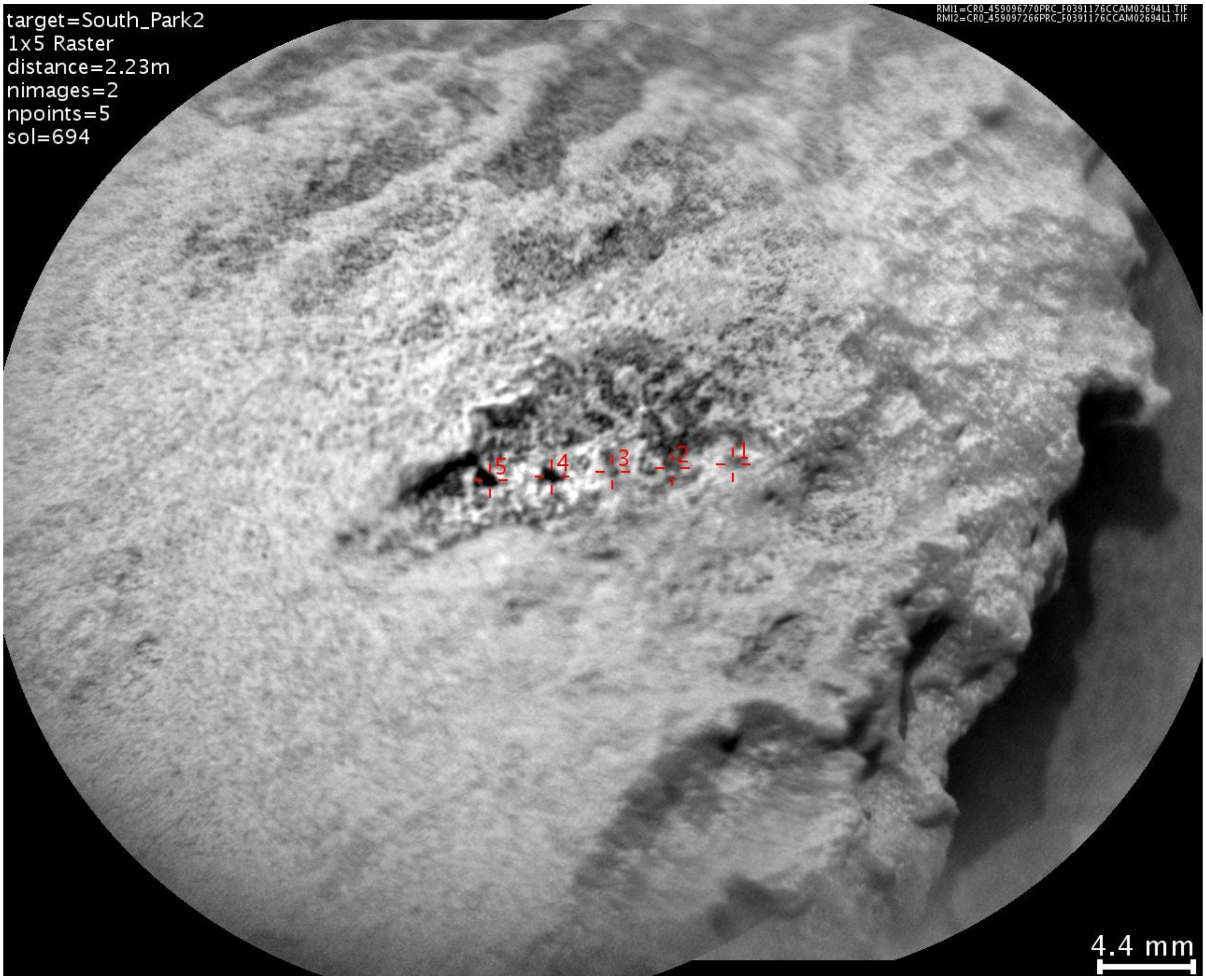

South_Park2, RMI image.

13. Designations of Images Used in the Paper

Fig. 1: Mosaic of images acquired by the MRO CTX and HiRISE cameras assembled by Calef and Parker (2016).

Fig. 2a: HiRISE image ESP_042682_1755, acquired on 04 September 2015.

Fig. 2b: Portion of Mastcam-34 image 1100ML0048710700500554C00.

Fig. 2c: Portion of Mastcam-34 image 1100ML0048710620500546C00.

Fig. 2d-e: Portions of a Mastcam-34 Sol 1094 mosaic (sequence mcam04850).

Fig. 2f: Portion of a Mastcam-100 Sol 1099 mosaic (sequence mcam04868).

Fig. 2g: Portion of Sol 1098 Navcam Left-B camera product NLB_494970804ILTLF0492374NCAM00266M1.

Fig. 2h: Portion of Sol 1104 MAHLI landscape image 1104MH0003250050401144E01.

Fig. 2i: Portion of a Mastcam-34 mosaic acquired on Sol 1100 (sequence mcam04871).

Fig. 3a: Portion of a Mastcam-34 mosaic acquired on Sol 1100 (sequence mcam04871).

Fig. 3b: Portion of Mastcam-100 image 1100MR0048770020600964E01.

Fig. 3c-d: Portions of a Mastcam-34 mosaic acquired on Sol 1100 (sequence mcam04871).

Fig. 3e: Portion of ChemCam RMI image product 1103_CRM_CCAM01102_Madison and a portion of Mastcam-100 image 1103MR0048880000601026E01.

Fig. 3f: Portion of MAHLI focus merge product 1103MH0005210000401138R00.

Fig. 3g: Portion of Mastcam-34 image 1100ML0048711100500594C00.

Fig. 4a: Portion of HiRISE image ESP_043539_1755 acquired on MSL Sol 1159 (10 November 2015).

Fig. 4b: Portion of a Sol 1163 Mastcam-34 mosaic (sequence mcam05263).

Fig. 4c: Portion of a Sol 1158 Mastcam-34 mosaic (sequence mcam05242).

Fig. 4d: Portion of Mastcam-34 image 1158ML0052420350502024C0.

Fig. 4e: Portion of a Sol 1160 Mastcam-100 mosaic (sequence mcam05247).

Fig. 4f: Anaglyph created from portions of HiRISE stereo pair images PSP_009149_1750 and PSP_009294_1750.

Fig. 5a: Portion of a Sol 1160 Mastcam-100 mosaic (sequence mcam05247).

Fig. 5b: ChemCam RMI image product 1160_CRM_CCAM2160_Hoba.

Fig. 5c: ChemCam RMI image product 1160_CRM_CCAM3160_Gibeon.

Fig. 5d: Portion of Mastcam-100 image 1160MR0052470570602057E01.

Fig. 5e: Portion of Mastcam-100 image 1277MR0059970040304226E01.

Fig. 5f: Portion of MAHLI focus merge product 1278MH0001700000500011R00.

Fig. 5g: Portion of a mosaic of Mastcam-100 images 1160MR0052470550602055E01 and 1160MR0052470620602062E01.

Fig. 5h: Portion of Mastcam-100 image 1160MR0052470310602031E01.

Fig. 5i: Portion of Mastcam-100 image 1160MR0052470600602060E01.

Fig. 5j: Portion of a Sol 1178 Mastcam-100 mosaic (sequence mcam05336).

Fig. 6: Portions of a HiRISE image mosaic constructed by Calef and Parker (2016).

Fig. 7: Base map is a portion of HiRISE image ESP_035917_1755. Insets for northeast Bimbe ridge and Bukalo are portions of a Sol 1387 Mastcam-100 mosaic (sequence mcam06815); inset for Bailundo is a portion of a Sol 1398 Mastcam-100 mosaic (sequence mcam06839).

Fig. 8: Stereo anaglyph constructed from portions of MRO HiRISE images PSP_009716_1755 and PSP_009650_1755.

Fig. 9a: Portion of a Sol 1402 Mastcam-100 mosaic (sequence mcam06869).

Fig. 9b: Portion of a mosaic of Navcam RDR image products:
NLB_522233603ILTLF0560000NCAM00353M1.IMG,
NLB_522233635ILTLF0560000NCAM00353M1.IMG,
NLB_522234307ILTLF0560000NCAM07753M1.IMG,
NLB_522234337ILTLF0560000NCAM07753M1.IMG,
NLB_522234369ILTLF0560000NCAM07753M1.IMG,
NLB_522234401ILTLF0560000NCAM07753M1.IMG,
NLB_522234590ILTLF0560000NCAM00654M1.IMG,
NLB_522234624ILTLF0560000NCAM00654M1.IMG, and
NLB_522234657ILTLF0560000NCAM00654M1.IMG.

Fig. 10a: Portion of a Sol 1407 Mastcam-34 mosaic (sequence mcam06889).

Fig. 10b: Portion of MAHLI image 1407MH0001900010502809C00.

Fig, 10c: Portion of MAHLI focus merge product 1408MH0001630000502842R00.

Fig. 11a: Composite of a portion of Sol 1405 Navcam Left-B product NLB_522234590ILTLF0560000NCAM00654M1 and Mastcam-100 images 1409MR0068990000702206E01 (Seeis) and 1409MR0069000010702208E01 (Aegis_1406a).

Fig. 11b: Portion of Mastcam-100 image 1409MR0068990000702206E01.

Fig. 11c: Portion of Mastcam-100 image 1409MR0069000010702208E01.

Fig. 11d: ChemCam RMI image product 1406_CRM_CCAM15900_aegis_post_1406a.

Fig. 12a-b: Portions of Mastcam-100 image 1408MR0068970460702184C00.

Fig. 12c: ChemCam RMI image product 1409_CRM_CCAM03409_Oranjemund.

Fig. 13a: Portion of Mastcam-34 image 1399ML0068480000601724E01.

Fig. 13b: Portion of Mastcam-100 image 1400MR0068560000702022E01.

Fig. 13c: ChemCam RMI image product 1400_CRM_CCAM01400_Auchab.

Fig. 14a: Portion of a Sol 1401 Mastcam-34 mosaic (sequence mcam06865).

Fig. 14b: Portion of Mastcam-100 image 1401MR0068610000702028E01.

Fig. 14c: ChemCam RMI image product 1401_CRM_CCAM03401_Chinchimane.

Fig. 15a: Portion of Sol 1405 Left-B Navcam image product NLB_522233635ILTLF0560000NCAM00353M1.

Fig. 15b: Portion of MAHLI image 1407MH0006270010502776C00.

Fig. 16a: Portion of a Sol 1401 Mastcam-34 mosaic (sequence mcam06865).

Fig. 16b: ChemCam RMI image product 1401_CRM_CCAM02401_Canico.

Fig. 16c -- Portions of a Sol 1401 Mastcam-34 mosaic (sequence mcam06865).

Fig. 17a: Portion of a Sol 1407 Mastcam-34 mosaic (sequence mcam06889).

Fig. 17b-c: Portions of a Sol 1408 Mastcam-100 mosaic (sequence mcam06898).

Fig. 18a: Portion of a Sol 1409 mosaic of Mastcam-100 and Mastcam-34 images (sequence mcam06889).

Fig. 18b: Portion of ChemCam RMI image product 1409_CRM_CCAM01409_Seeheim.

Fig. 18c: Portion of ChemCam RMI image product 1407_CRM_CCAM03407_Bungo.

Fig. 18d: Portion of ChemCam RMI image product 1407_CRM_CCAM02407_Cabamba.

Fig. 18e: Portion of ChemCam RMI image product 1409_CRM_CCAM02409_Wilhelmstal.

Fig. 19a: Portion of a mosaic of MAHLI images 1407MH0006270010502774C00 and 1407MH0006270010502776C00.

Fig. 19b: Portion of MAHLI focus merge product 1411MH0005840000503006R00.

Fig. 19c-d: Portions of MAHLI focus merge product 1411MH0005840000503000R00.

Fig. 20a: Portion of Mastcam-100 image 1410MR0069050010702210E01. Inset is a portion of a Sol 1402 Mastcam-100 mosaic (sequence mcam06869).

Fig. 20b: ChemCam RMI image product 1410_CRM_CCAM05409_Mariental.

Fig. 22a: Portion of Mastcam-34 image 0052ML0002400070102217E01; inset is a composite of MAHLI images 0054MH0000160010100280C00 and 0054MH0000180010100312C00,

Fig. 22b: Portion of Mastcam-100 image 0336MR0013560000301059E01.

Fig. 22c: Portion of Mastcam-100 image 0349MR0014160000301161E01.

Fig. 22d: Portion of a mosaic of Mastcam-100 images 0044MR0002040290102567E01, 0044MR0002040220102560E01, 0044MR0002040150102553E01, and 0044MR0002040080102546E01.

Fig. 22e: Portion of Mastcam-100 image 0516MR0020340000303244E01; inset is a portion of MAHLI focus merge product 0516MH0002650000200986R00.

Fig. 22f: Portion of Mastcm-100 image 0692MR0029280000402215E01.
